# FNDC4 acts as an anti-inflammatory factor on macrophages and improves colitis in mice

**DOI:** 10.1038/ncomms11314

**Published:** 2016-04-12

**Authors:** Madeleen Bosma, Marco Gerling, Jenny Pasto, Anastasia Georgiadi, Evan Graham, Olga Shilkova, Yasunori Iwata, Sven Almer, Jan Söderman, Rune Toftgård, Fredrik Wermeling, Elisabeth Almer Boström, Pontus Almer Boström

**Affiliations:** 1 Department of Cell and Molecular Biology, Karolinska Institutet, Stockholm SE-171 77, Sweden; 2 Department of Biosciences and Nutrition, Center of Innovative Medicine, Karolinska Institutet, Huddinge SE-141 83, Sweden; 3 Division of Nephrology, Kanazawa University Hospital, Kanazawa 920-8641, Japan; 4 Department of Medicine, Solna, Karolinska Institutet, and Karolinska University Hospital, Stockholm SE-171 76, Sweden; 5 GastroCentrum, Karolinska University Hospital, Solna, Stockholm SE-171 76, Sweden; 6 Division of Medical Diagnostics, Ryhov County Hospital, Jönköping 55185, Sweden; 7 Department of Dental Medicine, Karolinska Institutet, Huddinge SE-141 83, Sweden

## Abstract

FNDC4 is a secreted factor sharing high homology with the exercise-associated myokine irisin (FNDC5). Here we report that *Fndc4* is robustly upregulated in several mouse models of inflammation as well as in human inflammatory conditions. Specifically, *FNDC4* levels are increased locally at inflamed sites of the intestine of inflammatory bowel disease patients. Interestingly, administration of recombinant FNDC4 in the mouse model of induced colitis markedly reduces disease severity compared with mice injected with a control protein. Conversely, mice lacking *Fndc4* develop more severe colitis. Analysis of binding of FNDC4 to different immune cell types reveals strong and specific binding to macrophages and monocytes. FNDC4 treatment of bone marrow-derived macrophages *in vitro* results in reduced phagocytosis, increased cell survival and reduced proinflammatory chemokine expression. Hence, treatment with FNDC4 results in a state of dampened macrophage activity, while enhancing their survival. Thus, we have characterized FNDC4 as a factor with direct therapeutic potential in inflammatory bowel disease and possibly other inflammatory diseases.

## Abstract (web summary)

FDNC4 is a poorly characterized homologue of FNDC5/irisin, a myokine induced by exercise. Here the authors show that FDNC4 increases macrophage survival in growth factor deprivation, inhibits phagocytosis and transcriptional responses to M1 and M2 polarizing stimuli, and protects mice from DSS-induced colitis.

## Graphical abstract

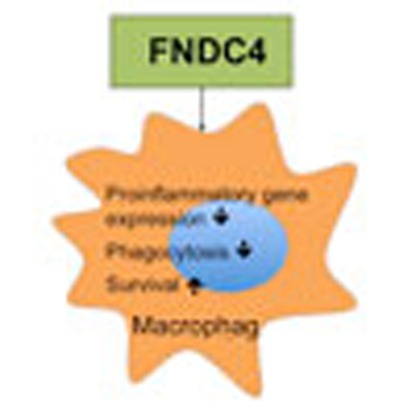

The incidence and prevalence of Crohn's disease and ulcerative colitis, the two major forms of inflammatory bowel disease (IBD), are rising globally^1^. IBD is characterized by inflammation of the gastrointestinal tract of genetically susceptible individuals exposed to environmental factors^2^. The hallmark of the pathogenesis is a hyperactive and deregulated immune function in the bowel, where macrophages play a central role^3^. Treatment possibilities are limited and often come at the cost of potentially severe side effects^4^. Therefore, further knowledge on the molecular pathology and the development of novel treatment approaches is highly warranted.

We have characterized a novel secreted factor, FNDC4, a member of the fibronectin type III domain family of proteins. This protein family consists of five proteins (FNDC1-5), of which FNDC4 shows the strongest homology with FNDC5 (ref. 5). FNDC5/irisin was recently identified as an exercise-induced secreted factor targeting adipose tissue to induce brown fat formation and energy expenditure^6^. Irisin is released from skeletal muscle in response to exercise by cleavage of its precursor, the type 1 membrane protein FNDC5 (refs 6, 7). Conclusive data now show that irisin protein circulates in humans, at concentrations typical for hormones^8^. Irisin is a soluble polypeptide consisting primarily of one fibronectin type III domain^6^,^7^,^8^. Among the members of proteins containing the fibronectin type III domain, this domain architecture is only found in one additional gene: *Fndc4* (also known as *Frcp1*) (ref. 5). FNDC4 protein shares 57% amino acid identity with FNDC5 in the functional, extracellular domain. Like *Fndc5*, *Fndc4* is highly conserved (excluding the signal peptide, human *FNDC4* is 100% homologous to mouse *Fndc4*). *Fndc4* expression is highest in liver and brain, but it is expressed at lower levels in many other tissues, including heart, skeletal muscle and adipose tissue.

The observation that *Fndc4*, but not *Fndc5*, is robustly regulated in the inflamed state led us to investigate its potential role in immunomodulation. Here we show that administration of FNDC4 results in reduced inflammation and disease severity in a mouse model for IBD, indicating that FNDC4 could have therapeutic potential. FNDC4 binds to and induces signalling in one key inflammatory cell; the macrophage. Interestingly, FNDC4 fundamentally alters the activation state of macrophages, which has important consequences for macrophage function.

## Results

### FNDC4 is cleaved and secreted

**Figure 1:**
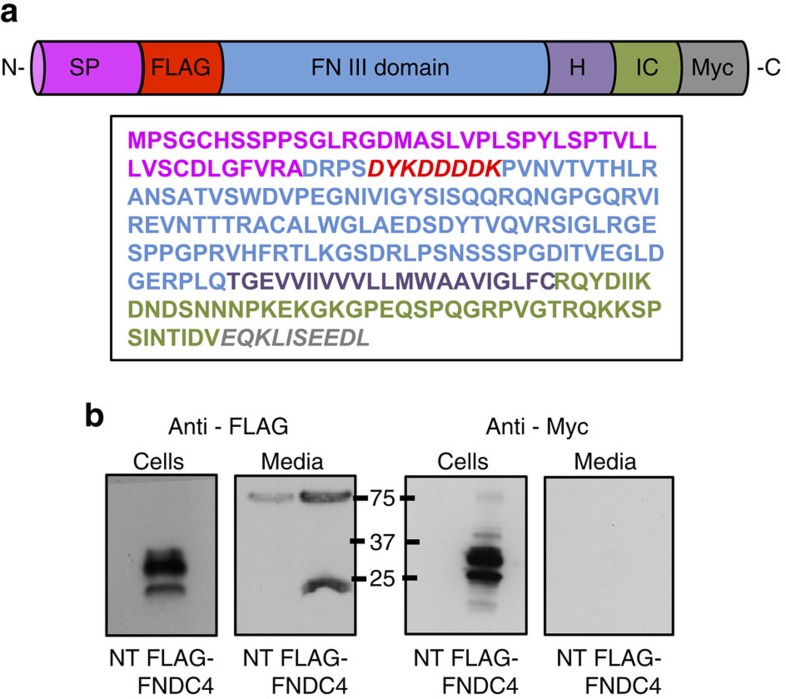
An N-terminal portion of FNDC4 is secreted. (**a**) Graphical representation of the FNDC4 construct with N-terminal FLAG and C-terminal Myc tags as well as the peptide sequence. C, intracellular domain; FN III domain, Fibronectin type III domain; H, hydrophobic domain; SP, signal peptide. (**b**) Western blot against either FLAG or Myc, as indicated, in cell- and media samples from HEK293 cells transfected with the N-terminal FLAG and C-terminal Myc FNDC4 construct. The full-size blot image is presented in Supplementary Fig. 11a. Similar results were seen in at least three independent experiments. NT, non-transfected control.

Given its similarity to FNDC5, we investigated whether FNDC4 is cleaved to release a secreted part similar to FNDC5 processing. For this purpose, an N-terminal FLAG-tagged and C-terminal Myc-tagged FNDC4 construct was synthesized (Fig. 1a). After overexpression of the construct in HEK293 cells, a clear FLAG-positive protein band was identified in both cells and media when probing the N-terminal FLAG tag using western blot (Fig. 1b, left panel). Probing against Myc, however, resulted in a detectable band only in the cellular fraction (Fig. 1b, right panel). Thus, an N-terminal portion of FNDC4 is released from the cells in accordance with the release mechanism of FNDC5/irisin^6^,^7^. Importantly, the band in the media fraction was of reduced MW compared with the cellular version, signifying a cleaved version. Thus, the N-terminus of FNDC4 can be shredded to release an extracellular portion of the protein.

### *Fndc4* is upregulated in settings of inflammatory disease

**Figure 2:**
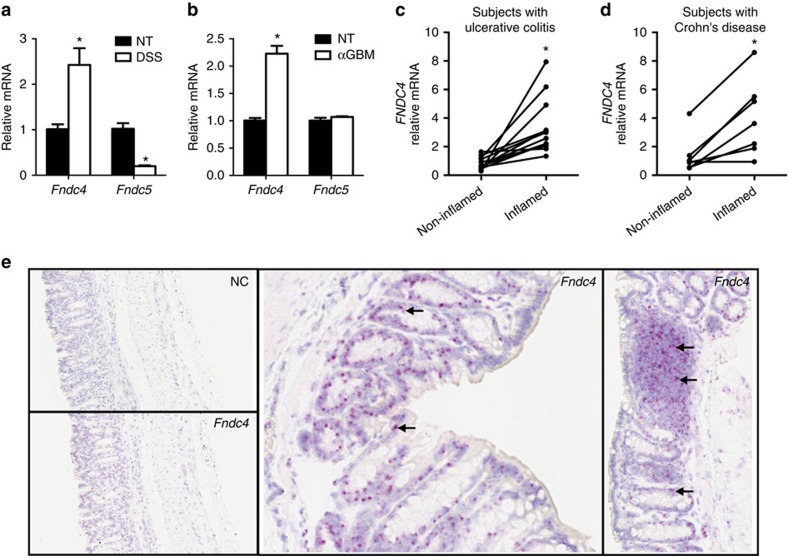
*Fndc4* gene expression is upregulated in inflammatory conditions. (**a**) *Fndc4* gene expression in the murine colon on induction of DSS-mediated colitis. DSS was administered via the drinking water, mice were sacrificed 7 days later, *N*=10 mice per group. NT, non-treated. Mean±s.e.m., *P<0.05 (Student's *T*-test). This experiment was performed three times with similar results. (**b**) *Fndc4* gene expression in the kidney on induction of glomerulonephritis. Mice were injected with antiserum directed against the glomerular basement membrane (αGBM) and sacrificed 5 days post injection. NT, non-treated. *N*=5 mice per group. Mean±s.e.m., *P<0.05 (Student's *T*-test). This experiment was repeated once with similar results. (**c**–**d**) Human IBD cohort. *FNDC4* gene expression in intestinal biopsies from inflamed versus non-inflamed regions of the same patients with ulcerative colitis (UC) (*N*=12) (**c**) or Crohn's disease (CD) (*N*=7) (**d**). *P<0.05, paired *T*-tests. (**e**) RNA *in situ* hybridization. Distal colon from DSS-treated mice. *Fndc4* versus negative control (NC) probe. Arrows point at examples of positive signals (red).

To explore the potential roles of FNDC4 and FNDC5 in inflammation, we first studied the expression of both *Fndc4* and its close homologue *Fndc5* in three models of inflammation; (i) dextran sodium sulfate (DSS)-induced inflammation of the colon, (ii) autoimmune inflammation of the kidney and (iii) ischaemia-induced inflammation of the kidney. Histology and gene expression data showing the degree of inflammation are presented in Supplementary Fig. 1. Interestingly, in all three models, *Fndc4* gene expression was upregulated, whereas *Fndc5* was either downregulated or unchanged (Fig. 2a,b and Supplementary Fig. 2a,b). Moreover, with ischaemia reperfusion, the regulation of *Fndc4* was seen both locally in the ischaemic kidney (Supplementary Fig. 2a), as well as in the liver (Supplementary Fig. 2c). In contrast to *Fndc5*, *Fndc4* is not regulated by exercise training, at least not at the transcriptional level (Supplementary Fig. 2d,e). We concluded that *Fndc4* and *Fndc5* have clearly different expression profiles when comparing exercise and inflammation. Given the consistent upregulation of *Fndc4* in inflammation and the fact that FNDC4 shows superior protein stability when produced as a recombinant protein compared with irisin/FNDC5, we focused subsequent studies on this molecule.

### Subjects with IBD display higher *FNDC4* expression

To investigate whether the inflammation-induced regulation of *Fndc4* in mice translates to humans, we analysed *FNDC4* and *FNDC5* expression in patients with IBD and controls without intestinal inflammation. Intestinal biopsies obtained during ileocolonoscopy from 52 IBD patients (21 with Crohn's disease, 29 with ulcerative colitis and 2 with unclassified IBD, Supplementary Table 2) and 19 controls were analysed for *FNDC4* and *FNDC5* expression using quantitative reverse transcription–PCR (RT–qPCR). Paired samples from both non-inflamed and inflamed regions were available from 21 IBD patients (7 with Crohn's disease, 12 with ulcerative colitis and 2 with unclassified IBD). *FNDC5* gene expression was undetectable in the intestinal biopsies. No gender differences or regional differences between ileum and colon or within different segments of the colon in *FNDC4* expression were observed (*P*=0.125; *T*-test, and *P*=0.507; one-way analysis of variance, respectively, Supplementary Fig. 3a,b). No differences were seen in *FNDC4* expression between non-IBD controls and non-inflamed samples from IBD patients (*P*=0.370; *T*-test, Supplementary Fig. 3c). Yet interestingly, *FNDC4* expression was significantly increased in inflamed regions of the intestine when compared with non-inflamed regions from the same subjects in both ulcerative colitis (*P*=0.002; paired *T*-test, Fig. 2c) and Crohn's disease (*P*=0.007; paired *T*-test, Fig. 2d). Furthermore, among inflamed samples from IBD patients, *FNDC4* gene expression correlated with *TNF* (*r*=0.654, *P*<0.0001; Pearson's correlation coefficient; Supplementary Fig. 3d) and *CCL2* (MCP1) gene expression (*r*=0.612, *P*<0.0001; Pearson's correlation coefficient; Supplementary Fig. 3e), further supporting the notion that *FNDC4* is regulated with inflammation.

### *Fndc4* is expressed in colonic epithelium and immune cells

To identify the cellular sources of *Fndc4* in the inflamed state, we applied RNA *in situ* hybridization using the high-sensitivity RNAscope technology^9^. Signal analysis revealed that in the murine colon, *Fndc4* mRNA is expressed in the epithelium and in a subset of immune cells in lymphoid aggregates (Fig. 2e, right panel). We further investigated *Fndc4* gene expression in different immune cell types using publicly available data sets^10^ (Supplementary Fig. 4). This revealed that various human and mouse immune cell types showed low but detectable *FNDC4* gene expression (Supplementary Fig. 4a–b). *FNDC4* expression tended to be increased in activated human neutrophils (Supplementary Fig. 4a). In mice, natural killer cells and dendritic cells showed the highest levels of *Fndc4* expression (Supplementary Fig. 4b). Interestingly, human monocytes showed higher *FNDC4* expression levels compared with macrophages (Supplementary Fig. 4c). Yet, expression in immune cells is generally low compared with colonic epithelial cells. In macrophages, *Fndc4* gene expression is close to the detection limit with RT-qPCR, whereas Caco-2 colonic epithelial cells show expression levels that are several orders of magnitude higher (Supplementary Fig. 4d). Thus, *Fndc4* is expressed in colonic epithelial cells and several immune cell subtypes.

### *Fndc4* expression is regulated by TGF-β

**Figure 3:**
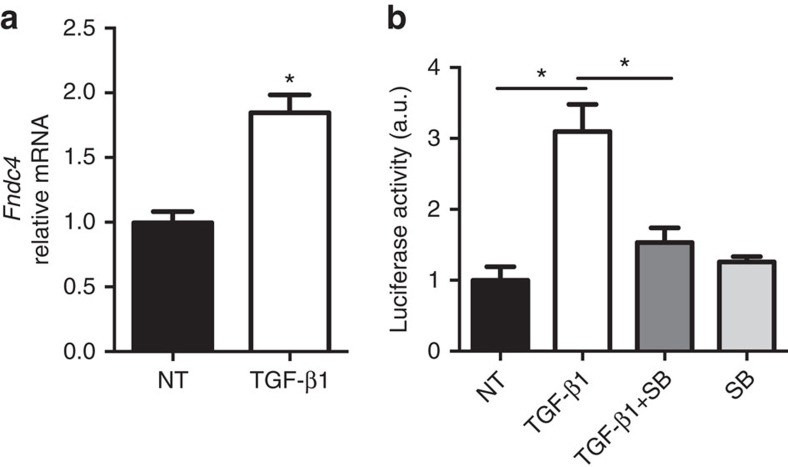
TGFβ induces *FNDC4* expression. (**a**) *FNDC4* expression in Caco-2 cells after 20 h treatment with 20 ng ml^−1^ TGFβ1. *N*=4. Mean±s.e.m., *P<0.05, Student's *T*-test. (**b**) Luciferase assay. McA-RH7777 cells were transfected with an *Fndc4* promoter reporter construct and treated with 20 ng ml^−1^ TGFβ1 and/or TGFβ1R antagonist SB-431542 (SB) (20 μM) for 20 h. *N*=4–5. Mean±s.e.m., *P<0.05 (one-way analysis of variance with Tukey's *post hoc* tests).

Given the bidirectional regulation of *Fndc4* and *Fndc5* in the models for inflammatory disease and the recent data of Tiano *et al*.^11^ demonstrating downregulation of *Fndc5* by transforming growth factor-β (TGF-β), we set out to investigate a potential regulation of *Fndc4* by TGF-β. Interestingly, TGF-β treatment induced *Fndc4* expression levels in Caco-2 human epithelial cells (Fig. 3a). Furthermore, analysis of the activity of the *Fndc4* promoter region using a luciferase readout confirmed TGF-β-mediated activation, which was blunted by the TGF-β1R inhibitor SB-431542 (Fig. 3b). Tumour necrosis factor-α (TNFα) and lipopolysaccharide (LPS) did not induce *Fndc4* expression (Supplementary Fig. 4e–f). Thus, our data suggest that there are several potential explanations for increased *Fndc4* expression during inflammation, including TGF-β-induced expression in epithelial cells and increased infiltration of *Fndc4* expressing immune cells.

### FNDC4 reduces colitis disease progression in mice

**Figure 4:**
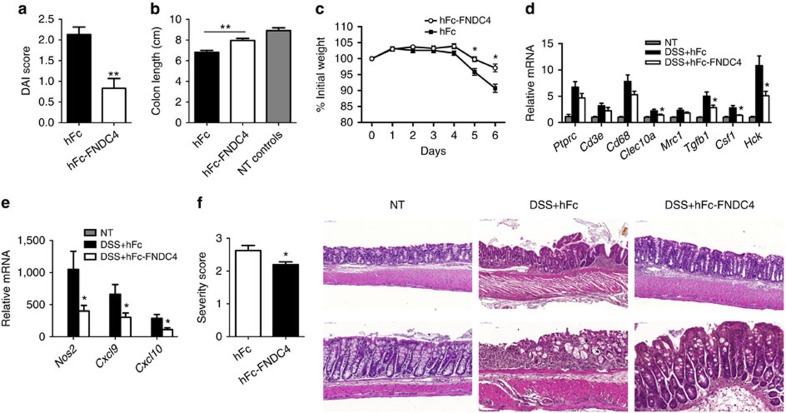
FNDC4 improves colitis in mice. DSS-induced colitis model. Mice were administered DSS from day 0 to 5 and were injected with hFc-FNDC4 or hFc control protein on days 0, 2 and 4. *N*=10 mice per group. This experiment was performed three times (of which the last experiment was blinded, blinding was performed by a colleague not involved in the study) with similar results. (**a**) DAI (disease activity index). **P<0.001, Mann–Whitney rank test. (**b**) Colon length. **P<0.001, Student's *T*-test. The non-treated (NT) control group consisted of 5 age- and gender-matched controls that did not receive DSS. (**c**) Body weight curve. *P<0.05, Student's *T*-test. (**d**–**e**) Gene expression, selected genes. Mean±s.e.m., *P<0.05 and *q*<0.10; two-sided Student's *T*-tests of hFc versus hFc-FNDC4, followed by the Benjamini-Hochberg false discovery rate (FDR) correction for multiple testing. (**f**) Histopathology. Colitis severity assessment and representative images. Scores are mean±s.e.m. *P<0.05, Mann–Whitney rank test.

We next sought to investigate the functional consequences of increased *Fndc4* expression in inflammation using a gain-of-function system in a rodent model. To this end, we produced FNDC4 (extracellular domain only) conjugated to the Fc-domain of human IgG1 (hFc) resulting in a biophysically stable protein with good kinetic properties (Supplementary Fig. 5). Importantly, all experiments were controlled with an isotype matched hFc control. Mice were injected with hFc-FNDC4 or hFc control followed by induction of colitis by addition of DSS to the drinking water for 5 days. Injections were repeated on days 2 and 4 and mice were sacrificed on day 6. Interestingly, in the FNDC4-treated group, colitis severity was significantly ameliorated, as demonstrated by a lower disease activity index (DAI)^12^ score (Fig. 4a), and a reduced shortening of the colon length, a surrogate marker for the degree of inflammation in the DSS model (Fig. 4b). Consistently, the FNDC4-injected mice lost significantly less weight, indicating a milder disease course (Fig. 4c). We also analysed gene expression in intestinal samples from these mice, where a robust induction of proinflammatory genes was observed on DSS treatment. Many of these genes were reduced in the FNDC4-treated mice compared with the control cohort (Fig. 4d,e). Especially *Cxcl9*, *Cxcl10* (Ip10), *Tgfb1* and *Csf1* were significantly reduced in the FNDC4-treated group. Furthermore, histopathology scores for colitis severity were significantly lower in the hFc-FNDC4-treated mice (Fig. 4f), supporting the beneficial effects of hFc-FNDC4 supplementation in colitis. There were no significant effects of hFc-FNDC4 treatment on body weight and colonic inflammatory gene expression in healthy control mice (Supplementary Fig. 6). In summary, therapeutic delivery of FNDC4 reduced the disease severity of colitis in mice.

### FNDC4 acts on macrophages

**Figure 5:**
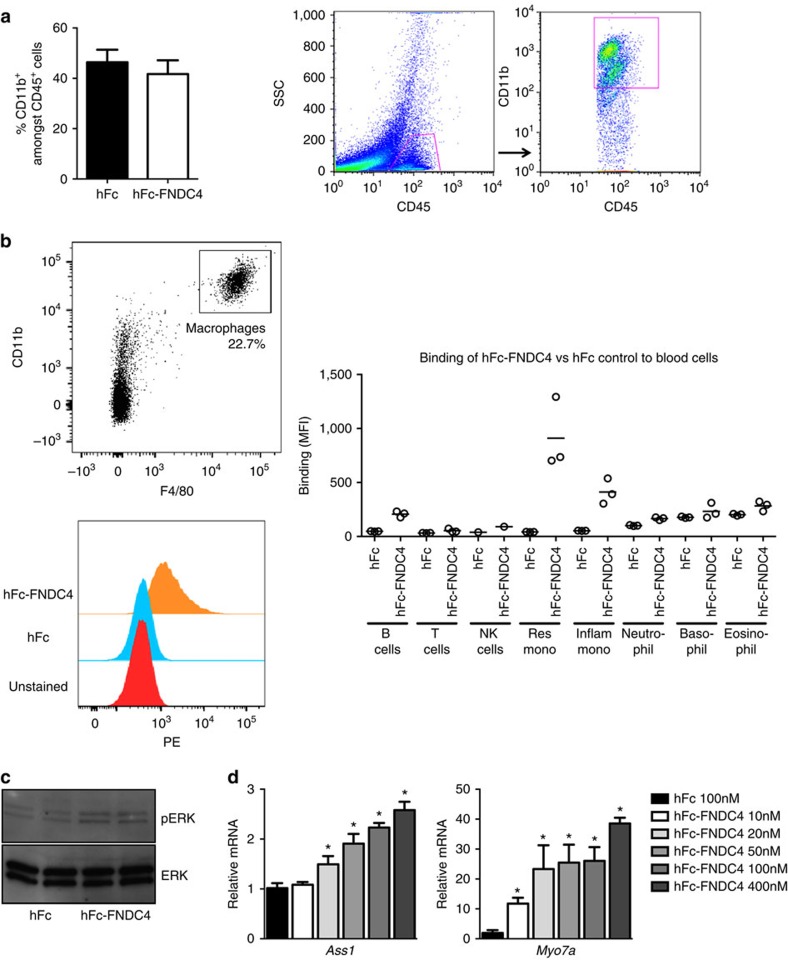
FNDC4 acts on macrophages. (**a**) Immune cell infiltration of DSS-mediated inflamed colon mostly consists of macrophages. Flow cytometry analysis of CD11b^+^-cells amongst CD45^+^ cells in the DSS colitis model. *N*=10 for the hFc control group, *N*=9 for the hFc-FNDC4 group. (**b**) FNDC4 binds to IgG Fc-gamma (IgG Fc-receptor negative) macrophages. The plots on the left show binding to peritoneal macrophages, the graph on the right shows binding to a panel of blood cells. Antibodies against CD11b and F4/80 were used to gate for macrophages. Binding of hFc-FNDC4 to cells was detected using a PE-conjugated antibody against human IgG. (**c**) ERK phosphorylation after 30 minutes of hFc-FNDC4 treatment. Total ERK served as loading control. This experiment was repeated twice with similar results. The full-size blot image is presented in Supplementary Fig. 11b. (**d**) Dose curve of hFc-FNDC4-mediated induction of *Ass1* and *Myo7a* in bone marrow macrophages treated for 24 h with different concentrations of hFc-FNDC4 (100 nM hFc as control). Data are presented as mean±s.e.m., *N*=4. *P<0.05, One-way analysis of variance followed by Dunnett's *post hoc* tests.

Subsequently, we sought to elucidate the cell type targeted by FNDC4 in colitis. To this end, we first analysed the composition of the immune cell population in the colons of DSS-treated mice using FACS and found that the majority of the CD45-positive cells were positive for CD11b (Fig. 5a). CD11b is expressed by macrophages, neutrophils and eosinophils, consistent with their reported predominance in inflammatory infiltrates in DSS-induced colitis^13^. We next used a FACS-ligand-binding assay to study the binding of hFc-FNDC4 to different immune cells. To this end, we utilized Fc-gamma receptor-deficient mice (*Fcg/2b* knockout (KO); a cross of the mouse strains described in refs 14, 15) to completely exclude any minor binding mediated by the hFc part of the hFc-FNDC4 fusion protein. Interestingly, FNDC4 displayed strong, specific binding to macrophages/monocytes, whereas neutrophils, eosinophils, T cells and NK cells did not display specific binding (Fig. 5b). Moreover, bone marrow-derived macrophages treated with FNDC4 *in vitro* displayed ERK phosphorylation (Fig. 5c) and dose-dependent target gene activation (these genes were consistently regulated on FNDC4 treatment in a range of different cell types and are used as readout for bioactivity of the hFc-FNDC4 recombinant protein; Fig. 5d) as expected^16^, demonstrating that FNDC4 induces active signalling in macrophages. As resident and recruited macrophages constitute a major cell type in acute colitis and ablation of macrophages exerts a beneficial effect on acute intestinal inflammation^17^, our results suggest that the inhibitory effects of FNDC4 on macrophages are of central importance for its therapeutic effects in the DSS model.

### FNDC4 treatment reduces macrophage phagocytosis

**Figure 6:**
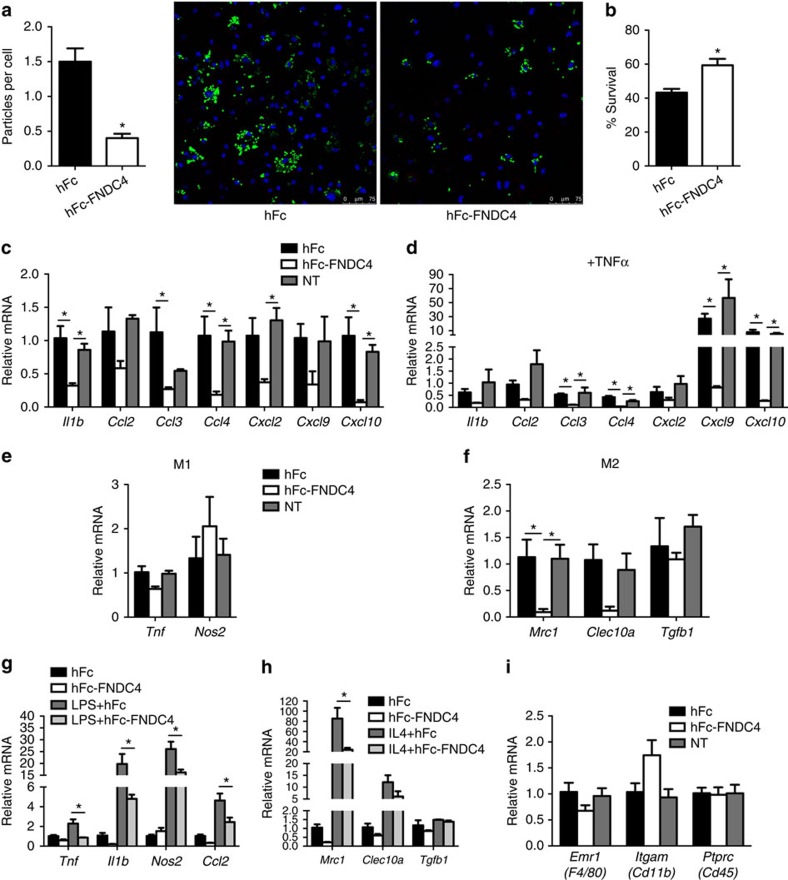
FNDC4 treatment affects key macrophage processes and downregulates proinflammatory gene expression. (**a**) Phagocytosis of fluorescently labelled zymosan A particles. Bone marrow macrophages were treated for 24 h with 100 nM hFc-FNDC4 or hFc control followed by incubation with zymosan A particles for 30 minutes. Mean±s.e.m., *N*=4. *P<0.05 (*T*-test). This experiment was performed twice with similar results. (**b**) Survival of bone marrow-derived macrophages after 48 h in starvation media, treated with 100 nM hFc-FNDC4 or hFc control. Mean±s.e.m., *N*=4. *P<0.05 (*T*-test). This effect was validated in two individual repeat experiments. (**c**) Gene expression of a selection of cytokines and chemokines on hFc-FNDC4 treatment (or hFc as carrier-control) (100 nM). *N*=6–7 (**d**) Gene expression of a selection of cytokines and chemokines on hFc-FNDC4 treatment (24 h, 100 nM) in the presence of TNFα. *N*=6–7. (**e**–**f**) Gene expression of M1 (**e**) and M2 (**f**) markers. *N*=6–7. (**g**) Gene expression of *Tnf*, *Il1b* and *Nos2* after LPS-mediated polarization. Cells were treated with 10 ng ml^−1^ LPS for 3 days before 24 h treatment with 100 nM hFc-FNDC4 or hFc control. *N*=4. (**h**) Gene expression of M2 markers *Clec10a* and *Mrc1* after IL-4-mediated polarization. Cells were treated with 10 ng ml^−1^ IL-4 for 3 days before 24 h treatment with 100 nM hFc-FNDC4 or hFc control. *N*=4. (**i**) Gene expression of the general leukocyte marker *Ptprc* (Cd45) and the general macrophage markers *Emr1* (F4/80) and *Itgam* (Cd11b). *N*=3. Mean±s.e.m. NT, non-treated. *P<0.05 and *q*<0.10 for the comparison hFc-FNDC4 versus the corresponding hFc control group; one-way analysis of variance with Tukey's *post hoc* tests, followed by the Benjamini-Hochberg FDR correction. These genes were regulated to a similar extend in the gene expression array and similar effects on gene expression were seen in multiple (>3) independent validation experiments.

A series of assays were used to assess the consequences of FNDC4 stimulation of bone marrow-derived macrophages *in vitro*. Strikingly, FNDC4 treatment strongly reduced phagocytosis of fluorescent beads (Fig. 6a). Consistent results were obtained for dead cell clearance (phagocytosis of apoptotic thymocytes; Supplementary Fig. 7a). Moreover, FNDC4-enhanced survival of macrophages after serum depletion (Fig. 6b), without indications for enhanced macrophage proliferation (Supplementary Fig. 7b). Finally, TNFα-induced production of nitric oxide (NO) production was blunted (Supplementary Fig. 7c). Thus, FNDC4 treatment resulted in a state of reduced macrophage activity, but enhanced survival. To address the transcriptional response to FNDC4 stimulation, we performed whole-genome gene expression analysis (significantly regulated genes with a fold change above 1.5 are listed in Supplementary Table 3), which confirmed the downregulation of many key pathways for macrophage activation (Supplementary Table 4). This gene ontology analysis in particular highlighted effects on phagocytosis, survival (phosphatidylinositol 3-kinase cascade), chemotaxis and cytokine secretion. Using RT-qPCR, we validated many of these findings with the reduction in the expression of key phagocytosis genes (Supplementary Fig. 8a), but also chemokines and cytokines (Fig. 6c). Importantly, this suppression of proinflammatory genes was observed both in the presence and the absence of TNFα as inflammation trigger (Fig. 6d) and was also observed in peritoneal macrophages treated with hFc-FNDC4 (Supplementary Fig. 8b). Thus, FNDC4 treatment results in an anti-inflammatory, quiescent-state macrophage as judged from activity assays and gene expression.

These effects could possibly have been attributed to ‘M2' polarization of the macrophages in culture^18^, but as shown in Fig. 6e,f, this was not the case. Interestingly, both classical M1 (*Ccl2*, *Cxcl9*, *Cxcl10*, Fig. 6c,d) and M2 markers (*Mrc1*, *Clec10a*, Fig. 6f) were downregulated by FNDC4 in the basal state. The M1 markers *Tnf* and *Nos2* (Fig. 6e) and the M2 marker *Tgfb1* (Fig. 6f) were unchanged after hFc-FNDC4 treatment in the basal state. Likewise, hFc-FNDC4 treatment resulted in downregulation of M1 and M2 markers after LPS- or interleukin-4 (IL-4)-mediated polarization towards M1 or M2 phenotypes, respectively (Fig. 6g,h), whereas identity markers such as *Ptprc* (CD45) and *Emr1* (F4/80) were unchanged (Fig. 6i). Thus, without signs of de-differentiation, these cells become largely quiescent after stimulation with FNDC4.

### FNDC4 also acts on primary human macrophages

**Figure 7:**
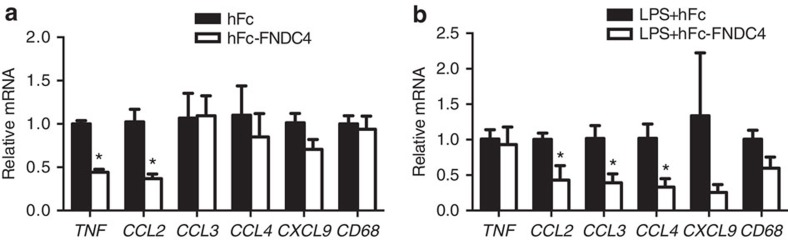
FNDC4 also acts on human macrophages. Effects of hFc**-**FNDC4 treatment on proinflammatory gene expression in the basal (3–4 individual donors) (**a**) and LPS-stimulated state (2–3 individual donors) (**b**). Macrophages were cultured in the presence of macrophage colony-stimulating factor and treated with hFc-FNDC4 or hFc control for 24 h. Subsequently, RNA was isolated using trizol, reverse transcribed and subjected to RT-qPCR analysis. Mean±s.e.m., *P<0.05 (*T*-test).

Excluding the signal peptide, the ectodomain of human FNDC4 is 100% homologous to mouse FNDC4. Thus, hFc-FNDC4 is suitable for both mouse and human applications. We therefore examined the potential effects of FNDC4 on primary human macrophages from four different donors. hFc-FNDC4 treatment significantly downregulated *TNF* and *CCL2* (MCP1) expression in both the basal (Fig. 7a) and LPS-stimulated state (Fig. 7b). Furthermore, under LPS-stimulated conditions, hFc-FNDC4 treatment also reduced the expression of the proinflammatory chemokines *CCL3*, *CCL4* and *CXCL9* (Fig. 7b).

### FNDC4 KO mice exposed to DSS show increased colitis severity

**Figure 8:**
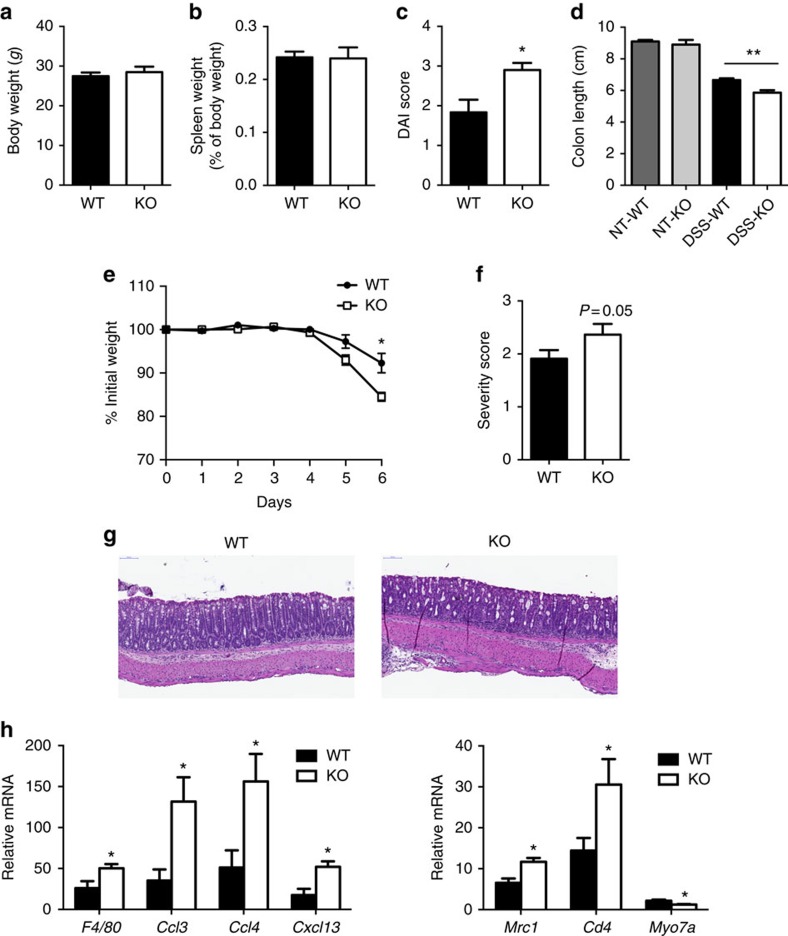
FNDC4 knockout mice show increased inflammation and severity of DSS-induced colitis. (**a**) Body weights of 12 week old mice, *N*=4. (**b**) Spleen weight, standardized to body weight at 16 weeks of age, *N*=4. (**c**) DAI (disease activity index). *N*=5 mice per group. *P<0.05, Mann–Whitney rank test. KO, *Fndc4* knockout mice, WT, wild-type mice. (**d**) Colon length. **P<0.01, analysis of variance with Tukey's *post hoc* test. (**e**) Body weight curve. *P<0.05 and *q*<0.05, Student's T-test followed by the Benjamini-Hochberg false discovery rate correction. (**f**-**g**) Histopathology. Colitis severity assessment. *N*=11 mice per group, *P<0.05, Mann–Whitney rank test. (**h**) Gene expression, selected genes. *P<0.05; two-sided Student's *T*-tests, *N*=5 mice per group. Disease severity was assessed blinded and the experiment was repeated with similar results. Mean±s.e.m.

To evaluate whether FNDC4 is not only sufficient, but also required for immune dampening in colitis, we investigated DSS-induced colitis development and severity in *Fndc4* KO mice. *Fndc4* KO mice were generated by crossing mice with floxed *Fndc4* alleles with CMV-Cre mice. Heterozygote breeding pairs gave normal Mendelian distribution and average litter sizes (average litter size of seven mice). Furthermore, the KO mice had no detectable developmental defects. Body weight was similar between *Fndc4* KO and littermate controls (Fig. 8a). Furthermore, spleen appearance (histology) and weight (Fig. 8b) was unaffected. Moreover, baseline gene expression profiles of a panel of immune cell and inflammation markers were similar in spleens of KO and WT mice (Supplementary Fig. 9).

Induction of colitis resulted in increased disease severity in the *Fndc4* KO mice, as shown by increased disease severity index (Fig. 8c), reduced colon length (Fig. 8d), increased body weight loss (Fig. 8e), increased histopathology scores (Fig. 8f,g) and increased proinflammatory gene expression (Fig. 8h).

### FNDC4 acts partly via STAT3

**Figure 9:**
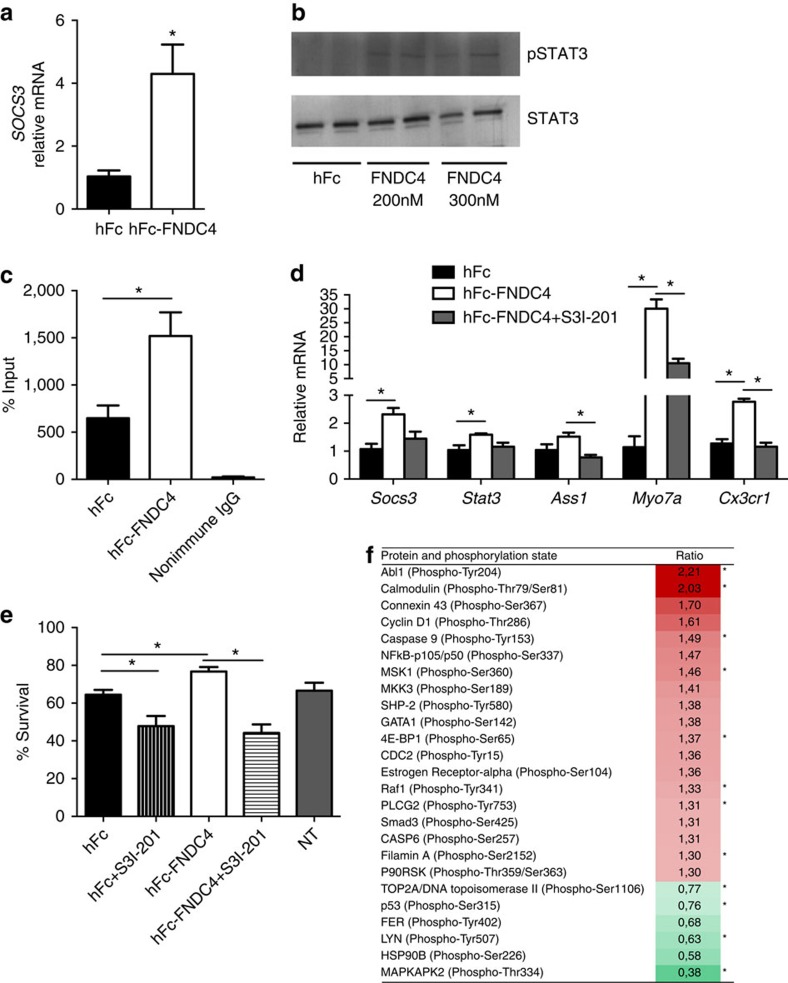
FNDC4 signals partly via STAT3. (**a**) *Socs3* gene expression after 4 h incubation with 100 nM hFc-FNDC4 or hFc control. Mean±s.e.m., *N*=3. *P<0.05 (*T*-test). (**b**) Phosphorylation of STAT3. Bone marrow-derived macrophages were starved overnight followed by 30 minutes treatment with 200 nM or 300 nM hFc-FNDC4, 200 nM hFc served as control. The full-size, uncropped blot image is presented in Supplementary Fig. 11c. Similar effects were observed in two repeated experiments. (**c**) STAT3 DNA binding to the *Socs3* promoter region. Macrophages were treated with hFc-FNDC4 or hFc for 6 h. Data presented as percentage of input. Mean±s.e.m., *N*=4. *P<0.05 (*T*-test). (**d**) STAT3 inhibition reverses FNDC4-mediated gene regulation. Bone marrow-derived macrophages were pretreated with 50 μM S3I-201 STAT3 inhibitor or DMSO as control for 45 min and subsequently treated with 100 nM hFc-FNDC4 or hFc control for 6 h in the presence or absence of S3I-201. Mean±s.e.m., *P<0.05 and *q*<0.10, One-way analysis of variance followed by Tukey's *post hoc* tests and Benjamini-Hochberg false discovery rate-adjustment, *N*=4. (**e**) STAT3 inhibition reverses FNDC4-mediated improvements of macrophage survival. Bone marrow macrophages were pretreated with 50 μM S3I-201 STAT3 inhibitor or DMSO as control for 45 min and subsequently treated with 100 nM hFc-FNDC4 or hFc control for 48 h in starvation media in the presence or absence of S3I-201. NT, non-treated. Mean±s.e.m., *P<0.05, one-way analysis of variance followed by Tukey's *post hoc* tests, *N*=8 per condition. (**f**) Proteins showing a >1.3-fold increase or decrease in phosphorylation status in primary bone marrow macrophages on 30-min treatment with hFc-FNDC4 or hFc control. The fold change was calculated as the signal ratio of the paired antibodies for each pair of site-specific antibody and phospho site-specific antibody. Average ratios of two duplicates per condition. * Indicate that the fold induction in phosphorylation status is due to a reduction in binding of the total protein (conformational change affecting the accessibility of the epitopes; the array is performed using non-denatured protein).

We then proceeded to identify signalling pathways involved in the effects of FNDC4 on macrophages. STAT3 is an important regulator of inflammatory processes^19^,^20^ and was shown to be activated on irisin treatment of neuronal cells^21^. Furthermore, gene expression analysis of bone marrow-derived macrophages treated with hFc-FNDC4 revealed overrepresentation of the GO classes associated with peptidyl-tyrosine phosphorylation and JAK-STAT signalling (Supplementary Table 3). Therefore, we examined whether FNDC4 affects STAT3 activation. The STAT3 target gene *Socs3* was increased fourfold after 4 h of treatment with 100 nM hFc-FNDC4 protein (Fig. 9a) and showed a dose-response pattern (Supplementary Fig. 10a). Furthermore, phosphorylation of STAT3 (Fig. 9b; but not STAT6, Supplementary Fig. 10b) was induced after 30 min of FNDC4 treatment. FNDC4-mediated STAT3 activation was further supported by STAT3 DNA binding to the *Socs3* promoter region (chromatin immunoprecipitation (ChIP) assay; Fig. 9c). Importantly, inhibition of STAT3 resulted in reversal of gene regulation of several FNDC4 regulated genes (Fig. 9d) and reversal of FNDC4-mediated improvements in macrophage survival (Fig. 9e), indicating that STAT3 is at least partly responsible for FNDC4 downstream signalling.

To identify other pathways involved in the actions of FNDC4 on macrophages, we performed a phospho-antibody array containing 1,318 site-specific antibodies from over 30 signalling pathways. The phosphorylation sites with a foldchange of at least 1.3 are listed in Fig. 9f. Phosphorylation of FER (pTyr402) and HSP90B (pSer226) was reduced by FNDC4 treatment, while LYN (pTyr507) and MAPKAPK2 (pThr334) showed the strongest reduction in phosphorylation ratio due to increased total protein binding, indicating possible conformational changes in the protein. FNDC4-induced phosphorylation was strongest for Connexin 43 (pSer367), Cyclin D1 (Thr286) and NFκB p105/p50 (pSer337). Abl1 (pTyr204), Calmodulin (pThr79/pSer81) and Caspase 9 (pTyr153) showed increased phosphorylation ratios due to reduced total protein binding.

In summary, we have characterized a novel secreted factor—FNDC4—which signals to macrophages to downregulate inflammation. FNDC4 supplementation reduced disease severity in a mouse model for colitis, indicating therapeutic potential.

## Discussion

Here we characterized the immunomodulating properties of FNDC4, of which to our knowledge no functional reports exist to date. FNDC4 treatment of mice with DSS-induced colitis resulted in reduced inflammation and clinical improvement, suggesting therapeutic potential. We show that FNDC4 binds to and acts on macrophages to downregulate proinflammatory gene expression. Furthermore, key macrophage functions including phagocytosis were strongly affected by FNDC4 treatment, and these effects were partly mediated by STAT3 activation. Importantly, *Fndc4* KO mice showed increased colitis severity, suggestive of a requirement for FNDC4 to dampen the immunological response in colitis.

*Fndc4* gene expression was upregulated with inflammation in human IBD and in inflamed tissue in several mouse models for inflammatory diseases including DSS colitis and nephritis, indicating that FNDC4 may play a physiological role in the regulation of inflammation, potentially counteracting (consequences of) inflammation. Furthermore, we showed that TGF-β1 upregulates *Fndc4* expression, which might explain the induction of *Fndc4* expression with inflammation, in addition to potentially increased infiltration of *Fndc4*-expressing immune cells. *Fndc4* is highly expressed in many tissues including liver, brain, heart, adipose tissue and spleen, while it is barely expressed in macrophages. It is thus likely that non-macrophage cells signal to macrophages in a paracrine- or endocrine fashion. The cellular source of FNDC4 protein in inflammatory disease will be an important focus for subsequent studies. The lack of a robust and valid antibody for detection of endogenous FNDC4 levels prevented us from including endogenous protein expression data in the current report. Yet, *in situ* RNA hybridization experiments revealed FNDC4 expression in colonic epithelium as well as a subset of immune cells in lymphoid aggregates. Immune cell expression profiles further support expression of *Fndc4* in several immune cell subtypes, including neutrophils, natural killer cells, dendritic cells and T-cell subtypes. Also monocytes showed *Fndc4* expression. However, as for many other peptide hormones, the cellular source for FNDC4 during inflammation is not fully clarified. This will need to be addressed in future studies.

FNDC4 displayed strong binding to macrophages and dampened macrophage inflammatory gene expression. Macrophages were also the most prominent inflammatory cell type in the colitis model used. Moreover, inflammation in colitis is largely mediated by macrophages, as supported by reports demonstrating beneficial effects of targeting macrophage inflammation in mouse models for colitis^22^,^23^,^24^. Altogether, this suggests that FNDC4 exerts its anti-inflammatory effects via macrophages, although we cannot completely rule out contributing effects on other (nonimmune) cell types.

Administering FNDC4 to mice with DSS-induced colitis resulted in downregulation of expression of the proinflammatory chemokines *Cxcl9* and *Cxcl10* (Ip10), consistent with the *in vitro* data in macrophages. In accordance, beneficial effects of counteracting chemokine signalling in DSS-induced colitis have been shown in several reports. Tokuyama *et al*.^25^ showed attenuation of DSS-induced colitis on treatment with a broad spectrum chemokine receptor antagonist. Furthermore, Chami *et al*.^26^ showed that blockage of *Cxcl9* and *Cxcl10* signalling by loss of function of its receptor, CXCR3, resulted in attenuation of colitis symptoms. Thus, reduced secretion of relevant chemokines by macrophages represents a relevant therapeutic response to FNDC4 treatment.

Interestingly, we observed a strong downregulation of *in vitro* phagocytosis activity of bone marrow macrophages in culture after treatment with FNDC4. It is thus likely that therapeutic delivery of FNDC4 partially blocks phagocytosis also *in vivo*, which would be consistent with previous reports where blockage of phagocytosis function attenuated colitis in mice^27^.

Another interesting finding is the downregulation of both M1 and M2 markers on FNDC4 treatment of macrophages in culture. Previously, macrophage activation has been characterized as polarized to either M1 or M2 phenotypes. Yet, recent developments in the field indicate that this classification is too simplified and that there is a spectrum of macrophage functional states spanning between M1 and M2 definitions^28^,^29^. Our data indicates that FNDC4 action on macrophages results in a quiescent-state macrophage with increased survival but downregulated expression of several M1 and M2 markers. This phenotype did not match any of the previously described M2 subpopulations neither the recently proposed M3 ‘repair' phenotype^30^. Further research will be required to further characterize this macrophage state.

The effects of FNDC4 on macrophages were partly mediated by STAT3, which is a transcription factor involved in regulating inflammatory processes and cell survival^31^,^32^. The role of STAT3 in inflammation, however, is complex and cell type specific. For example, targeted deletion of STAT3 in myeloid cells in mice results in spontaneous chronic enterocolitis^33^, while STAT3 deletion in T cells is associated with reduced colitis symptoms^34^,^35^. Likewise, risk allele analysis has indicated that both increased and reduced STAT3 activity is associated with increased risk for IBD^36^,^37^. Since FNDC4 mainly targets macrophages, STAT3 activation may have contributed to the attenuation of colitis symptoms in the FNDC4-treated mice. Yet, FNDC4 effects were only partly STAT3 dependent. Other transcription factors and downstream signalling pathways are likely to contribute to the anti-inflammatory actions of FNDC4. In addition to the STAT3 target gene *Socs3*, *Socs2* was strongly upregulated in macrophages treated with FNDC4, indicating possible involvement of STAT5 signalling.

Extensive phosphoprotein profiling revealed potential involvement of heat shock protein 90 (HSP90), ABL1, NFκB p50 and oestrogen receptor-α in the effects of FNDC4 on macrophages and further supported a role for the ERK/p38 pathway (MKK3, MSK1, MAPKAPK2, SHP-2) downstream of FNDC4. Interestingly, the BCR–ABL complex was shown to suppress HSP90 phosphorylation at Ser226, resulting in a reduction of apoptosome function and thus improved cell survival^38^. Although HSP90 inhibition was shown to dampen inflammation *in vitro*^39^ and *in vivo*^40^,^41^, the role of Ser226 phosphorylation of HSP90 in inflammation in macrophages is unclear. We observed an upregulation of Ser337 phosphorylation of NFκB p105/p50. NFκB p50 is constitutively phosphorylated by PKAc at Ser337 (ref. 42) and in contrast to p50/p65 heterodimers that are mostly involved in proinflammatory processes, p50 homodimers are mostly bound to DNA in resting cells where they maintain negative regulation of NFκB gene expression^42^. Finally, it has been well established that oestrogen receptors have an important role in modulating anti-inflammatory processes^43^.

In conclusion, we have characterized a novel secreted factor, FNDC4, targeting macrophages. *Fndc4* is upregulated with inflammation in human IBD and in several mouse models for inflammatory diseases. FNDC4 treatment of macrophages improved cell survival, downregulated proinflammatory gene expression and strongly reduced phagocytosis. FNDC4 supplementation *in vivo* attenuated DSS-induced colitis, indicating that FNDC4 has therapeutic potential. The use of a human Fc-bound recombinant FNDC4 protein ensures prolonged *in vivo* stability of the protein and may have the propensity for direct use in humans.

## Methods

### Gene expression analysis

RNA-extraction was performed by trizol extraction followed by cleanup using the RNeasy system (Qiagen, Venlo, The Netherlands). Subsequently, complementary DNA synthesis was performed using the High Capacity Reverse Transcription Kit from Applied Biosystems (Life Technologies, Bleiswijk, The Netherlands) following the manufacturer's instructions. RT-qPCR was performed using the iTaq Universal SYBR Green Supermix (Bio-Rad, Solna, Sweden) on a ViiA7 Real-Time PCR System (Life Technologies) and quantified using the ΔΔCt method where *TBP* was used as housekeeping gene. In the DSS-induced colitis model, RNA was purified with lithium chloride as described previously^44^, since DSS inhibits reverse transcriptases and polymerases^44^. Primer sequences are listed in Supplementary Table 1. Affymetrix global gene expression analysis (MoGene-1_1-st-v1 arrays) was performed in the BEA core facility of Karolinska Institutet. Three samples per group were used and genes displaying probe intensity >100 and *P* values <0.05 using Student's *T*-tests were selected for downstream analysis. Furthermore, *q* values were calculated (corrected for multiple testing). Microarray data have been deposited in NCBI's Gene Expression Omnibus and are accessible through GEO series accession number GSE76172.

### Animal experiments

All animal procedures were in compliance with protocols approved by local government authorities (Institutional Animal Care and Use Committee of Karolinska Institutet, Stockholm, Sweden).

Male C57Bl/6N mice aged 10–14 weeks were housed under specific-pathogen-free (SPF) conditions in groups of 3 to 10 in a 12-h–12-h light–dark cycle (with lights on at 06:00 hours) and fed standard chow diet.

Colitis was induced by administration of 3% DSS (MW 40 kDa, #DB001, TdB Consultancy, Uppsala, Sweden) with the drinking water, provided *ad libitum* for 5 days. The non-treated control group received tap water only. 2.7 mg kg^−1^ body weight hFc- FNDC4 or hFc control protein (amount matched for molecular weight) was injected on days 0, 2 and 4. Body weight was measured daily. The disease activity index was based on Cooper *et al*.^12^ It is a combined score of stool consistency, intestinal bleeding and weight loss.

Glomerulonephritis was induced by the administration of antiserum raised against the glomerular basement membrane (Probetex, San Antonio, Texas, USA). 200 μl of serum was injected intraperitoneally. Mice were sacrificed 5 days post injection.

Transient ischaemia reperfusion injury of the kidney was performed as described previously^45^.

*Fndc4* KO mice were generated by crossing mice harbouring *lox*P sites flanking exons 2–5 of the *Fndc4* locus with CMV-Cre-recombinase expressing mice (B6.C-Tg(CMV-cre)1Cgn/J, Jackson Laboratories), resulting in *Fndc4* KO mice on a mixed C57Bl/6NJ background. *Fndc4* KO mice were born with the expected Mendelian ratio, had no detectable developmental defects, and were fertile with a normal litter size.

### Human IBD cohort

During routine endoscopy in 52 adult patients investigated for a known IBD diagnosis or in the work-up for suspected gastrointestinal disorders (Supplementary Table 2), colorectal and ileal mucosal biopsy specimens were collected. Nineteen patients not afflicted with IBD and without intestinal inflammation or pathological findings were included as non-inflamed non-IBD controls. Study biopsies were collected in parallel to, and from the same locations as biopsies for histopathologic assessment. Each biopsy was categorized as ‘inflamed' or ‘non-inflamed' based on a composite evaluation of macroscopic findings assessed by one experienced endoscopist (S.A.) and the routine histopathologic assessment.

Biopsy specimens for RNA purification were immersed in RNA*later* RNA stabilization reagent (Qiagen, Hilden, DE) and stored at 4 °C overnight, and thereafter at −20 °C awaiting RNA purification.

This part of the study was approved by the Regional Ethical Review Board in Linköping, Sweden (Dnr 2011/201-31). Written informed consent was obtained from all participants.

### Cell culture

Bone marrow-derived macrophages were generated from C57Bl/6N mice as previously described^46^. Macrophages were cultured in DMEM supplemented with 10% FCS, 1% PenStrep (all from Gibco/Life Technologies, Bleiswijk, The Netherlands) and 20% conditioned media collected from L929 cells. Before experiments, cells were starved in 1% FCS DMEM for 24 h. Further treatments were also performed in starvation media.

For STAT3 inhibition, cells were incubated with 50 μM S3I-201 (Santa Cruz Biotechnology, Dallas, Texas, USA).

Peripheral blood mononuclear cells were isolated from buffy-coated blood obtained from healthy blood donors using Ficoll-Hypaque gradient centrifugation (BD Diagnostics, Franklin Lakes, NJ), followed by monocyte isolation using the EasySep Human monocyte enrichment kit without CD16 depletion (StemCell Technologies, Vancouver, Canada), according to manufacturers protocols. 0.5–1.0 × 10^6^ monocytes per well were plated in six-well plates with complete RPMI media supplemented with 50 ng ml^−1^ colony-stimulating factor-1 (Biolegend, San Diego, CA) for 8 days to generate macrophages.

### *Fndc4* promoter luciferase assay

McArdle cells were transfected with a pGl3 reporter vector (Promega) containing the mouse *Fndc4* upstream promoter region using Lipofectamine 2000 (Life Technologies) as transfection reagent. After 8 h, cells were treated with TGF-β1 (20 ng ml^−1^; Peprotech, Stockholm, Sweden) and/or SB-431542 (20 μM; Merck Millipore) for 20 h. The luciferase assay (Dual-Glo, Promega) was performed according to the manufactures instructions. Values are reported relative to the non-treated control group.

### Production of Fc-fusion proteins

His-Fc-FNDC4 fusion protein was expressed using pEFIRES expression vector^47^. The DNA fragment coding signal peptide (SP) from Fndc5 fused to 6His-FC was synthesized by GeneScript, Inc., USA, and cloned into the pEFIRES modified multiple cloning site using *Nhe*I and *Not*I restriction sites. The extracellular domain of the *Fndc4* gene was PCR-amplified using mouse clone MR223815 (Origene, Rockville, MD, USA) as a template and set of primers: forward: 5′-GAGAGCGGCCGCTCGACCTCCCTCTCCTGTG-3′; reverse: 5′-GAGAGAATTCATTCCCCTGTCTGCAATGGC-3′. The amplified *Fndc4* fragment was cloned into an SP-6His-Fc-pEFIRES vector using *Not*I and *Eco*RI restriction sites to produce an SP-6His-Fc-Fndc4-pEFIRES expression construct. These constructs were transfected to CHO-S cells using Lipofectamine 2000 (Life Technologies) and stably producing cell lines were selected using puromycin (Sigma-Aldrich, St Louis, MO, USA) as selection agent. For protein production stable CHO-S suspension cultures were grown in CD OptiCHO medium (Life Technologies) supplemented with Ala-Glu (Sigma-Aldrich). Culture supernatants were loaded onto HisTrap Excel columns (GE Healthcare, Stockholm, Sweden) in 5 mM imidazole containing buffer, washed with 20 mM imidazole and eluted with 400 mM imidazole. Eluted protein was dialyzed against 3 × PBS buffer. Purity of the produced recombinant protein was about 95% (based on Coomassie staining). The protein concentration was measured using the BCA Protein Assay Kit (Pierce Biotechnology, Inc./Thermo Fischer Scientific, Rockford, IL, USA). Protein purity was assessed by SDS–PAGE and Coomassie staining using PageBlue Protein Staining Solution (Thermo Fisher Scientific). Kinetics of the recombinant protein were assessed by analysis of plasma protein content and stability. hFc-FNDC4 or hFc control protein was injected into 9- to 10-week-old C57Bl/6 mice (single injection of 3 mg kg^−1^ body weight). Plasma was collected after 24 h and 5 days and boiled in reducing buffer for 7 min. 0.2 μl of plasma per sample was blotted against hFc (IgG). Recombinant hFc and hFc-FNDC4 was spiked into plasma and treated in the same way as the experimental samples to serve as positive controls.

### Analysis of FNDC4 secretion from HEK293 cells

The FNDC4 N-terminal FLAG, C-terminal Myc construct was created by modifying a C-terminally tagged FNDC4 mouse complementary DNA clone purchased from Origene (MC210221). The full-length, C-terminally modified FNDC4 was first cloned into the pENTR-1a dual-selection vector (Invitrogen). The N-terminal FLAG tag was subsequently inserted between FNDC4 S49 and P50 via site-directed mutagenesis. The C-terminal FLAG tag was removed by introducing a stop codon directly after the C-terminal Myc, also by site-directed mutagenesis, resulting in an FNDC4 construct with an N-terminal FLAG and a C-terminal Myc. The site-directed mutagenesis was performed using the QuikChange XL Site-Directed Mutagenisis Kit (Agilent). The resulting construct was then recombined with the pT-REx-DEST30 gateway vector from Invitrogen for transient expression in mammalian cell lines.

HEK293 cells were cultured in DMEM-GlutaMAX supplemented with 10% fetal bovine serum and 1% PenStrep (Invitrogen). Transient transfections were performed using Lipofectamine 2000 (Invitrogen) in accordance with the manufacturer's instructions. Transfected cells were cultured for 24 h, washed and subsequently switched to serum-free FreeStyle 293 Expression Media (Gibco). The conditioned FreeStyle media and cell lysates were harvested after 24 h, and media was filtered and concentrated 20 ×, after which both cell lysates and media were probed using western blot.

### FNDC4 binding to immune cells

Blood was collected from FcgR-deficient mice (Fcg/2b DKO) on C57BL/6 background and red blood cells were lysed with two rounds of RBC lysis buffer (BioLegend, San Diego, CA, USA). Peritoneal macrophages were collected from the same mice by lavage from the peritoneal cavity using 5 ml ice cold PBS. Cells were dissolved in FACS buffer (PBS+3% FBS) containing hFc-FNDC4 or hFc control (0.5 μM) and incubated 1 h in room temperature, washed, followed by 1 h incubation with an anti-human IgG secondary antibody conjugated to PE (Life Technologies, H10104, 1:200) and washing steps. Subsequently, cells were labelled with antibodies to define cell populations (CD11b, B220, TCRb, GR-1, CD123, NK1.1 and F4/80). After washings, cells were collected on a BD LSRII FACS and analysed by FlowJo software. Dead cells were excluded on the basis of labelling with 7-AAD and duplicates excluded by a FSC-H FSC-A gate. Quantification was performed by defining the mean fluorescence intensity of PE on gated cell populations.

### RNA *in situ* hybridization

*In situ* RNA hybridization for *Fndc4* mRNA was performed on formaldehyde-fixed, paraffin-embedded colonic tissue using RNAscope technology (Advanced Cell Diagnostics, Hayward, CA, USA) with custom-made probes for mouse *Fndc4*, following the manufacturer‘s instructions (RNAscope 2.0 HD red detection kit). Probes targeting *Ubc* and *Dabp* were used as positive and negative control probes, respectively. Slides were counterstained with hematoxylin.

### Histopathology

Renal tissue was stained with periodic acid Schiff's stain for histological analysis. To examine the glomerular injury, the glomerular damage index (0: none, 1: slight, 2: moderate, 3: severe) was determined by assessing glomerular cellularity, mesangiolysis, glomerular expansion and crescent formation. Crescent formation was defined as the presence of three or more layers of cells in Bowman's space.

Colon pathology was assessed as described previously^48^. Briefly, severity of lesion was graded as follows: 0=normal, 1=mild, 2=moderate, 3=severe. Mild lesions were small, focal or widely separated multifocal areas of inflammation limited to the lamina propria. Moderate lesions were either multifocal or represented by locally extensive areas of inflammation extending into the submucosa. Severe lesions were ulcers covering large areas (>20 crypts) of the mucosa.

### Western blotting

Western blots were performed with cell lysates in RIPA-lysis buffer mixed 1:1 with Laemmli sample buffer (Bio-Rad, Mississauga, Ontario, Canada) and processed for standard SDS–PAGE and western blotting. Protein concentration was determined and equal amounts of protein were loaded per lane. The nitrocellulose membranes were incubated with antibodies directed against phosphoERK (1:2,000; Thr202, Tyr204; #9101; Cell Signaling, Leiden, The Netherlands), ERK (1:2,000; #4695; Cell Signaling), phosphoSTAT3 (Tyr705) (1:1,000; #9145; Cell Signaling), STAT3 (1:500; #sc-482; Santa Cruz Biotechnology, Dallas, Texas, USA) and phosphoSTAT6 (1:1,000; #ab54461; Abcam, Cambridge, UK). Detection of phosphorylation versus total protein levels (pERK/ERK, pSTAT3/STAT3) was performed on separate gels (no stripping). Antibody binding was revealed by incubation with donkey anti-rabbit horseradish peroxidase-linked IgG (1:10,000) (#97085; Abcam) and the SuperSignal West Femto immunoblotting detection system (Thermo Scientific). Chemiluminescence was detected by autoradiography using Amersham Hyperfilm ECL (GE Healthcare, Uppsala, Sweden). Images have been cropped for presentation. Full-size images are presented in Supplementary Fig. 11.

### Phagocytosis assay

Alexa Fluor 488 conjugated zymosan A particles (Life Technologies, Stockholm, Sweden) were diluted in starvation media to obtain a concentration of 2E6 particles per ml. Bone marrow macrophages were incubated with the zymosan particles for 30 min followed by five washes in PBS to wash away non-phagocytosed particles. On fixation in 4% formaldehyde, nuclei were stained with DAPI and cells were imaged using a Leica TCS SP8 confocal laser-scanning microscope (Leica Microsystems, Kista, Sweden).

### Apoptotic cell clearance assay

Dead cell clearance was performed as described earlier^49^. Briefly, bone marrow macrophages were treated with hFc-FNDC4 or hFc control for 24 h before incubation with CellTracker Orange CMTMR (Life Technologies)-labelled apoptotic thymocytes (dexamethasone-induced). Phagocytosis of apoptotic thymocytes was assessed after 1 h.

### NO assay

NO production was assessed in cell culture media using the nitrate/nitrite calorimetric assay from Cayman Chemicals (Ann Arbour, Michigan, USA).

### Flow cytometry

After separation from the small intestine the colon was cut longitudinally and one half was collected in ice-cold PBS for flow cytometry analysis. The colon was further cut into 1 mm pieces. Subsequently, colon pieces were incubated in magnesium and calcium-free HBSS (Life Technologies) containing 1 mM dithiothreitol and 5 mM EDTA at 37 °C for 20 min with shaking, after which the EDTA- and DTT-containing buffer was refreshed followed by another 20 min. incubation at 37 °C. Then, colon pieces were treated with 20 μ ml^−1^ collagenase D and 2% DNAse I (Sigma-Aldrich) for 45 min at 37 °C with shaking. Single cells were obtained by filtering through a 100 μm cell strainer. Samples were centrifuged for 5 min. at 600*g* to pellet cells. Subsequently, cell pellets were resuspended in FACS buffer (PBS supplemented with 3% FCS and 10 mM EDTA) containing Fc block, 1:200 (#553141; BD Biosciences). Cells were incubated for 15 min. at room temperature, followed by a wash in FACS buffer. Subsequently, cells were incubated with an antibody cocktail containing CD45.2-FITC (#109806, BioLegend) and CD11b-PE (#557397, BD Biosciences) at 1:200 for 30 min on ice. Cells were washed once and resuspended in FACS buffer containing 1:30 diluted 7-AAD staining solution (BioLegend) to determine cell viability. Samples were filtered through 50 μm nylon gauze before analysis on a FACSCanto II with FACSDiva software (BD Biosciences). Unstained and single-stained control samples were included in the experiments. Acquired data were gated and analysed with FlowJo software (TreeStar, Inc., Ashland, OR, USA).

### Phosphoprotein profiling

Cell lysates were obtained from primary bone marrow macrophages treated for 30 min with hFc-FNDC4 or hFc control protein (*N*=2 per condition). The Phospho Explorer antibody microarray, which was designed and manufactured by Full Moon Biosystems, Inc. (Sunnyvale, CA), contains 1,318 antibodies. Each of the antibodies has two replicates that are printed on a coated glass microscope slide, along with multiple positive and negative controls. The antibody array experiment was performed by Full Moon Biosystems, according to their established protocols^50^.

The slides were scanned on an Axon GenePix array scanner, and the images were analysed with GenePix Pro 6.0 (Molecular Devices, Sunnyvale, CA). The fluorescence signal (*I*) of each antibody was obtained from the fluorescence intensity of this antibody spot after subtraction of the blank signal (spot in the absence of antibody). For each phospho-specific antibody, a phosphorylation signal ratio was calculated.

### ChIP assay for analysis of STAT3 DNA binding

Bone marrow-derived macrophages were starved overnight followed by 6 h treatment with 200 nM hFc-FNDC4 or hFc control. Cells were crosslinked for 10 min with 1% formaldehyde, followed by quenching for 5 min with 12.5 mM glycine. Cells were further processed for ChIP analysis using the Magna ChIP A/G chromatin immunoprecipitation kit (Millipore,Solna, Sweden), following the manufacturer's instructions. Briefly, after cell and nucleus lysis, chromatin was sheared 10 times (20 s on and 30 s off) with a Bioruptor sonicator (Diagenode, Seraing, Belgium) to yield fragment sizes of 100–500 base pairs. After 10% of the sample was saved as 'input control', sheared chromatin was immunoprecipitated overnight with antibody to STAT3 (#sc-482; Santa Cruz Biotechnology) and normal rabbit IgG (Sigma-Aldrich) as negative control, followed by 1 h of incubation at 4 °C with Protein A/G magnetic beads. After washing and reverse crosslinking, DNA was purified and analysed using iTaq Universal SYBR green supermix (Life Technologies, Stockholm, Sweden) on a ViiA7 Real-Time PCR System (Life Technologies). Primers sequences for RT-qPCR analysis of binding to *Socs3* were: 5′-CCCCCAACTTCTCATTCACA-3′ (forward) and 5′-TACATGAGGACCTCGGAGTG-3′ (reverse)^51^. Promoter occupancy was calculated as percentage of input DNA.

### Statistics

Differences between groups were evaluated using two-tailed unpaired or paired (IBD cohort) Student's *T*-tests for single comparisons or one-way analysis of variances in combination with Tukey's *post hoc* tests for multiple comparisons when data was normally distributed, and Mann–Whitney rank tests for non-normally distributed data (scores). The Benjamini–Hochberg procedure was used to correct for multiple testing for experiments where gene expression of a large panel of genes was shown. In these cases, the level of significance was set at false discovery rate (*q*)=0.10. Correlations were evaluated using Pearson's correlation coefficients. IBM SPSS Statistics version 22 and GraphPad Prism version 6 were used for statistical analyses. *P*<0.05 was considered statistically significant.

## Additional information

**How to cite this article:** Bosma, M. *et al*. FNDC4 acts as an anti-inflammatory factor on macrophages and improves colitis in mice. *Nat. Commun.* 7:11314 doi: 10.1038/ncomms11314 (2016).

## Supplementary Material

Supplementary Information Supplementary Figures 1-11 and Supplementary Tables 1-4
