# Supplementary material for: FNDC4 acts as an anti-inflammatory factor on macrophages and improves colitis in mice
Source: Nat Commun. 2016 Apr 12;7:11314. doi: 10.1038/ncomms11314 (PMC4832079; doi:10.1038/ncomms11314)
Supplement: Supplementary Information — Supplementary Figures 1-11 and Supplementary Tables 1-4 [file ncomms11314-s1.pdf]

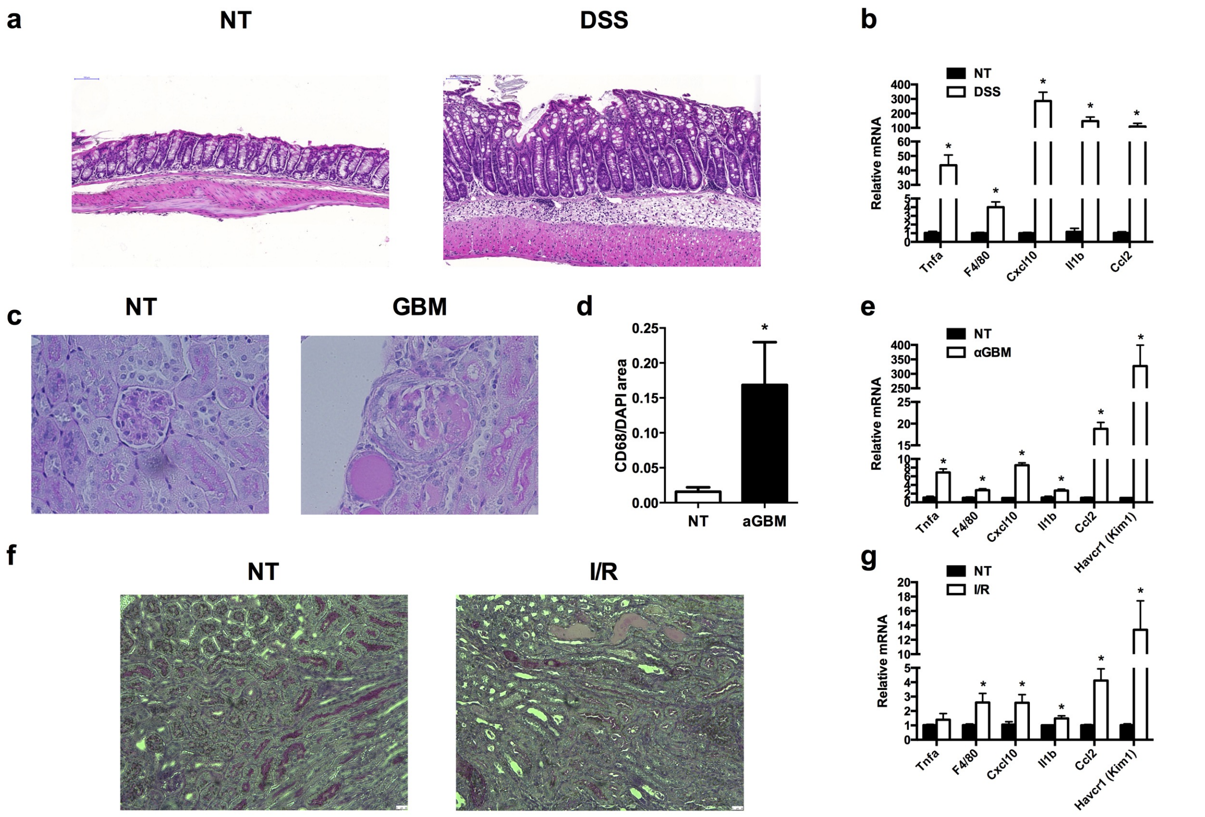

Supplementary Figure 1. Histology and gene expression of inflammatory markers in the three models for inflammatory disease. (a) Histopathology DSS model. Representative pictures (H&E staining). (b) Gene expression of inflammatory genes in the DSS model. (c) Histopathology αGBM-induced glomerulonephritis. Representative pictures (PAS staining). (d) CD68 quantification as a measure for macrophage infiltration in the αGBM model. CD68 was quantified as the CD68-positive area relative to the DAPI (nuclear) area. (e) Gene expression of inflammatory genes in the αGBM-induced glomerulonephritis model. (f) Histopathology of kidneys 3 days after ischemia-reperfusion (I/R). Representative images (H&E staining). (g) Gene expression of inflammatory genes in the kidney I/R model. NT=non-treated. *$P<0.05$. Mean±SEM.

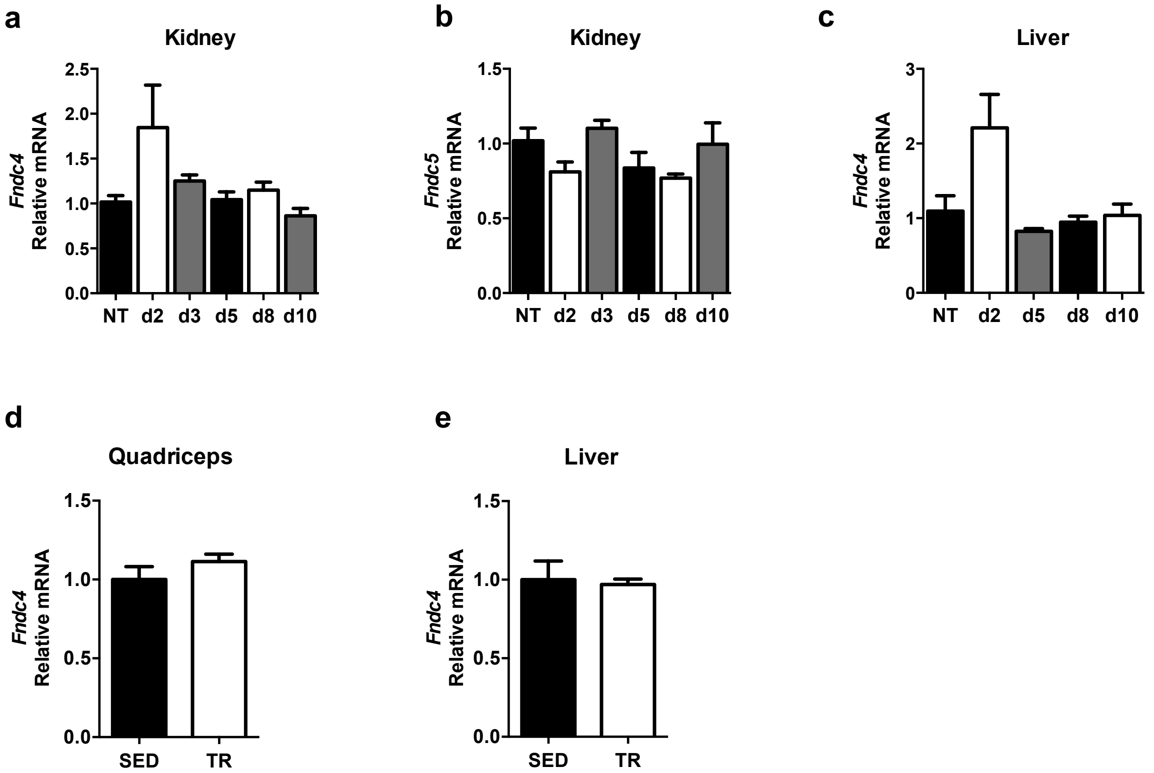

Supplementary Figure 2. *Fndc4 and Fndc5* gene expression in mouse kidney and liver at different timepoints after kidney ischemia/reperfusion. (a) *Fndc4* expression in kidney. (b) *Fndc5* expression in kidney. (c) *Fndc4* expression in liver. Mean±SEM, n=2-3 mice per timepoint. (d-e) Gene expression of *Fndc4* in mouse quadriceps muscle (d) and liver (e) after 2 weeks of progressive swim training (according to Boström et al. 2010, Cell). Mean±SEM, *P<0.05.

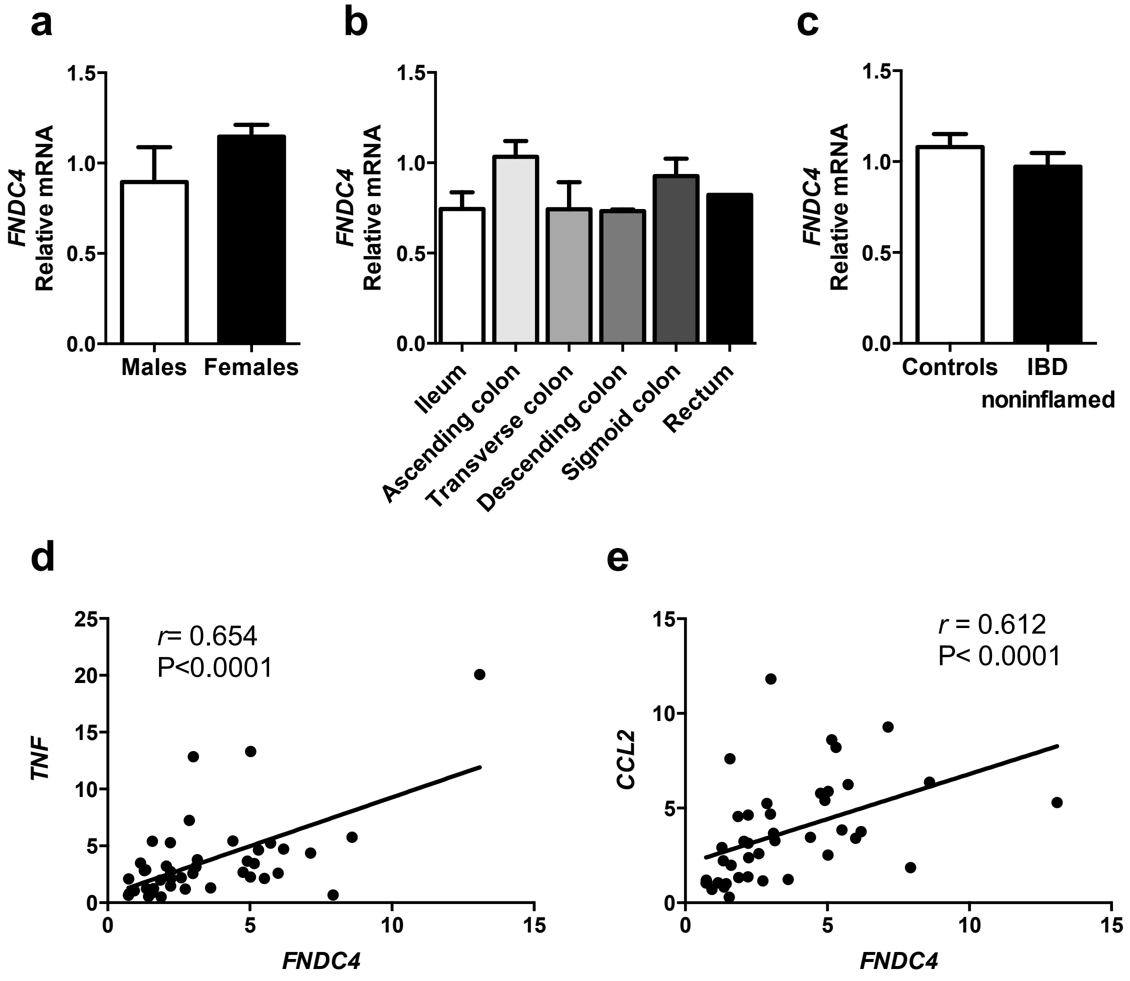

Supplementary Figure 3. Human IBD cohort. (a-b) *FNDC4* gene expression in intestine samples from control subjects; (a) males (n=5) versus females (n=14) and (b) different regions of the intestinal tract. (c) *FNDC4* gene expression in intestine biopsies from control subjects (n=19) versus noninflamed biopsies from IBD-patients (n=40). (d-e) Correlation of *FNDC4* expression with *TNF* (d) and *MCP1* (e) expression. Only inflamed biopsies from IBD subjects were included in the analysis.

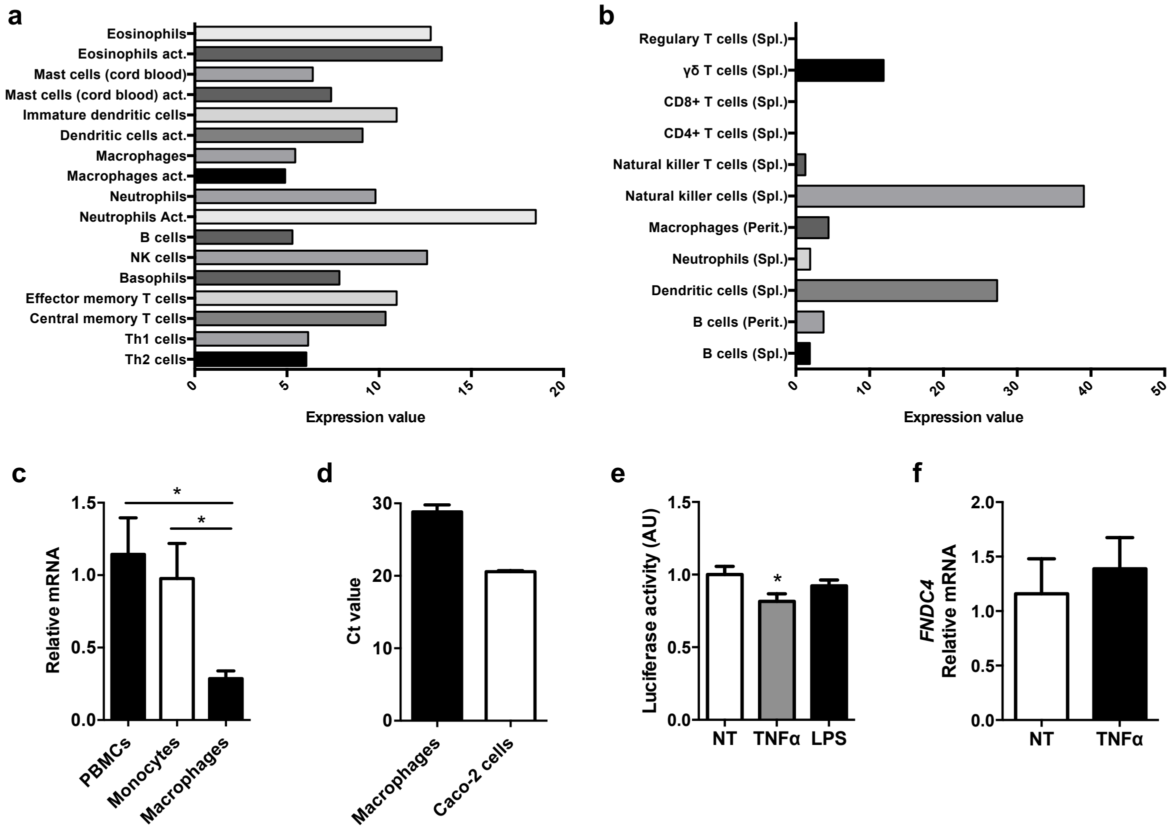

Supplementary Figure 4. *Fndc4* gene expression in different immune cell subtypes. (a) *FNDC4* gene expression in human immune cell subpopulations. Data was derived from the Immunological Genome Project (Immgen). Act: activated. (b) *Fndc4* gene expression in mouse immune cell populations (male C57Bl/6J mice, 6 weeks old). Data from the Immunological Genome Project (Immgen). Spl.: splenic, Perit.: peritoneal. (c) *FNDC4* gene expression in PBMCs (peripheral blood mononuclear cells), monocytes and macrophages from 6 healthy donors. Mean±SEM. *P<0.05. (d) Comparison of *FNDC4* gene expression in Caco-2 colonic epithelial cells and human macrophages. qRT-PCR Ct values (a high Ct value corresponds with a low level of gene expression). (e) Luciferase assay. McA-RH7777 cells were transfected with an *Fndc4* promoter reporter construct and treated with 10ng/mL TNFα or 10 ng/mL LPS for 20h. n=5. Mean±SEM, *P<0.05. (f) *FNDC4* gene expression in Caco-2 cells upon 20h treatment with 10ng/mL TNFα. N=6. Mean±SEM.

a

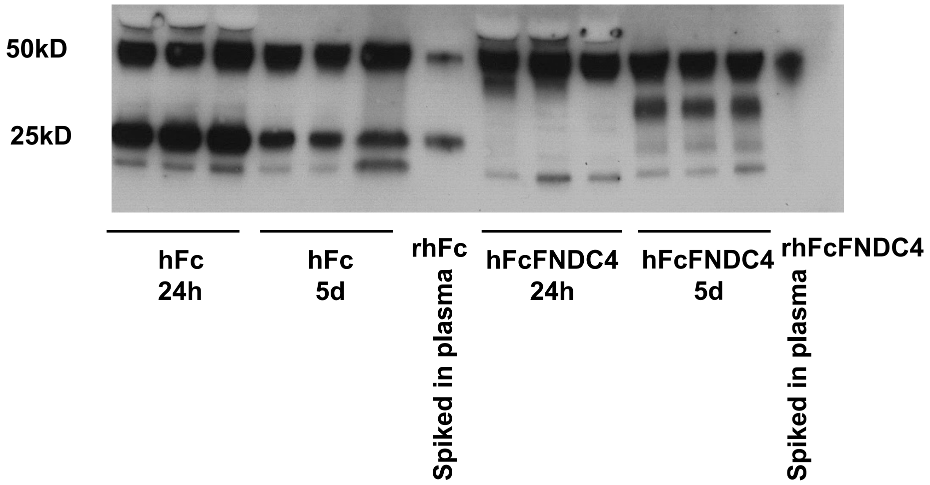

b

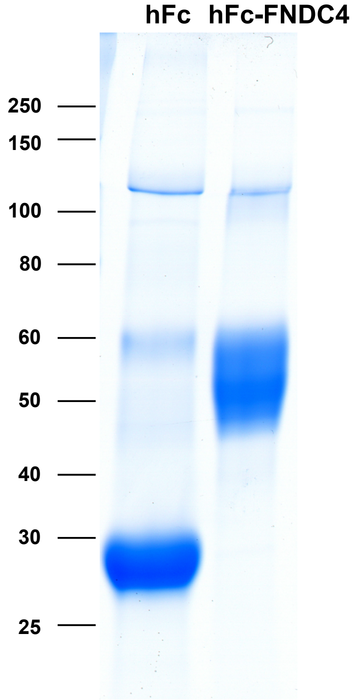

Supplementary Figure 5. Kinetics data for hFc-FNDC4. hFc-FNDC4 or hFc control protein was injected into 9-10 week old C57Bl/6 mice (single injection of 3mg/kg). Plasma was collected after 24h and 5 days and boiled in reducing buffer. Plasma was blotted against hFc (IgG). Recombinant hFc (rhFc) and hFc-FNDC4 (rhFc-FNDC4) were spiked into plasma and treated in the same way as the experimental samples to serve as positive controls. The 50kDa band in the hFc group corresponds to unreduced hFc. The reduced hFc monomer has a molecular weight of approximately 25kDa. The hFc-FNDC4 monomer has a molecular weight of approximately 50kDa. Some degradation/processing products are visible in the plasma of hFc-FNDC4 injected mice after 5 days but not after 24h. Therefore, hFc-FNDC4 and hFc proteins were injected every other day in the DSS cohorts. The full-size blot image is presented in Supplementary Fig. 11d. (b) Coomassie staining demonstrating purity of the hFc and hFc-FNDC4 protein products. 10µg recombinant protein was loaded per lane.

a

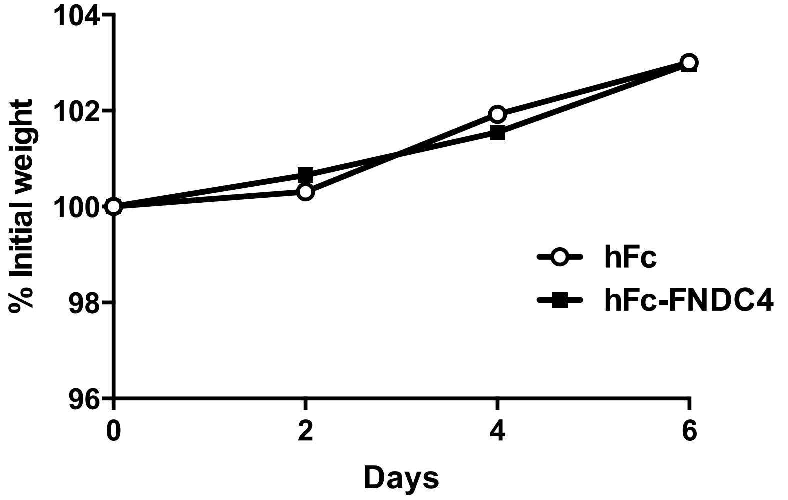

b

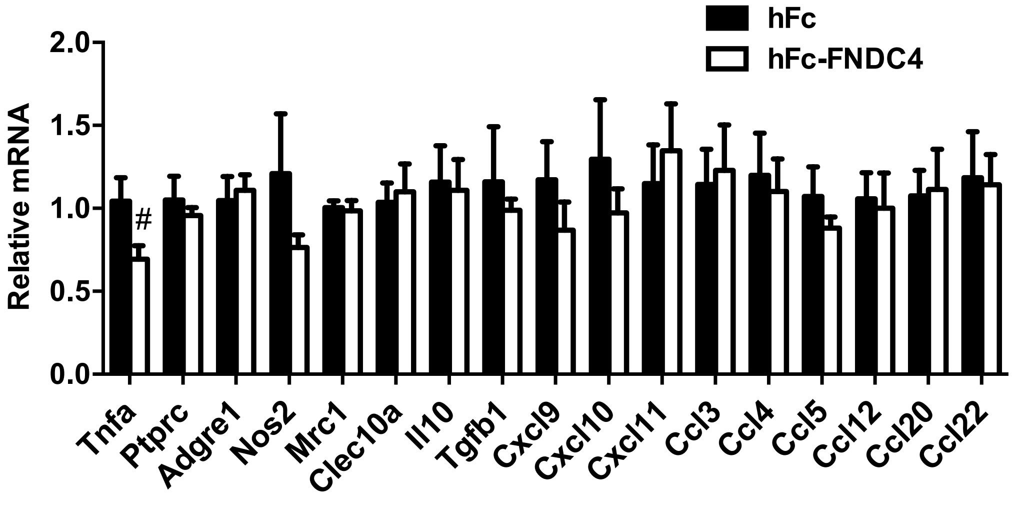

Supplementary Figure 6. hFc-FNDC4 treatment of healthy control mice (no DSS). 80 µg hFc-FNDC4 or hFc control protein was administered every other day over the course of 6 days. (a) Body weight curve. (b) Gene expression of a selection of inflammation-related genes. Mean±SEM. [#]P<0.10.

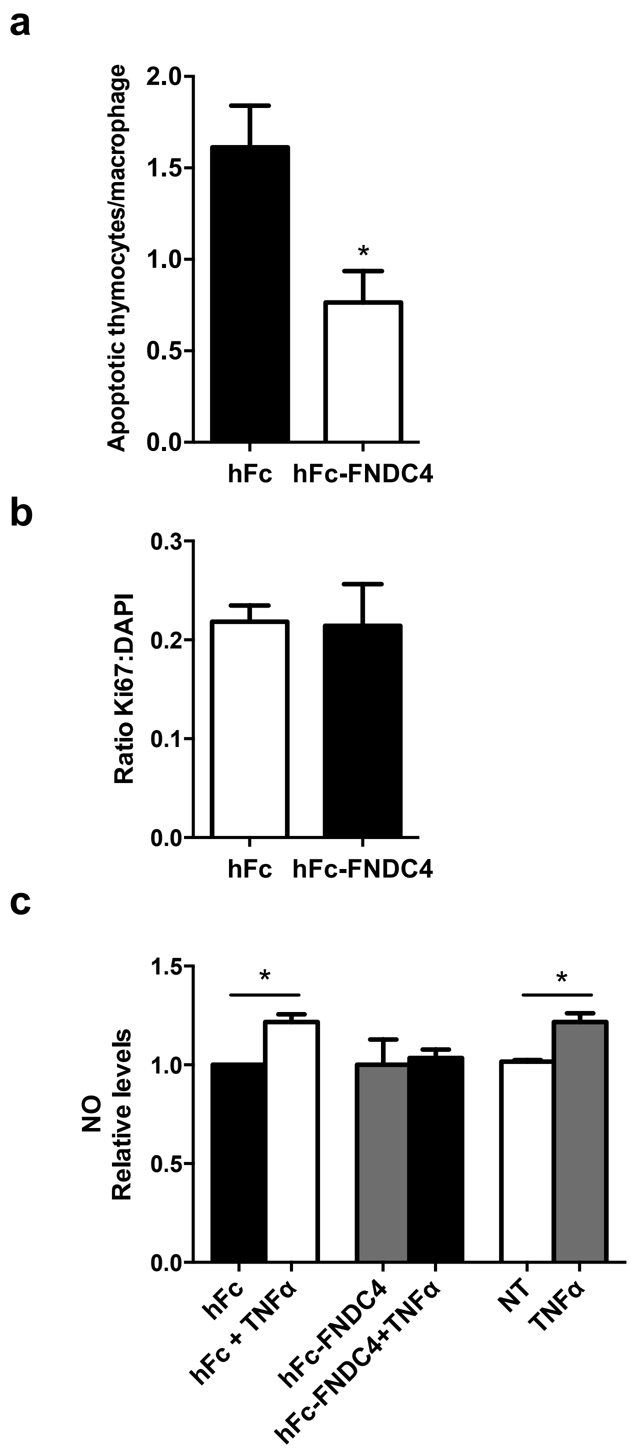

Supplementary Figure 7. (a) Dead cell clearance by hFc-FNDC4 or hFc (100nM, 24h) treated bone marrow macrophages. (b) Ki67 positive nuclei as a measure for proliferation. Cells were treated for 24h with 100nM hFc-FNDC4 or hFc control. (c) NO levels in media of macrophages treated with hFc-FNDC4 or hFc control for 24 in the presence or absence of 10 ng/mL TNFα. Means of 4 independent experiments.

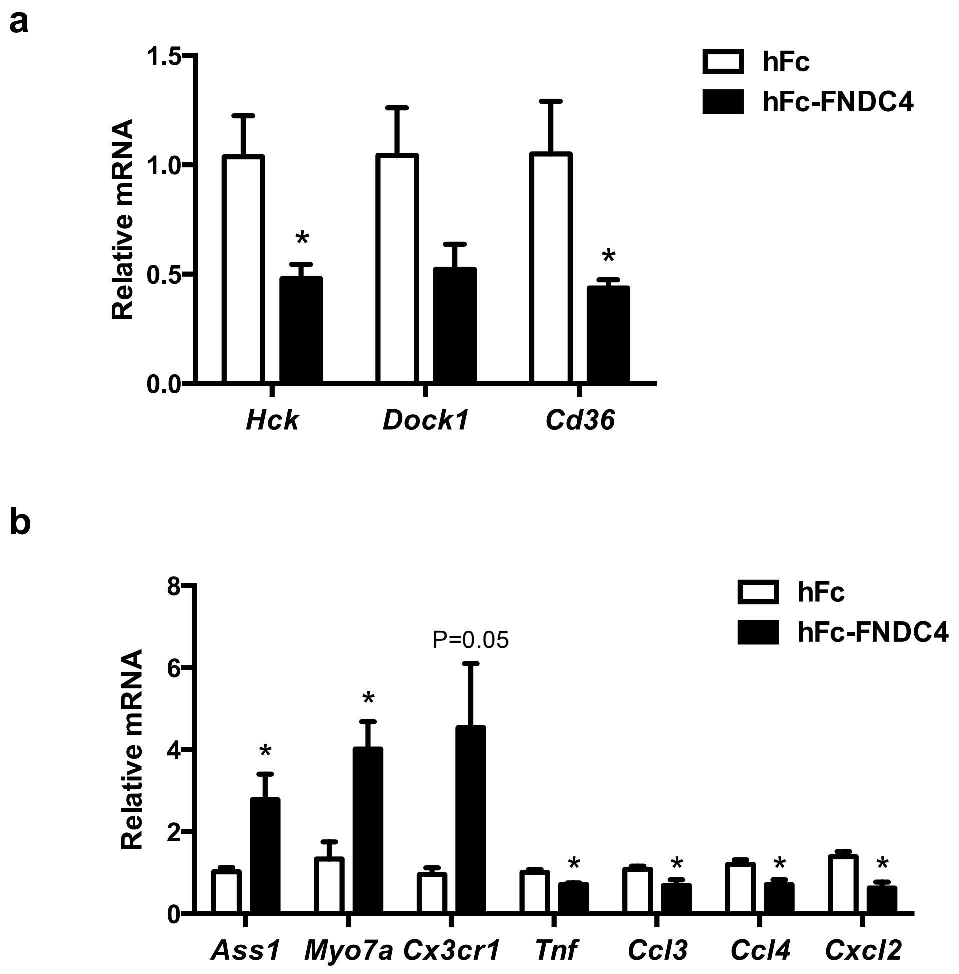

Supplementary Figure 8. (a) Expression of genes involved in phagocytosis in bone marrow derived macrophages treated with 100nM hFc-FNDC4 for 24h. Mean±SEM, n=4 per condition, *P<0.05. (b) Gene expression of a selection of genes in peritoneal macrophages treated with 100μM hFc-FNDC4 or hFc control protein for 5h.

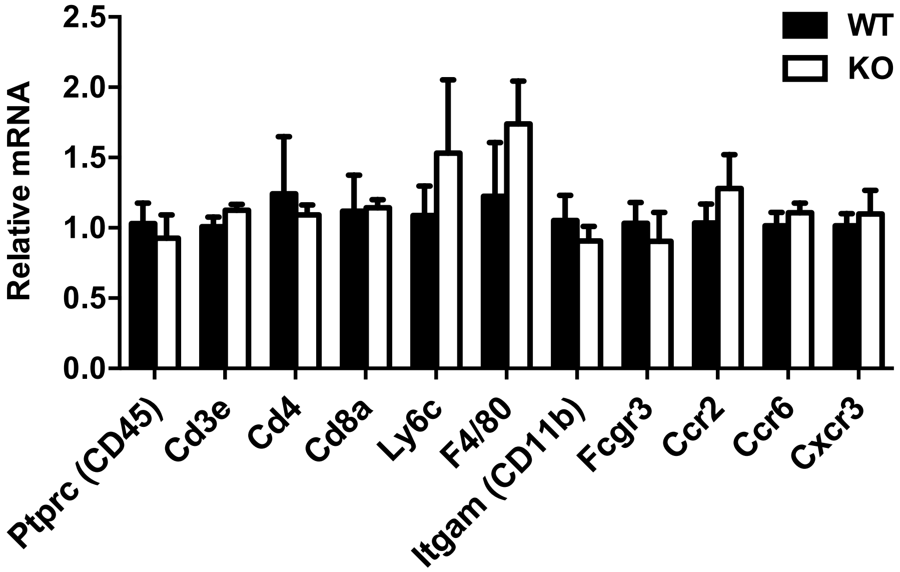

Supplementary Figure 9. Gene expression profiles of immune cell- and inflammation markers in spleen of non-treated *Fndc4* KO and WT mice at 16 weeks of age. Mean±SEM, n=4 per group, *P<0.05.

a

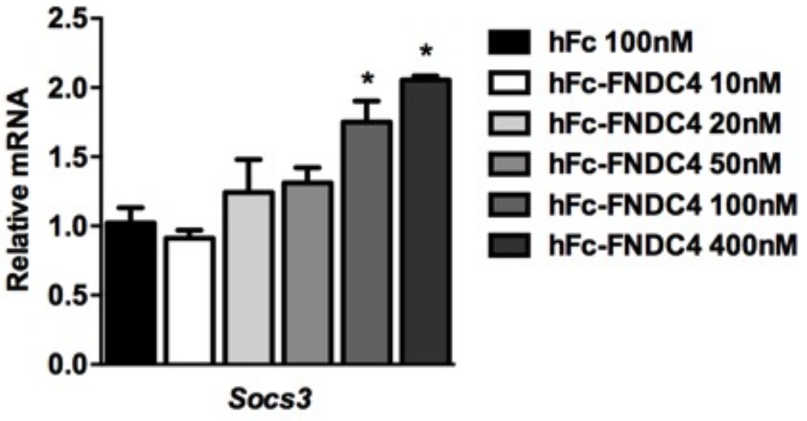

b

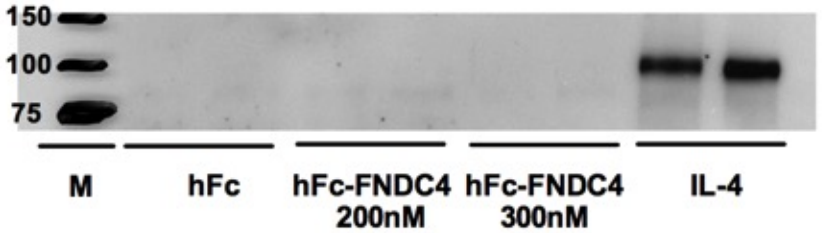

Supplementary Figure 10. (a) *Socs3* gene expression, hFc-FNDC4 dose curve. Bone marrow-derived macrophages were treated with the given concentrations of hFc-FNDC4 or 100nM hFc as control for 24h. Mean±SEM, n=4 per condition. (b) pSTAT6 blotting after 30 minutes treatment with 200nM hFc or hFc-FNDC4 at a concentration of 200 or 300 nM. As positive control, cells were treated with 10 ng/mL interleukin 4. M= marker (protein ladder, highlighted with a pen). The full-size blot image is presented in Supplementary Fig. 11e.

a

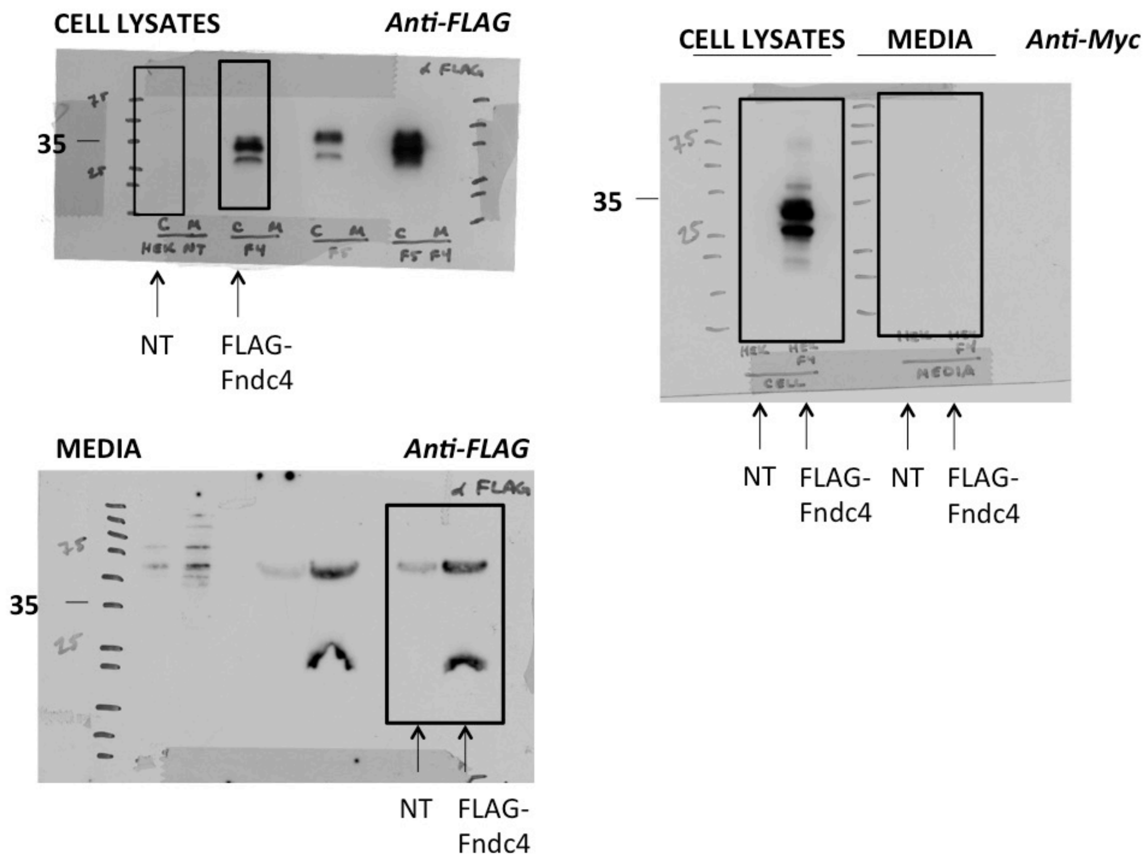
CELL LYSATES
Anti-FLAG
35
NT
FLAG-Fndc4
MEDIA
Anti-FLAG
35
NT
FLAG-Fndc4
CELL LYSATES
MEDIA
Anti-Myc
35
NT
FLAG-Fndc4
NT
FLAG-Fndc4

b

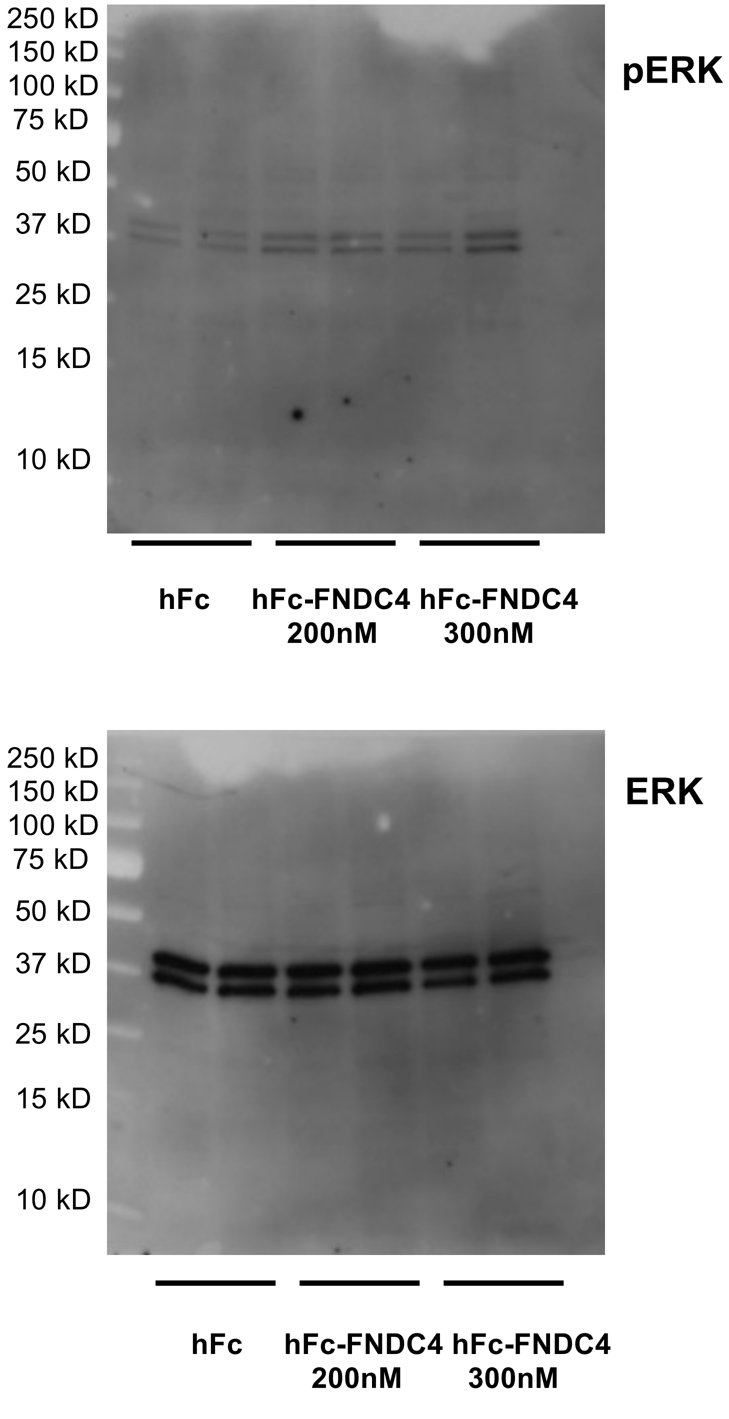
250 kD
150 kD
100 kD
75 kD
50 kD
37 kD
25 kD
15 kD
10 kD
pERK
hFc
hFc-FNDC4
200nM
hFc-FNDC4
300nM
250 kD
150 kD
100 kD
75 kD
50 kD
37 kD
25 kD
15 kD
10 kD
ERK
hFc
hFc-FNDC4
200nM
hFc-FNDC4
300nM

c

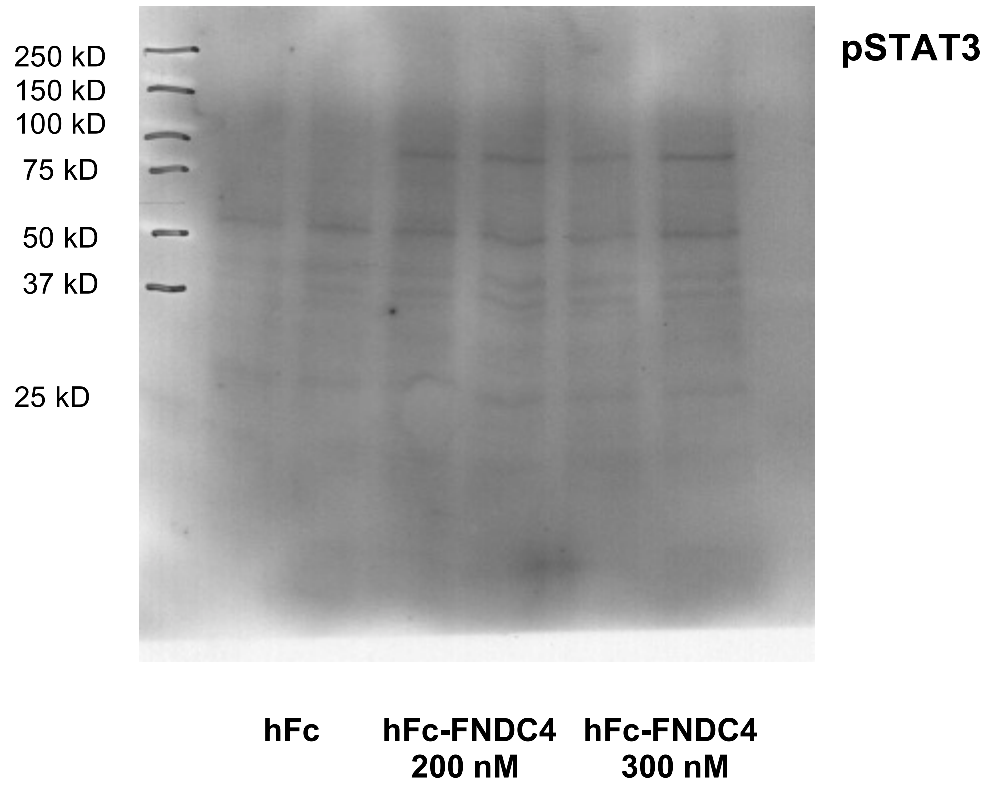
250 kD
150 kD
100 kD
75 kD
50 kD
37 kD
25 kD
pSTAT3
hFc
hFc-FNDC4
200 nM
hFc-FNDC4
300 nM

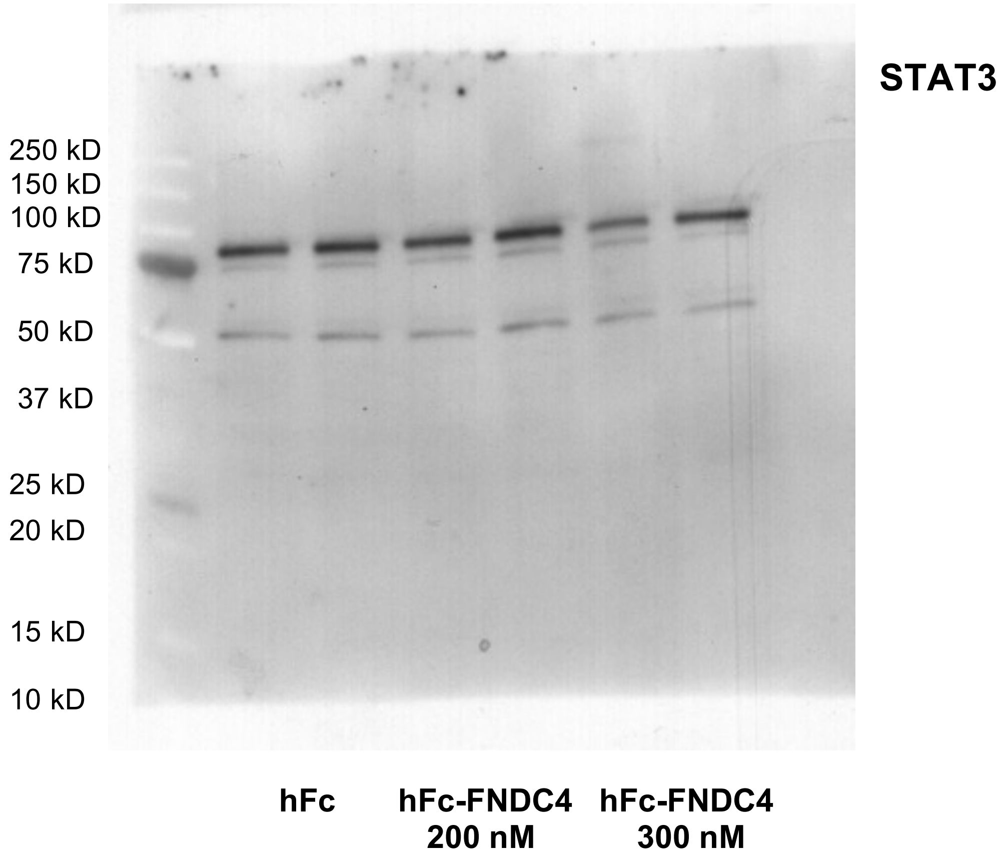
STAT3
250 kD
150 kD
100 kD
75 kD
50 kD
37 kD
25 kD
20 kD
15 kD
10 kD
hFc
hFc-FNDC4
200 nM
hFc-FNDC4
300 nM

d

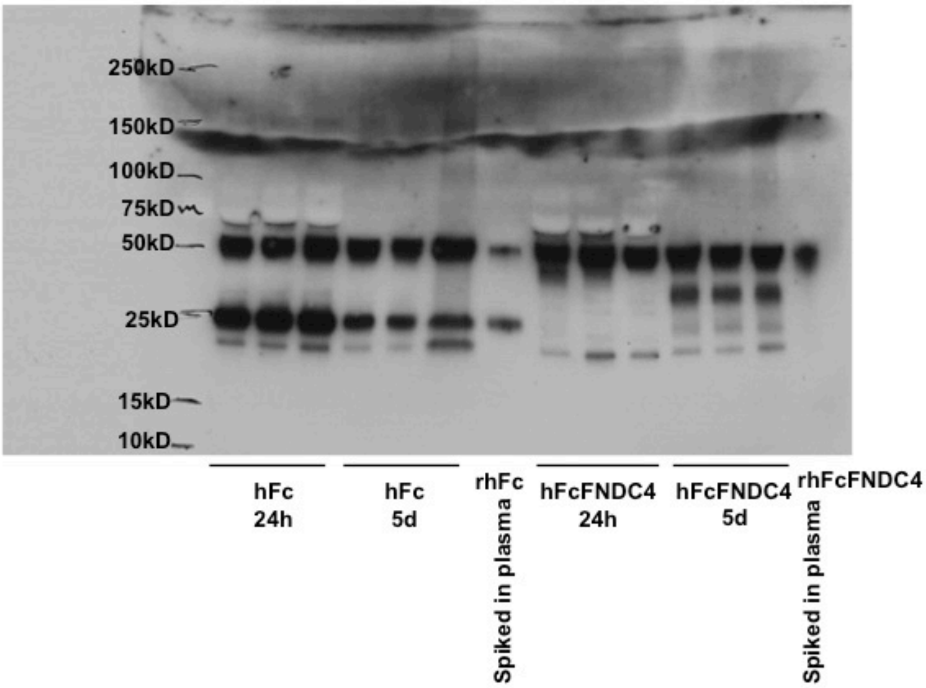

e

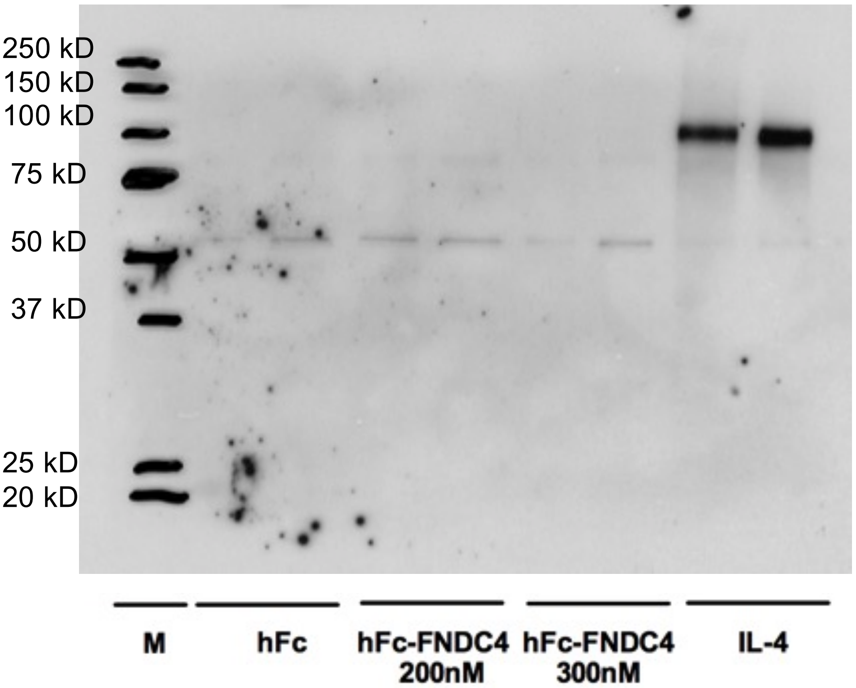

Supplementary Fig. 11. Full-size scan images of Western blots. (a) Uncropped versions of the blots presented in Figure 1b. (b) Uncropped versions of the blots presented in Figure 5c. (c) Uncropped versions of the blots presented in Figure 9b. (d) Uncropped versions of the blots presented in Supplemental Figure 5a. (e) Uncropped versions of the blots presented in Supplemental Figure 10.

Supplementary Table 1. Primer sequences used for qPCR.

	Forward	Reverse
**mAss1**	ACACCTCCTGCATCCTCGT	GCTCACATCCTCAATGAACACCT
**mCcl2**	TTAAAAACCTGGATCGGAACCAA	GCATTAGCTTCAGATTTACGGGT
**mCcl3**	TGTACCATGACACTCTGCAAC	CAACGATGAATTGGCGTGGAA
**mCcl4**	TTCCTGCTGTTTCTCTTACACCT	CTGTCTGCCTCTTTTGGTCAG
**mCd36**	AGATGACGTGGCAAAGAACAG	CCTTGGCTAGATAACGAACTCTG
**mCd3e**	ATGCGGTGGAACACTTTCTGG	GCACGTCAACTCTACACTGGT
**mCd68**	TGTCTGATCTTGCTAGGACCG	GAGAGTAACGGCCTTTTTGTGA
**mClec10a**	CTCTGGAGAGCACAGTGGAG	ACTTCCGAGCCGTTGTTCT
**mCsf1**	GGCTTGGCTTGGGATGATTCT	GAGGGTCTGGCAGGTACTC
**mCx3cr1**	GAGTATGACGATTCTGCTGAGG	CAGACCGAACGTGAAGACGAG
**mCxcl10**	CCAAGTGCTGCCGTCATTTTC	GGCTCGCAGGGATGATTTCAA
**mCxcl2**	CCAACCACCAGGCTACAGG	GCGTCACACTCAAGCTCTG
**mCxcl9**	TCCTTTTGGGCATCATCTTCC	TTTGTAGTGGATCGTGCCTCG
**mDock1**	AGTACGGCGTGGCCTTTTAC	GACGGTTTCATGTTGCCCTTT
**mEmr1**	TGACTCACCTTGTGGTCCTAA	CTTCCCAGAATCCAGTCTTTCC
**mFndc4**	TTCCCCATATCTGAGTCCCAC	TGACCGTCACATTCACAGGAG
**mFndc5**	GGACTCTTGGAAAACACCACTG	TCCACACAGATGATCTCACCAC
**mHck**	TCCTCCGAGATGGAAGCAAG	ACAGTGCGACCACAATGGTAT
**mIl1b**	GCAACTGTTCCTGAACTCAACT	ATCTTTTGGGGTCCGTCAACT
**mItgam**	CCATGACCTTCCAAGAGAATGC	ACCGGCTTGTGCTGTAGTC
**mMrc1**	TGATTACGAGCAGTGGAAGC	GTTCACCGTAAGCCCAATTT
**mMyo7a**	AGGGGGACTATGTATGGATGGA	ATGTGCGTGGCATTCTGAGG
**mNos2**	ACCTTGGTGAAGGGACTGAG	TCCGTTCTCTTGCAGTTGAC
**mPtprc**	GTTTTCGCTACATGACTGCACA	AGGTTGTCCAACTGACATCTTTC
**mSocs3**	ATGGTCACCCACAGCAAGTTT	TCCAGTAGAATCCGCTCTCCT
**mStat3**	CAATACCATTGACCTGCCGAT	GAGCGACTCAAACTGCCCT
**mTbp**	GCTCTGGAATTGTACCGCAG	CTGGCTCATAGCTCTTGGCTC
**mTgfb1**	CTCCCGTGGCTTCTAGTGC	GCCTTAGTTTGGACAGGATCTG
**mTnf**	CCCTCACACTCAGATCATCTTCT	GCTACGACGTGGGCTACAG
**hCCL2**	CAGCCAGATGCAATCAATGCC	TGGAATCCTGAACCCACTTCT
**hCCL3**	AGTTCTCTGCATCACTTGCTG	CGGCTTCGCTTGGTTAGGAA
**hCCL4**	CTGTGCTGATCCCAGTGAATC	TCAGTTCAGTTCCAGGTCATACA
**hCD68**	GTCCACCTCGACCTGCTCT	CACTGGGGCAGGAGAAACT
**hCXCL9**	CCAGTAGTGAGAAAGGGTCGC	AGGGCTTGGGGCAAATTGTT
**hFNDC4**	CACTTCCGAACTCTCTCAAGG	GCAGAACAGCCCAATTACA
**hFNDC5**	GCGGCAGAAGAGAGCTATAACA	ATGAAGGAGATGGGGAGGAA
**hTBP**	CCACTCACAGACTCTCACAAC	CTGCGGTACAATCCCAGAACT
**hTNF**	GAGGCCAAGCCCTGGTATG	CGGGCCGATTGATCTCAGC

Supplementary Table 2. Clinical characteristics of the participants.

	**IBD** (n = 52)	**Non-IBD** (n = 19)
**Disease** (CD / UC / IBDU)	21 / 29 / 2	
**Gender** (male / female)	23 / 29	5/ 14
**Age** (years)[1]	40 (18 – 77)	39 (20 – 69)
**Immune-modulating drugs**		
Aminosalicylates	24	0
Thiopurines	17	0
Corticosteroids	17	0
anti-TNF-α-antibodies	6	0
Methotrexate	2	0
Tacrolimus	2	0
None	10	19

[1] Median (range) values are given.

Supplementary Table 3. Gene expression array data. N=3 samples per group (hFc-FNDC4 treated vs. hFc-treated bone marrow-derived macrophages). Gene selection: Minimum array signal intensity >50; Fold change (FC)>1.5; P<0.05.

Symbol	Gene name	GeneID	FC	P-value	q-value
Serpine1	serine (or cysteine) peptidase inhibitor, clade E, member 1	18787	22.28	<0.001	0.064
Mcam	melanoma cell adhesion molecule	84004	17.33	<0.001	0.018
Fgf2	fibroblast growth factor 2	14173	16.63	<0.001	0.073
Cx3cr1	chemokine (C-X3-C) receptor 1	13051	16.03	<0.001	0.071
AI504432	expressed sequence AI504432	229694	12.59	<0.001	0.073
Tmeff1	transmembrane protein with EGF-like and two follistatin-like domains 1	230157	12.44	<0.001	0.031
Slc36a2	solute carrier family 36 (proton/amino acid symporter), member 2	246049	12.40	0.001	0.141
Kcna3	potassium voltage-gated channel, shaker-related subfamily, member 3	16491	11.51	<0.001	0.067
Ctla2a	cytotoxic T lymphocyte-associated protein 2 alpha	13024	11.47	<0.001	0.069
Pmepa1	prostate transmembrane protein, androgen induced 1	65112	11.31	<0.001	0.018
Egln3	EGL nine homolog 3 (C. elegans)	112407	10.92	<0.001	0.060
Murc	muscle-related coiled-coil protein	68016	10.23	<0.001	0.038
Cspg4	chondroitin sulfate proteoglycan 4	121021	10.04	<0.001	0.073
Gcnt2	glucosaminyl (N-acetyl) transferase 2, I-branching enzyme	14538	9.13	<0.001	0.071
Notch4	Notch gene homolog 4 (Drosophila)	18132	8.23	<0.001	0.067
Cav1	caveolin 1, caveolae protein	12389	7.73	<0.001	0.073
Ly6c1	lymphocyte antigen 6 complex, locus C1	17067	7.52	0.004	0.205
Ly6c2	lymphocyte antigen 6 complex, locus C2	100041546	6.96	0.002	0.168
F11r	F11 receptor	16456	6.72	<0.001	0.048
Ak4	adenylate kinase 4	11639	6.65	<0.001	0.064
Spsb1	splA/ryanodine receptor domain and SOCS box containing 1	74646	6.51	0.002	0.167
Chst3	carbohydrate (chondroitin 6/keratan) sulfotransferase 3	53374	6.25	<0.001	0.060
Gatm	glycine amidinotransferase (L-arginine:glycine amidinotransferase)	67092	5.68	<0.001	0.075
Htr2b	5-hydroxytryptamine (serotonin) receptor 2B	15559	5.46	<0.001	0.048
Fam46c	family with sequence similarity 46, member C	74645	5.43	<0.001	0.048
Ak4	adenylate kinase 4	11639	5.28	<0.001	0.096
Pdlim7	PDZ and LIM domain 7	67399	5.14	0.001	0.112
Cyp4f18	cytochrome P450, family 4, subfamily f, polypeptide 18	72054	4.88	0.001	0.123
Rhov	ras homolog gene family, member V	228543	4.78	<0.001	0.067
Socs2	suppressor of cytokine signaling 2	216233	4.68	0.001	0.128
Ctse	cathepsin E	13034	4.45	0.001	0.154
Ly6i	lymphocyte antigen 6 complex, locus I	57248	4.29	0.002	0.178
Prss46	protease, serine, 46	74306	4.27	0.002	0.156
Pla2g16	phospholipase A2, group XVI	225845	3.91	0.001	0.131
Ptgfrn	prostaglandin F2 receptor negative regulator	19221	3.77	<0.001	0.073
Hp	haptoglobin	15439	3.75	0.002	0.156
Havcr2	hepatitis A virus cellular receptor 2	171285	3.74	0.002	0.156
Cd24a	CD24a antigen	12484	3.64	0.002	0.176
Gm11545	predicted gene 11545	217122	3.62	<0.001	0.082
Ldhb	lactate dehydrogenase B	16832	3.61	0.002	0.156
Plk3	polo-like kinase 3 (Drosophila)	12795	3.47	0.001	0.152
Tmem26	transmembrane protein 26	327766	3.46	0.001	0.113

Cxcl14	chemokine (C-X-C motif) ligand 14	57266	3.37	<0.001	0.073
Cd276	CD276 antigen	102657	3.34	<0.001	0.097
Ppp1r12b	protein phosphatase 1, regulatory (inhibitor) subunit 12B	329251	3.32	<0.001	0.082
Slamf9	SLAM family member 9	98365	3.29	0.004	0.210
Tgfbi	transforming growth factor, beta induced	21810	3.24	<0.001	0.073
Atp2b4	ATPase, Ca++ transporting, plasma membrane 4	381290	3.15	0.001	0.108
Cetn4	centrin 4	207175	3.10	0.002	0.179
Pdgfa	platelet derived growth factor, alpha	18590	3.10	0.002	0.167
Sox4	SRY-box containing gene 4	20677	3.08	0.002	0.156
Ctla2b	cytotoxic T lymphocyte-associated protein 2 beta	13025	3.08	<0.001	0.018
Gpr84	G protein-coupled receptor 84	80910	3.08	0.002	0.166
Angptl2	angiopoietin-like 2	26360	3.07	<0.001	0.092
Pbxip1	pre-B-cell leukemia transcription factor interacting protein 1	229534	3.07	0.004	0.210
Socs2	suppressor of cytokine signaling 2	216233	3.07	0.002	0.156
Lrrc16a	leucine rich repeat containing 16A	68732	3.02	<0.001	0.060
Fads2	fatty acid desaturase 2	56473	2.95	0.010	0.305
X99384	cDNA sequence X99384	27355	2.90	0.001	0.100
Olfml3	olfactomedin-like 3	99543	2.90	<0.001	0.096
Ndrg2	N-myc downstream regulated gene 2	29811	2.84	0.034	0.469
Slc16a3	solute carrier family 16 (monocarboxylic acid transporters), member 3	80879	2.83	0.001	0.127
Zfp618	zinc fingerprotein 618	72701	2.82	<0.001	0.082
Bach2	BTB and CNC homology 2	12014	2.82	<0.001	0.080
Col14a1	collagen, type XIV, alpha 1	12818	2.80	0.002	0.167
Stk38l	serine/threonine kinase 38 like	232533	2.80	<0.001	0.073
Jag1	jagged 1	16449	2.80	0.001	0.127
Ptgs1	prostaglandin-endoperoxide synthase 1	19224	2.77	0.003	0.196
Ldhb	lactate dehydrogenase B	16832	2.77	0.003	0.196
Bcar3	breast cancer anti-estrogen resistance 3	29815	2.76	0.001	0.131
Slc7a4	solute carrier family 7 (cationic amino acid transporter, y+ system), member 4	224022	2.74	0.004	0.227
Ttc28	tetratricopeptide repeat domain 28	209683	2.73	0.001	0.109
Rai14	retinoic acid induced 14	75646	2.73	<0.001	0.085
Eya4	eyes absent 4 homolog (Drosophila)	14051	2.69	<0.001	0.073
Mmp2	matrix metallopeptidase 2	17390	2.69	<0.001	0.073
Vdr	vitamin D receptor	22337	2.68	0.008	0.280
Flt1	FMS-like tyrosine kinase 1	14254	2.68	0.001	0.102
Hgf	hepatocyte growth factor	15234	2.68	<0.001	0.069
Arg2	arginase type II	11847	2.68	0.004	0.224
Il21r	interleukin 21 receptor	60504	2.65	<0.001	0.023
Xylt1	xylosyltransferase 1	233781	2.64	<0.001	0.073
Ttc28	tetratricopeptide repeat domain 28	209683	2.64	<0.001	0.048
Ltc4s	leukotriene C4 synthase	17001	2.63	0.008	0.275
Bhlhe40	basic helix-loop-helix family, member e40	20893	2.62	0.001	0.140
Haus8	4HAUS augmin-like complex, subunit 8	76478	2.61	0.001	0.112
Ass1	argininosuccinate synthetase 1	11898	2.60	0.001	0.127
Gpr157	G protein-coupled receptor 157	269604	2.59	<0.001	0.064
Ankrd37	ankyrin repeat domain 37	654824	2.59	0.001	0.105

Fkbp5	FK506 binding protein 5	14229	2.59	0.002	0.174
D18Ertd653e	DNA segment, Chr 18, ERATO Doi 653, expressed	52662	2.57	<0.001	0.096
Ass1	argininosuccinate synthetase 1	11898	2.57	0.001	0.127
Gpr183	G protein-coupled receptor 183	321019	2.56	0.004	0.223
Gtf2h2	general transcription factor II H, polypeptide 2	23894	2.54	<0.001	0.075
Ttc28	tetratricopeptide repeat domain 28	209683	2.51	<0.001	0.097
Trem1	triggering receptor expressed on myeloid cells 1	58217	2.50	0.006	0.253
Gna15	guanine nucleotide binding protein, alpha 15	14676	2.49	<0.001	0.078
Fzd7	frizzled homolog 7 (Drosophila)	14369	2.49	<0.001	0.073
Angptl4	angiopoietin-like 4	57875	2.47	0.002	0.156
Elk3	ELK3, member of ETS oncogene family	13713	2.45	<0.001	0.067
Fmnl3	formin-like 3	22379	2.45	0.001	0.115
Zfp691	zinc finger protein 691	195522	2.43	0.003	0.189
Klhl6	kelch-like 6 (Drosophila)	239743	2.42	0.001	0.146
1700017B05Rik	RIKEN cDNA 1700017B05 gene	74211	2.42	<0.001	0.082
Usp2	ubiquitin specific peptidase 2	53376	2.41	0.002	0.156
Ankrd57	ankyrin repeat domain 57	268301	2.39	<0.001	0.067
Chd7	chromodomain helicase DNA binding protein 7	320790	2.37	0.002	0.156
Gm10002	predicted gene 10002	791405	2.37	0.004	0.214
Chd7	chromodomain helicase DNA binding protein 7	320790	2.35	0.003	0.185
Hexb	hexosaminidase B	15212	2.35	<0.001	0.073
Procr	protein C receptor, endothelial	19124	2.33	<0.001	0.096
Chd7	chromodomain helicase DNA binding protein 7	320790	2.33	0.001	0.105
Hes1	hairy and enhancer of split 1 (Drosophila)	15205	2.31	<0.001	0.060
E2f2	E2F transcription factor 2	242705	2.31	0.003	0.202
9030425E11Rik	RIKEN cDNA 9030425E11 gene	71566	2.31	0.014	0.338
2010002N04Rik	RIKEN cDNA 2010002N04 gene	106878	2.29	0.003	0.188
Chd7	chromodomain helicase DNA binding protein 7	320790	2.28	0.008	0.274
Dmxl2	Dmx-like 2	235380	2.26	<0.001	0.075
Prr5l	proline rich 5 like	72446	2.25	0.002	0.167
Runx2	runt related transcription factor 2	12393	2.25	0.003	0.186
Sgk1	serum/glucocorticoid regulated kinase 1	20393	2.24	0.002	0.178
S1pr1	sphingosine-1-phosphate receptor 1	13609	2.24	<0.001	0.082
Cfb	complement factor B	14962	2.23	0.020	0.397
Plau	plasminogen activator, urokinase	18792	2.22	0.001	0.131
Luzp1	leucine zipper protein 1	269593	2.22	0.001	0.102
Chd7	chromodomain helicase DNA binding protein 7	320790	2.22	0.001	0.115
Enc1	ectodermal-neural cortex 1	13803	2.22	<0.001	0.073
Spata13	spermatogenesis associated 13	219140	2.22	<0.001	0.073
Loxl2	lysyl oxidase-like 2	94352	2.21	<0.001	0.084
Slc25a37	solute carrier family 25, member 37	67712	2.21	0.002	0.156
Tagap	T-cell activation Rho GTPase-activating protein	72536	2.20	0.002	0.156
AI427809	expressed sequence AI427809	381524	2.20	0.004	0.210
Pdgfb	platelet derived growth factor, B polypeptide	18591	2.19	0.001	0.111
Plekhh2	pleckstrin homology domain containing, family H (with MyTH4 domain) member 2	213556	2.18	0.002	0.176

Rilpl1	Rab interacting lysosomal protein-like 1	75695	2.18	0.003	0.185
Chd7	chromodomain helicase DNA binding protein 7	320790	2.17	0.001	0.127
Ang2	angiogenin, ribonuclease A family, member 2	11731	2.17	<0.001	0.073
Mgat4a	mannoside acetylglucosaminyltransferase 4, isoenzyme A	269181	2.17	<0.001	0.073
Chd7	chromodomain helicase DNA binding protein 7	320790	2.16	0.002	0.158
Slamf6	SLAM family member 6	30925	2.15	0.001	0.127
Armc9	armadillo repeat containing 9	78795	2.15	0.005	0.228
Agap1	ArfGAP with GTPase domain, ankyrin repeat and PH domain 1	347722	2.15	<0.001	0.073
Cd109	CD109 antigen	235505	2.15	<0.001	0.073
Chd7	chromodomain helicase DNA binding protein 7	320790	2.15	0.002	0.178
Fat3	FAT tumor suppressor homolog 3 (Drosophila)	270120	2.14	<0.001	0.049
Prune2	prune homolog 2 (Drosophila)	353211	2.13	0.008	0.275
Epb4.1l2	erythrocyte protein band 4.1-like 2	13822	2.13	0.001	0.108
Zranb3	zinc finger, RAN-binding domain containing 3	226409	2.12	0.001	0.144
Map3k9	mitogen-activated protein kinase kinase kinase 9	338372	2.11	0.001	0.141
Smad7	MAD homolog 7 (Drosophila)	17131	2.11	<0.001	0.094
Fcrls	Fc receptor-like S, scavenger receptor	80891	2.10	0.007	0.269
Rgs11	regulator of G-protein signaling 11	50782	2.10	0.017	0.372
5730528L 13Rik	RIKEN cDNA 5730528L13 gene	66665	2.09	0.004	0.223
D8Ertd82e	DNA segment, Chr 8, ERATO Doi 82, expressed	244418	2.09	0.001	0.112
Chd7	chromodomain helicase DNA binding protein 7	320790	2.08	<0.001	0.073
Dcakd	dephospho-CoA kinase domain containing	68087	2.08	0.002	0.156
Ch25h	cholesterol 25-hydroxylase	12642	2.08	0.028	0.440
Rap2a	RAS related protein 2a	76108	2.08	0.001	0.116
Chd7	chromodomain helicase DNA binding protein 7	320790	2.07	0.005	0.240
Chd7	chromodomain helicase DNA binding protein 7	320790	2.07	0.001	0.116
Bbs12	Bardet-Biedl syndrome 12 (human)	241950	2.07	<0.001	0.073
Mir23b	microRNA 23b	387217	2.06	0.002	0.177
Fat3	FAT tumor suppressor homolog 3 (Drosophila)	270120	2.06	<0.001	0.075
Cd300lf	CD300 antigen like family member F	246746	2.06	0.006	0.245
Vhl	von Hippel-Lindau tumor suppressor	22346	2.05	0.005	0.227
Arhgap24	Rho GTPase activating protein 24	231532	2.05	0.001	0.108
Chd7	chromodomain helicase DNA binding protein 7	320790	2.05	0.002	0.168
Chd7	chromodomain helicase DNA binding protein 7	320790	2.04	0.003	0.193
Chd7	chromodomain helicase DNA binding protein 7	320790	2.04	<0.001	0.073
Itga5	integrin alpha 5 (fibronectin receptor alpha)	16402	2.04	<0.001	0.096
Galnt7	UDP-N-acetyl-alpha-D-galactosamine: polypeptide N-acetylgalactosaminyltransferase 7	108150	2.03	0.001	0.115
Mfge8	milk fat globule-EGF factor 8 protein	17304	2.02	0.005	0.239
Zfp503	zinc finger protein 503	218820	2.02	0.002	0.165
Chd7	chromodomain helicase DNA binding protein 7	320790	2.02	0.004	0.215
Nptx1	neuronal pentraxin 1	18164	2.01	0.020	0.391
Chd7	chromodomain helicase DNA binding protein 7	320790	2.01	0.004	0.206
Uchl1	ubiquitin carboxy-terminal hydrolase L1	22223	1.99	0.021	0.403
Tle1	transducin-like enhancer of split 1, homolog of Drosophila E(spl)	21885	1.99	0.001	0.119
Hopx	HOP homeobox	74318	1.99	0.003	0.186
Cpd	carboxypeptidase D	12874	1.99	<0.001	0.082

Dmxl2	Dmx-like 2	235380	1.99	0.002	0.156
Pfkl	phosphofructokinase, liver, B-type	18641	1.99	0.004	0.224
Extl3	exostoses (multiple)-like 3	54616	1.99	0.008	0.275
Rttn	rotatin	246102	1.97	<0.001	0.073
Gm4980	predicted gene 4980	245190	1.97	<0.001	0.075
Olfml2b	olfactomedin-like 2B	320078	1.97	0.024	0.416
Sdc1	syndecan 1	20969	1.96	0.003	0.185
Chd7	chromodomain helicase DNA binding protein 7	320790	1.96	0.001	0.129
Mr1	major histocompatibility complex, class I-related	15064	1.96	0.003	0.184
Asap3	ArfGAP with SH3 domain, ankyrin repeat and PH domain 3	230837	1.96	0.002	0.162
6430527G18Rik	RIKEN cDNA 6430527G18 gene	238330	1.96	0.001	0.125
Chd7	chromodomain helicase DNA binding protein 7	320790	1.96	0.001	0.139
Chd7	chromodomain helicase DNA binding protein 7	320790	1.95	0.002	0.165
Socs6	suppressor of cytokine signaling 6	54607	1.95	<0.001	0.073
Chd7	chromodomain helicase DNA binding protein 7	320790	1.94	0.003	0.183
Itga6	integrin alpha 6	16403	1.94	0.002	0.169
Fat3	FAT tumor suppressor homolog 3 (Drosophila)	270120	1.94	<0.001	0.097
Tgfbr1	transforming growth factor, beta receptor I	21812	1.94	0.002	0.162
Socs3	suppressor of cytokine signaling 3	12702	1.93	0.001	0.127
Bnip3	BCL2/adenovirus E1B interacting protein 3	12176	1.93	<0.001	0.073
Chd7	chromodomain helicase DNA binding protein 7	320790	1.92	0.003	0.184
Chd7	chromodomain helicase DNA binding protein 7	320790	1.92	0.002	0.170
Ccl5	chemokine (C-C motif) ligand 5	20304	1.92	0.003	0.204
Chd7	chromodomain helicase DNA binding protein 7	320790	1.92	<0.001	0.067
Hivep3	human immunodeficiency virus type I enhancer binding protein 3	16656	1.91	0.001	0.146
Fat3	FAT tumor suppressor homolog 3 (Drosophila)	270120	1.91	<0.001	0.075
Shisa9	shisa homolog 9 (Xenopus laevis)	72555	1.91	<0.001	0.095
Chd7	chromodomain helicase DNA binding protein 7	320790	1.90	0.001	0.117
Mir27b	microRNA 27b	387221	1.90	0.002	0.156
Gm22	predicted gene 22	195209	1.90	<0.001	0.067
Dna2	DNA replication helicase 2 homolog (yeast)	327762	1.89	0.005	0.228
Pfkfb3	6-phosphofructo-2-kinase/fructose-2,6-biphosphatase 3	170768	1.89	0.001	0.127
Sipa1l2	signal-induced proliferation-associated 1 like 2	244668	1.89	0.001	0.127
Neto2	neuropilin (NRP) and tolloid (TLL)-like 2	74513	1.89	0.004	0.210
Chd7	chromodomain helicase DNA binding protein 7	320790	1.89	0.001	0.103
Fat3	FAT tumor suppressor homolog 3 (Drosophila)	270120	1.89	0.001	0.146
Dcakd	dephospho-CoA kinase domain containing	68087	1.89	0.005	0.228
Sh3d20	SH3 domain containing 20	70559	1.88	0.002	0.178
Mir24-1	microRNA 24-1	387142	1.88	0.007	0.269
Synj2	synaptojanin 2	20975	1.88	0.003	0.196
Chd7	chromodomain helicase DNA binding protein 7	320790	1.88	0.004	0.216
Fat3	FAT tumor suppressor homolog 3 (Drosophila)	270120	1.87	0.017	0.364
Fat3	FAT tumor suppressor homolog 3 (Drosophila)	270120	1.87	0.002	0.156
Lrrk2	leucine-rich repeat kinase 2	66725	1.87	0.002	0.159
Thap7	THAP domain containing 7	69009	1.87	0.021	0.401
Pdpn	podoplanin	14726	1.87	<0.001	0.064

Syt11	synaptotagmin XI	229521	1.87	0.001	0.105
Tmem119	transmembrane protein 119	231633	1.87	0.012	0.325
Socs6	suppressor of cytokine signaling 6	54607	1.87	0.001	0.112
Rnase4	ribonuclease, RNase A family 4	58809	1.86	0.004	0.210
Chd7	chromodomain helicase DNA binding protein 7	320790	1.86	0.001	0.119
Itgav	integrin alpha V	16410	1.84	0.003	0.196
Tnfrsf12a	tumor necrosis factor receptor superfamily, member 12a	27279	1.84	0.006	0.250
Cetn4	centrin 4	207175	1.84	0.010	0.303
1600014C10Rik	RIKEN cDNA 1600014C10 gene	72244	1.84	0.026	0.426
Hmga2	high mobility group AT-hook 2	15364	1.84	0.001	0.149
Rab3b	RAB3B, member RAS oncogene family	69908	1.84	0.005	0.228
Fat3	FAT tumor suppressor homolog 3 (Drosophila)	270120	1.83	<0.001	0.096
Cd207	CD207 antigen	246278	1.83	0.043	0.497
Chd7	chromodomain helicase DNA binding protein 7	320790	1.82	0.003	0.200
Osbpl3	oxysterol binding protein-like 3	71720	1.82	0.005	0.227
Chd7	chromodomain helicase DNA binding protein 7	320790	1.81	<0.001	0.073
Btbd19	BTB (POZ) domain containing 19	78611	1.81	0.038	0.477
Cav2	caveolin 2	12390	1.81	0.002	0.167
Znrf3	zinc and ring finger 3	407821	1.81	0.006	0.253
Phyhd1	phytanoyl-CoA dioxygenase domain containing 1	227696	1.80	0.025	0.420
Abi3	ABI gene family, member 3	66610	1.80	0.004	0.223
Clip2	CAP-GLY domain containing linker protein 2	269713	1.80	0.002	0.167
Plcb4	phospholipase C, beta 4	18798	1.79	0.013	0.333
Runx3	runt related transcription factor 3	12399	1.79	0.006	0.247
Tmem37	transmembrane protein 37	170706	1.79	0.005	0.231
Bcl2	B-cell leukemia/lymphoma 2	12043	1.79	0.019	0.386
Vipr1	vasoactive intestinal peptide receptor 1	22354	1.79	0.014	0.338
Mir34a	microRNA 34a	723848	1.79	0.012	0.328
Gcnt1	glucosaminyl (N-acetyl) transferase 1, core 2	14537	1.79	0.002	0.171
Cpeb2	cytoplasmic polyadenylation element binding protein 2	231207	1.78	<0.001	0.073
Arrdc3	arrestin domain containing 3	105171	1.78	0.002	0.167
Hk2	hexokinase 2	15277	1.78	0.005	0.238
Cd9	CD9 antigen	12527	1.77	0.005	0.227
Ssh2	slingshot homolog 2 (Drosophila)	237860	1.77	0.001	0.105
Lrrc16a	leucine rich repeat containing 16A	68732	1.76	0.003	0.200
Ctxn1	cortexin 1	330695	1.75	0.001	0.121
Fat3	FAT tumor suppressor homolog 3 (Drosophila)	270120	1.75	<0.001	0.083
Fat3	FAT tumor suppressor homolog 3 (Drosophila)	270120	1.75	0.001	0.109
Fhod1	formin homology 2 domain containing 1	234686	1.75	0.008	0.276
Nfil3	nuclear factor, interleukin 3, regulated	18030	1.75	0.014	0.338
Sulf2	sulfatase 2	72043	1.75	0.001	0.105
Inf2	inverted formin, FH2 and WH2 domain containing	70435	1.75	0.002	0.156
Gm14005	predicted gene 14005	100043424	1.75	0.002	0.167
Tmx4	thioredoxin-related transmembrane protein 4	52837	1.74	0.006	0.243
B4galt1	UDP-Gal:betaGlcNAc beta 1,4- galactosyltransferase, polypeptide 1	14595	1.73	0.006	0.250
Adcy2	adenylate cyclase 2	210044	1.73	0.002	0.161

Bnip3	BCL2/adenovirus E1B interacting protein 3	12176	1.73	0.001	0.132
Pdgfrl	platelet-derived growth factor receptor-like	68797	1.73	0.009	0.294
5031414D18Rik	RIKEN cDNA 5031414D18 gene	271221	1.73	0.012	0.328
Luzp1	leucine zipper protein 1	269593	1.73	<0.001	0.073
Ets1	E26 avian leukemia oncogene 1, 5' domain	23871	1.72	0.010	0.297
Cd101	CD101 antigen	630146	1.72	0.029	0.450
Fat3	FAT tumor suppressor homolog 3 (Drosophila)	270120	1.72	<0.001	0.078
Gm16432	predicted gene 16432	545391	1.72	0.003	0.181
Mtus1	mitochondrial tumor suppressor 1	102103	1.72	0.002	0.156
Ppcdc	phosphopantothenoylcysteine decarboxylase	66812	1.72	0.017	0.370
Psat1	phosphoserine aminotransferase 1	107272	1.72	0.001	0.139
Vwf	Von Willebrand factor homolog	22371	1.71	0.006	0.243
Gins1	GINS complex subunit 1 (Psf1 homolog)	69270	1.71	0.010	0.295
Gpr56	G protein-coupled receptor 56	14766	1.71	0.019	0.388
D10Wsu52e	DNA segment, Chr 10, Wayne State University 52, expressed	28088	1.70	0.002	0.176
Chd7	chromodomain helicase DNA binding protein 7	320790	1.70	0.004	0.221
Nlrc3	NLR family, CARD domain containing 3	268857	1.70	0.011	0.313
Mical2	microtubule associated monoxygenase, calponin and LIM domain containing 2	320878	1.70	0.001	0.104
Kcnn4	potassium intermediate/small conductance calcium-activated channel, subfamily N, member 4	16534	1.69	0.019	0.387
Itgb5	integrin beta 5	16419	1.69	0.003	0.204
Myo1d	myosin ID	338367	1.69	0.008	0.274
Gpr77	G protein-coupled receptor 77	319430	1.69	0.018	0.372
Grhpr	glyoxylate reductase/hydroxypyruvate reductase	76238	1.68	0.005	0.228
Ctsh	cathepsin H	13036	1.68	0.002	0.162
Skil	SKI-like	20482	1.68	0.004	0.210
Golm1	golgi membrane protein 1	105348	1.67	0.017	0.368
Vash1	vasohibin 1	238328	1.67	0.002	0.156
Bmyc	brain expressed myelocytomatosis oncogene	107771	1.66	0.009	0.290
Apoc2	apolipoprotein C-II	11813	1.66	0.017	0.368
Pcdh7	protocadherin 7	54216	1.66	0.008	0.274
Rcan1	regulator of calcineurin 1	54720	1.66	0.010	0.305
Gde1	glycerophosphodiester phosphodiesterase 1	56209	1.66	0.003	0.187
Galns	galactosamine (N-acetyl)-6-sulfate sulfatase	50917	1.66	0.001	0.126
Phospho1	phosphatase, orphan 1	237928	1.65	0.005	0.227
Fat3	FAT tumor suppressor homolog 3 (Drosophila)	270120	1.65	0.004	0.224
Scoc	short coiled-coil protein	56367	1.65	0.002	0.176
Apbb2	amyloid beta (A4) precursor protein-binding, family B, member 2	11787	1.65	0.006	0.253
Agfg2	ArfGAP with FG repeats 2	231801	1.65	0.005	0.228
P4ha1	procollagen-proline, 2-oxoglutarate 4-dioxygenase (proline 4-hydroxylase), alpha 1 polypeptide	18451	1.65	0.006	0.254
1300002K09Rik	RIKEN cDNA 1300002K09 gene	74152	1.65	0.027	0.433
Nfix	nuclear factor I/X	18032	1.65	0.023	0.407
Lyzl4	lysozyme-like 4	69032	1.64	0.017	0.368
Tatdn2	TatD DNase domain containing 2	381801	1.64	0.008	0.274
Oit3	oncoprotein induced transcript 3	18302	1.64	0.011	0.321
Arrdc4	arrestin domain containing 4	66412	1.63	0.005	0.227

4931406P16Rik	RIKEN cDNA 4931406P16 gene	233103	1.63	0.004	0.220
Rassf3	Ras association (RalGDS/AF-6) domain family member 3	192678	1.63	<0.001	0.064
Slc27a1	solute carrier family 27 (fatty acid transporter), member 1	26457	1.63	0.013	0.335
Zfp608	zinc finger protein 608	269023	1.63	0.008	0.276
Pde3b	phosphodiesterase 3B, cGMP-inhibited	18576	1.62	0.018	0.373
Gadd45a	growth arrest and DNA-damage-inducible 45 alpha	13197	1.62	0.003	0.202
Rasal3	RAS protein activator like 3	320484	1.62	0.012	0.328
Fosl2	fos-like antigen 2	14284	1.62	0.001	0.150
Fbxo32	F-box protein 32	67731	1.62	0.004	0.219
Arhgap27	Rho GTPase activating protein 27	544817	1.62	0.006	0.251
Dnajc18	DnaJ (Hsp40) homolog, subfamily C, member 18	76594	1.62	0.020	0.391
Adssl1	adenylosuccinate synthetase like 1	11565	1.61	0.027	0.432
Mif	macrophage migration inhibitory factor	17319	1.61	0.039	0.481
Col18a1	collagen, type XVIII, alpha 1	12822	1.60	0.014	0.340
Ptchd1	patched domain containing 1	211612	1.60	0.015	0.355
Scoc	short coiled-coil protein	56367	1.60	0.003	0.196
Ercc6	excision repair cross-complementing rodent repair deficiency, complementation group 6	319955	1.60	0.013	0.333
Fos	FBJ osteosarcoma oncogene	14281	1.60	0.007	0.258
Arl4c	ADP-ribosylation factor-like 4C	320982	1.60	0.026	0.428
Ssh2	slingshot homolog 2 (Drosophila)	237860	1.60	0.002	0.156
Creg2	cellular repressor of E1A-stimulated genes 2	263764	1.59	0.010	0.302
Slmo1	slowmo homolog 1 (Drosophila)	225655	1.59	0.020	0.399
Fat3	FAT tumor suppressor homolog 3 (Drosophila)	270120	1.59	0.018	0.375
Cacna1d	calcium channel, voltage-dependent, L type, alpha 1D subunit	12289	1.59	0.002	0.156
Klhl25	kelch-like 25 (Drosophila)	207952	1.59	0.024	0.415
Thsd1	thrombospondin, type I, domain 1	56229	1.58	0.005	0.238
Slc2a1	solute carrier family 2 (facilitated glucose transporter), member 1	20525	1.58	0.019	0.390
Cd5	CD5 antigen	12507	1.58	0.003	0.199
Mif	macrophage migration inhibitory factor	17319	1.58	0.032	0.462
Slc41a2	solute carrier family 41, member 2	338365	1.57	0.012	0.328
Pdk1	pyruvate dehydrogenase kinase, isoenzyme 1	228026	1.56	0.015	0.352
Dtx4	deltex 4 homolog (Drosophila)	207521	1.56	0.006	0.251
Mif	macrophage migration inhibitory factor	17319	1.56	0.038	0.477
Myo1d	myosin ID	338367	1.56	0.018	0.372
Slc25a25	solute carrier family 25 (mitochondrial carrier, phosphate carrier), member 25	227731	1.56	0.026	0.430
Nedd9	neural precursor cell expressed, developmentally down-regulated gene 9	18003	1.56	0.008	0.280
Chst11	carbohydrate sulfotransferase 11	58250	1.55	0.002	0.176
Mettl14	methyltransferase like 14	210529	1.55	0.009	0.290
Tm4sf19	transmembrane 4 L six family member 19	277203	1.55	0.013	0.333
Nanos1	nanos homolog 1 (Drosophila)	332397	1.55	0.020	0.391
Parvg	parvin, gamma	64099	1.55	0.017	0.368
Galnt9	UDP-N-acetyl-alpha-D-galactosamine:polypeptide N-acetylgalactosaminyltransferase 9	231605	1.55	0.026	0.430
Rmi1	RMI1, RecQ mediated genome instability 1, homolog (S. cerevisiae)	74386	1.55	0.019	0.379
Ly6a	lymphocyte antigen 6 complex, locus A	110454	1.54	0.040	0.483
Adora3	adenosine A3 receptor	11542	1.54	0.019	0.390

Maml3	mastermind like 3 (Drosophila)	433586	1.54	0.007	0.272
Rbpms	RNA binding protein gene with multiple splicing	19663	1.54	0.048	0.511
Ero1l	ERO1-like (S. cerevisiae)	50527	1.54	0.029	0.448
Raet1d	retinoic acid early transcript delta	56554	1.54	0.008	0.275
Sirpb1a	signal-regulatory protein beta 1A	320832	1.53	0.007	0.260
Gatsl3	GATS protein-like 3	71962	1.53	0.012	0.329
Micalcl	MICAL C-terminal like	70877	1.53	0.001	0.127
Npy	neuropeptide Y	109648	1.53	0.039	0.480
Fbxl7	F-box and leucine-rich repeat protein 7	448987	1.53	0.035	0.472
Eno1	enolase 1, alpha non-neuron	13806	1.53	0.032	0.462
Ssx2ip	synovial sarcoma, X breakpoint 2 interacting protein	99167	1.53	0.027	0.438
Cyp2d40	cytochrome P450, family 2, subfamily d, polypeptide 40	71754	1.53	0.016	0.359
Cebpa	CCAAT/enhancer binding protein (C/EBP), alpha	12606	1.53	0.005	0.228
B3gnt7	UDP-GlcNAc:betaGal beta-1,3-N-acetylglucosaminyltransferase 7	227327	1.53	0.014	0.343
Mfap3l	microfibrillar-associated protein 3-like	71306	1.52	0.018	0.377
Lhfpl2	lipoma HMGIC fusion partner-like 2	218454	1.52	0.013	0.333
Etl4	enhancer trap locus 4	208618	1.52	0.002	0.167
Lrpap1	low density lipoprotein receptor-related protein associated protein 1	16976	1.52	0.006	0.254
Tbc1d9	TBC1 domain family, member 9	71310	1.52	0.005	0.227
Htra4	HtrA serine peptidase 4	330723	1.52	0.005	0.236
Chst1	carbohydrate (keratan sulfate Gal-6) sulfotransferase 1	76969	1.52	0.023	0.409
Eno1	enolase 1, alpha non-neuron	13806	1.51	0.031	0.458
Col15a1	collagen, type XV, alpha 1	12819	1.51	0.005	0.227
Eno2	enolase 2, gamma neuronal	13807	1.51	0.018	0.377
Slc23a2	solute carrier family 23 (nucleobase transporters), member 2	54338	1.50	0.008	0.278
9330175E14Rik	RIKEN cDNA 9330175E14 gene	320377	1.50	0.015	0.348
Dusp9	dual specificity phosphatase 9	75590	1.50	0.010	0.299
Trim16	tripartite motif-containing 16	94092	-1.50	0.014	0.340
St18	suppression of tumorigenicity 18	240690	-1.50	0.016	0.362
Ccdc62	coiled-coil domain containing 62	208908	-1.50	0.005	0.240
Slc37a3	solute carrier family 37 (glycerol-3-phosphate transporter), member 3	72144	-1.50	0.024	0.415
Rnf213	ring finger protein 213	672511	-1.50	0.012	0.328
Serpinb6a	serine (or cysteine) peptidase inhibitor, clade B, member 6a	20719	-1.50	0.013	0.335
Rnf213	ring finger protein 213	672511	-1.50	0.010	0.298
March1	membrane-associated ring finger (C3HC4) 1	72925	-1.50	0.007	0.264
Rnf213	ring finger protein 213	672511	-1.51	0.008	0.273
Slc16a6	solute carrier family 16 (monocarboxylic acid transporters), member 6	104681	-1.51	0.023	0.410
St3gal4	ST3 beta-galactoside alpha-2,3-sialyltransferase 4	20443	-1.51	0.005	0.227
Bckdhb	branched chain ketoacid dehydrogenase E1, beta polypeptide	12040	-1.51	0.020	0.391
Tpk1	thiamine pyrophosphokinase	29807	-1.51	0.014	0.342
Layn	layilin	244864	-1.51	0.033	0.467
Rarg	retinoic acid receptor, gamma	19411	-1.51	0.040	0.485
Cdk1	cyclin-dependent kinase 1	12534	-1.52	0.001	0.145
Ahnak	AHNAK nucleoprotein (desmoyokin)	66395	-1.52	0.018	0.374
Eml6	echinoderm microtubule associated protein like 6	237711	-1.52	0.010	0.295
Enox2	ecto-NOX disulfide-thiol exchanger 2	209224	-1.52	0.003	0.188

Nat8l	N-acetyltransferase 8-like	269642	-1.52	0.023	0.415
H2-T24	histocompatibility 2, T region locus 24	15042	-1.52	<0.001	0.097
Lphn1	latrophilin 1	330814	-1.52	0.004	0.206
Il2rg	interleukin 2 receptor, gamma chain	16186	-1.52	0.020	0.396
Loxl3	lysyl oxidase-like 3	16950	-1.53	0.038	0.477
Stom	stomatin	13830	-1.53	0.048	0.510
Casp1	caspase 1	12362	-1.53	0.028	0.445
Gm10134	predicted gene 10134	100038637	-1.53	0.005	0.228
Rnf157	ring finger protein 157	217340	-1.53	0.011	0.321
Sdc4	syndecan 4	20971	-1.53	0.012	0.326
Dclre1c	DNA cross-link repair 1C, PSO2 homolog (S. cerevisiae)	227525	-1.53	0.003	0.197
Susd1	sushi domain containing 1	634731	-1.53	0.023	0.409
Fhit	fragile histidine triad gene	14198	-1.53	0.021	0.401
Tcfap4	transcription factor AP4	83383	-1.53	0.008	0.276
Dctd	dCMP deaminase	320685	-1.53	0.033	0.467
Aldh1l1	aldehyde dehydrogenase 1 family, member L1	107747	-1.53	0.007	0.263
Adk	adenosine kinase	11534	-1.54	0.006	0.243
Abcb1b	ATP-binding cassette, sub-family B (MDR/TAP), member 1B	18669	-1.54	0.045	0.503
Emilin2	elastin microfibril interfacer 2	246707	-1.54	0.012	0.328
Slfn5	schlafen 5	327978	-1.54	0.034	0.469
Plagl2	pleiomorphic adenoma gene-like 2	54711	-1.54	0.008	0.280
St7	suppression of tumorigenicity 7	64213	-1.54	0.003	0.202
Maoa	monoamine oxidase A	17161	-1.54	0.021	0.401
Nme4	non-metastatic cells 4, protein expressed in	56520	-1.54	0.020	0.399
Fcgrt	Fc receptor, IgG, alpha chain transporter	14132	-1.54	0.034	0.469
Ksr1	kinase suppressor of ras 1	16706	-1.55	0.012	0.330
Mir219-1	microRNA 219-1	723823	-1.55	0.039	0.481
Tns1	tensin 1	21961	-1.55	0.047	0.508
Pde4b	phosphodiesterase 4B, cAMP specific	18578	-1.55	0.001	0.131
2210404J11Rik	RIKEN cDNA 2210404J11 gene	381062	-1.55	0.010	0.302
Lpl	lipoprotein lipase	16956	-1.55	0.039	0.480
Lpar5	lysophosphatidic acid receptor 5	381810	-1.55	0.010	0.306
Dse	dermatan sulfate epimerase	212898	-1.55	0.017	0.369
Atp6v0a1	ATPase, H+ transporting, lysosomal V0 subunit A1	11975	-1.55	0.009	0.292
Vopp1	vesicular, overexpressed in cancer, prosurvival protein 1	232023	-1.55	0.006	0.250
Rnf213	ring finger protein 213	672511	-1.56	0.025	0.422
Gm6033	predicted gene 6033	547328	-1.56	0.025	0.421
Klra3	killer cell lectin-like receptor, subfamily A, member 3	16634	-1.56	0.035	0.471
Large	like-glycosyltransferase	16795	-1.56	0.037	0.473
Gmfg	glia maturation factor, gamma	63986	-1.56	0.009	0.291
Inpp4a	inositol polyphosphate-4-phosphatase, type I	269180	-1.56	0.016	0.360
Klraq1	KLRAQ motif containing 1	73825	-1.56	0.007	0.270
Nagk	N-acetylglucosamine kinase	56174	-1.56	0.007	0.257
Aph1b	anterior pharynx defective 1b homolog (C. elegans)	208117	-1.56	0.002	0.171
2810055G20Rik	RIKEN cDNA 2810055G20 gene	77994	-1.56	0.028	0.444
Aph1c	anterior pharynx defective 1c homolog (C. elegans)	68318	-1.56	0.006	0.245

Zfp90	zinc finger protein 90	22751	-1.57	0.012	0.328
Ttc32	tetratricopeptide repeat domain 32	75516	-1.57	0.016	0.360
Gstm4	glutathione S-transferase, mu 4	14865	-1.57	0.013	0.336
Nr1h3	nuclear receptor subfamily 1, group H, member 3	22259	-1.57	0.022	0.406
Ehd4	EH-domain containing 4	98878	-1.57	0.019	0.388
Ankrd33b	ankyrin repeat domain 33B	67434	-1.57	0.003	0.193
Scamp5	secretory carrier membrane protein 5	56807	-1.57	0.030	0.454
Pmaip1	phorbol-12-myristate-13-acetate-induced protein 1	58801	-1.58	0.024	0.419
Foxred2	FAD-dependent oxidoreductase domain containing 2	239554	-1.58	0.005	0.240
Shpk	sedoheptulokinase	74637	-1.58	0.038	0.477
Gdpd1	glycerophosphodiester phosphodiesterase domain containing 1	66569	-1.58	0.024	0.420
Wdfy4	WD repeat and FYVE domain containing 4	545030	-1.58	0.017	0.369
Arhgef37	Rho guanine nucleotide exchange factor (GEF) 37	328967	-1.58	0.021	0.401
Hk3	hexokinase 3	212032	-1.58	0.030	0.455
Fam129a	family with sequence similarity 129, member A	63913	-1.58	0.004	0.221
Tor3a	torsin family 3, member A	30935	-1.58	0.023	0.412
Mmp13	matrix metallopeptidase 13	17386	-1.58	0.017	0.366
2310047M10Rik	RIKEN cDNA 2310047M10 gene	71923	-1.59	0.011	0.311
Metrnl	meteorin, glial cell differentiation regulator-like	210029	-1.59	0.037	0.474
Samd9l	sterile alpha motif domain containing 9-like	209086	-1.59	0.022	0.406
Tmem106a	transmembrane protein 106A	217203	-1.59	0.015	0.352
Hck	hemopoietic cell kinase	15162	-1.59	0.033	0.467
Xaf1	XIAP associated factor 1	327959	-1.59	0.038	0.477
Nfkbia	nuclear factor of kappa light polypeptide gene enhancer in B-cells inhibitor, alpha	18035	-1.59	0.009	0.285
Mpa2l	macrophage activation 2 like	100702	-1.60	0.012	0.328
Abcc5	ATP-binding cassette, sub-family C (CFTR/MRP), member 5	27416	-1.60	0.014	0.338
Mir99a	microRNA 99a	387229	-1.60	0.031	0.457
Hgsnat	heparan-alpha-glucosaminide N-acetyltransferase	52120	-1.60	0.013	0.335
St8sia4	ST8 alpha-N-acetyl-neuraminide alpha-2,8-sialyltransferase 4	20452	-1.60	0.037	0.473
Tnfaip2	tumor necrosis factor, alpha-induced protein 2	21928	-1.60	0.007	0.257
Ly9	lymphocyte antigen 9	17085	-1.60	0.014	0.338
Fut7	fucosyltransferase 7	14347	-1.60	0.043	0.496
Ddx60	DEAD (Asp-Glu-Ala-Asp) box polypeptide 60	234311	-1.61	0.003	0.191
Stap1	signal transducing adaptor family member 1	56792	-1.61	0.021	0.401
Ephx1	epoxide hydrolase 1, microsomal	13849	-1.61	0.022	0.404
2410066E13Rik	RIKEN cDNA 2410066E13 gene	68235	-1.61	0.003	0.192
Gm12185	predicted gene 12185	620913	-1.61	0.040	0.486
Clcn5	chloride channel 5	12728	-1.61	0.046	0.505
Rnf213	ring finger protein 213	672511	-1.62	0.002	0.176
Fam114a1	family with sequence similarity 114, member A1	68303	-1.62	0.006	0.253
Gng2	guanine nucleotide binding protein (G protein), gamma 2	14702	-1.62	0.022	0.406
Tm6sf1	transmembrane 6 superfamily member 1	107769	-1.62	0.018	0.372
Oasl2	2'-5' oligoadenylate synthetase-like 2	23962	-1.62	0.028	0.444
Arhgap4	Rho GTPase activating protein 4	171207	-1.62	0.020	0.391
N4bp2l1	NEDD4 binding protein 2-like 1	100637	-1.62	0.002	0.173

Tfb1m	transcription factor B1, mitochondrial	224481	-1.62	0.009	0.294
Dcxr	dicarbonyl L-xylulose reductase	67880	-1.62	0.043	0.497
Entpd1	ectonucleoside triphosphate diphosphohydrolase 1	12495	-1.63	0.005	0.232
Depdc6	DEP domain containing 6	97998	-1.63	0.007	0.270
Sdr42e1	short chain dehydrogenase/reductase family 42E, member 1	74032	-1.63	0.013	0.333
Pik3r6	phosphoinositide-3-kinase, regulatory subunit 6	104709	-1.63	0.028	0.442
Cables1	CDK5 and Abl enzyme substrate 1	63955	-1.64	0.003	0.202
Gstm7	glutathione S-transferase, mu 7	68312	-1.64	0.046	0.504
Tle3	transducin-like enhancer of split 3, homolog of Drosophila E(spl)	21887	-1.64	0.030	0.453
Plekho2	pleckstrin homology domain containing, family O member 2	102595	-1.64	0.021	0.400
Slc45a4	solute carrier family 45, member 4	106068	-1.65	0.009	0.290
Fchsd2	FCH and double SH3 domains 2	207278	-1.65	0.016	0.362
Paox	polyamine oxidase (exo-N4-amino)	212503	-1.65	0.011	0.320
Ap2a2	adaptor protein complex AP-2, alpha 2 subunit	11772	-1.65	0.022	0.405
Athl1	ATH1, acid trehalase-like 1 (yeast)	212974	-1.65	0.016	0.361
Cd40	CD40 antigen	21939	-1.65	0.030	0.454
Rac2	RAS-related C3 botulinum substrate 2	19354	-1.65	0.034	0.467
Hip1	huntingtin interacting protein 1	215114	-1.65	0.016	0.359
Nfkbie	nuclear factor of kappa light polypeptide gene enhancer in B-cells inhibitor, epsilon	18037	-1.65	0.006	0.254
Mink1	misshapen-like kinase 1 (zebrafish)	50932	-1.65	0.011	0.320
1190002N15Rik	RIKEN cDNA 1190002N15 gene	68861	-1.65	0.005	0.227
Nfxl1	nuclear transcription factor, X-box binding-like 1	100978	-1.66	0.015	0.348
Klf7	Kruppel-like factor 7 (ubiquitous)	93691	-1.66	0.001	0.139
Lrmp	lymphoid-restricted membrane protein	16970	-1.66	0.003	0.189
Rnf213	ring finger protein 213	672511	-1.66	0.018	0.372
Slc16a9	solute carrier family 16 (monocarboxylic acid transporters), member 9	66859	-1.66	0.016	0.358
Ccl3	chemokine (C-C motif) ligand 3	20302	-1.67	0.012	0.328
Ptplad2	protein tyrosine phosphatase-like A domain containing 2	66775	-1.67	0.005	0.227
Nfxl1	nuclear transcription factor, X-box binding-like 1	100978	-1.67	0.027	0.435
6430548M08Rik	RIKEN cDNA 6430548M08 gene	234797	-1.67	0.031	0.461
Fam49a	family with sequence similarity 49, member A	76820	-1.67	0.009	0.290
Slco3a1	solute carrier organic anion transporter family, member 3a1	108116	-1.67	0.022	0.406
Bri3	brain protein I3	55950	-1.67	0.003	0.187
Il18	interleukin 18	16173	-1.67	0.046	0.508
Nup62	nucleoporin 62	18226	-1.67	0.026	0.426
Adcy3	adenylate cyclase 3	104111	-1.68	0.026	0.430
Fbxw10	F-box and WD-40 domain protein 10	213980	-1.68	0.013	0.336
Gbgt1	globoside alpha-1,3-N-acetylgalactosaminyltransferase 1	227671	-1.68	0.015	0.350
Anxa6	annexin A6	11749	-1.68	0.021	0.401
Phf17	PHD finger protein 17	269424	-1.68	0.032	0.462
Il15	interleukin 15	16168	-1.69	0.011	0.309
Ifi205	interferon activated gene 205	226695	-1.69	0.041	0.489
Snx8	sorting nexin 8	231834	-1.69	0.008	0.280
Nfxl1	nuclear transcription factor, X-box binding-like 1	100978	-1.69	0.006	0.254
Ptk2	PTK2 protein tyrosine kinase 2	14083	-1.69	0.018	0.377

Sncaip	synuclein, alpha interacting protein (synphilin)	67847	-1.69	0.004	0.220
Ust	uronyl-2-sulfotransferase	338362	-1.70	0.013	0.336
Gmfg	glia maturation factor, gamma	63986	-1.70	0.004	0.223
Rassf4	Ras association (RalGDS/AF-6) domain family member 4	213391	-1.70	0.004	0.221
Ets2	E26 avian leukemia oncogene 2, 3' domain	23872	-1.70	0.016	0.360
H2-M2	histocompatibility 2, M region locus 2	14990	-1.71	0.048	0.512
Irf1	interferon regulatory factor 1	16362	-1.72	0.009	0.281
H2-K2	histocompatibility 2, K region locus 2	630499	-1.72	0.020	0.395
Aim1	absent in melanoma 1	11630	-1.73	0.018	0.376
Gstm1	glutathione S-transferase, mu 1	14862	-1.73	0.028	0.440
P2ry14	purinergic receptor P2Y, G-protein coupled, 14	140795	-1.73	0.003	0.181
Zbtb8a	zinc finger and BTB domain containing 8a	73680	-1.73	0.014	0.341
Mamld1	mastermind-like domain containing 1	333639	-1.74	0.008	0.273
Pml	promyelocytic leukemia	18854	-1.74	0.010	0.298
Zfp703	zinc finger protein 703	353310	-1.74	0.027	0.432
Blvra	biliverdin reductase A	109778	-1.74	0.007	0.264
Tmem38b	transmembrane protein 38B	52076	-1.74	0.002	0.156
Vav3	vav 3 oncogene	57257	-1.75	0.010	0.298
Tbc1d2b	TBC1 domain family, member 2B	67016	-1.75	0.007	0.262
Tecpr1	tectonin beta-propeller repeat containing 1	70381	-1.75	0.020	0.396
March3	membrane-associated ring finger (C3HC4) 3	320253	-1.75	0.010	0.304
Evl	Ena-vasodilator stimulated phosphoprotein	14026	-1.76	0.019	0.379
Gab3	growth factor receptor bound protein 2-associated protein 3	210710	-1.76	0.014	0.341
Pstpip2	proline-serine-threonine phosphatase-interacting protein 2	19201	-1.77	0.013	0.331
Plekho2	pleckstrin homology domain containing, family O member 2	102595	-1.77	<0.001	0.069
Stk17b	serine/threonine kinase 17b (apoptosis-inducing)	98267	-1.77	0.005	0.228
Adrbk2	adrenergic receptor kinase, beta 2	320129	-1.78	0.001	0.150
Atf5	activating transcription factor 5	107503	-1.78	0.004	0.217
Hpse	heparanase	15442	-1.78	0.002	0.156
Marveld1	MARVEL (membrane-associating) domain containing 1	277010	-1.79	0.012	0.328
Tgtp1	T-cell specific GTPase 1	21822	-1.79	0.011	0.321
Ralgds	ral guanine nucleotide dissociation stimulator	19730	-1.80	0.001	0.140
Aurkb	aurora kinase B	20877	-1.80	0.005	0.227
Tgtp1	T-cell specific GTPase 1	21822	-1.80	0.008	0.274
D630023F18Rik	RIKEN cDNA D630023F18 gene	98303	-1.80	0.004	0.216
Tmem154	transmembrane protein 154	320782	-1.81	0.017	0.368
Tcf7l2	transcription factor 7-like 2, T-cell specific, HMG-box	21416	-1.81	0.012	0.325
Ptafr	platelet-activating factor receptor	19204	-1.81	0.014	0.343
Pgap1	post-GPI attachment to proteins 1	241062	-1.81	0.012	0.328
1700009P17Rik	RIKEN cDNA 1700009P17 gene	75472	-1.82	0.028	0.444
Gm6377	predicted gene 6377	622976	-1.82	0.010	0.298
Fah	fumarylacetoacetate hydrolase	14085	-1.82	0.011	0.316
4930444A02Rik	RIKEN cDNA 4930444A02 gene	74653	-1.82	0.016	0.359
Prrg1	proline rich Gla (G-carboxyglutamic acid) 1	546336	-1.82	0.006	0.255
Gm9949	predicted gene 9949	225609	-1.83	0.015	0.349

Gstm3	glutathione S-transferase, mu 3	14864	-1.83	0.013	0.335
Ifi44	interferon-induced protein 44	99899	-1.83	0.003	0.204
Cpeb3	cytoplasmic polyadenylation element binding protein 3	208922	-1.83	0.011	0.320
Wdfy2	WD repeat and FYVE domain containing 2	268752	-1.83	0.007	0.261
Mcoln2	mucolipin 2	68279	-1.83	0.003	0.187
Tcte3	t-complex-associated testis expressed 3	21647	-1.83	0.008	0.273
Pira1	paired-Ig-like receptor A1	18722	-1.83	0.019	0.380
Ggt5	gamma-glutamyltransferase 5	23887	-1.84	0.002	0.156
Med22	mediator complex subunit 22	20933	-1.84	0.022	0.404
Cd74	CD74 antigen (invariant polypeptide of major histocompatibility complex, class II antigen-associated)	16149	-1.84	0.041	0.487
Id3	inhibitor of DNA binding 3	15903	-1.85	0.008	0.280
Hivep2	human immunodeficiency virus type I enhancer binding protein 2	15273	-1.85	0.006	0.256
Cd4	CD4 antigen	12504	-1.85	0.002	0.171
Ifitm1	interferon induced transmembrane protein 1	68713	-1.85	0.033	0.466
Pram1	PML-RAR alpha-regulated adaptor molecule 1	378460	-1.86	0.030	0.456
Samd4	sterile alpha motif domain containing 4	74480	-1.86	0.006	0.253
Frmd4b	FERM domain containing 4B	232288	-1.86	0.013	0.333
Dbp	D site albumin promoter binding protein	13170	-1.86	0.005	0.228
Anpep	alanyl (membrane) aminopeptidase	16790	-1.86	0.018	0.372
Lilra6	leukocyte immunoglobulin-like receptor, subfamily A (with TM domain), member 6	18726	-1.86	0.037	0.473
Adrb2	adrenergic receptor, beta 2	11555	-1.87	0.008	0.280
Lrrc33	leucine rich repeat containing 33	224109	-1.87	0.005	0.232
Npl	N-acetylneuraminate pyruvate lyase	74091	-1.87	0.003	0.202
Casp4	caspase 4, apoptosis-related cysteine peptidase	12363	-1.87	0.005	0.236
F630028O10Rik	RIKEN cDNA F630028O10 gene	100038363	-1.87	0.008	0.280
BC013712	cDNA sequence BC013712	230787	-1.87	0.005	0.227
Tnfsf15	tumor necrosis factor (ligand) superfamily, member 15	326623	-1.88	0.012	0.327
Cd300ld	CD300 molecule-like family member d	217305	-1.88	0.009	0.286
Treml4	triggering receptor expressed on myeloid cells-like 4	224840	-1.88	0.036	0.473
Pira11	paired-Ig-like receptor A11	18724	-1.88	0.011	0.313
Tlr6	toll-like receptor 6	21899	-1.88	0.010	0.304
Cyp27a1	cytochrome P450, family 27, subfamily a, polypeptide 1	104086	-1.88	0.003	0.189
Ifitm2	interferon induced transmembrane protein 2	80876	-1.89	0.043	0.497
9030625A04Rik	RIKEN cDNA 9030625A04 gene	210808	-1.90	<0.001	0.078
Lilrb3	leukocyte immunoglobulin-like receptor, subfamily B (with TM and ITIM domains), member 3	18733	-1.90	0.015	0.350
Esr1	estrogen receptor 1 (alpha)	13982	-1.91	0.004	0.222
Itga4	integrin alpha 4	16401	-1.91	0.002	0.156
Rnasel	ribonuclease L (2', 5'-oligoisoadenylate synthetase-dependent)	24014	-1.91	0.005	0.227
Fas	Fas (TNF receptor superfamily member 6)	14102	-1.91	0.011	0.313
Ncoa4	nuclear receptor coactivator 4	27057	-1.91	0.004	0.222
Tcte3	t-complex-associated testis expressed 3	21647	-1.92	0.005	0.227
Ncoa4	nuclear receptor coactivator 4	27057	-1.92	0.004	0.220
Pgap1	post-GPI attachment to proteins 1	241062	-1.92	0.038	0.478
Scn1b	sodium channel, voltage-gated, type I, beta	20266	-1.93	0.002	0.161
Vsig4	V-set and immunoglobulin domain containing 4	278180	-1.94	0.014	0.340

Lpin1	lipin 1	14245	-1.94	0.003	0.184
Tmem176b	transmembrane protein 176B	65963	-1.94	0.007	0.262
Thbs1	thrombospondin 1	21825	-1.94	0.016	0.362
Zfp36l1	zinc finger protein 36, C3H type-like 1	12192	-1.94	0.005	0.228
Arl5c	ADP-ribosylation factor-like 5C	217151	-1.95	0.004	0.216
Zkscan17	zinc finger with KRAB and SCAN domains 17	268417	-1.95	0.020	0.396
Cd200r1	CD200 receptor 1	57781	-1.96	0.004	0.221
Ifitm2	interferon induced transmembrane protein 2	80876	-1.96	0.041	0.487
Cd5l	CD5 antigen-like	11801	-1.96	0.013	0.333
Nlrp3	NLR family, pyrin domain containing 3	216799	-1.96	0.008	0.273
Mmp12	matrix metallopeptidase 12	17381	-1.96	0.009	0.291
Lrrc25	leucine rich repeat containing 25	211228	-1.97	0.013	0.333
Mir146	microRNA 146	387164	-1.97	0.018	0.378
Igfbp4	insulin-like growth factor binding protein 4	16010	-1.98	0.015	0.352
Pdxk-ps	pyridoxal (pyridoxine, vitamin B6) kinase, pseudogene	435518	-1.98	0.008	0.273
Cd300a	CD300A antigen	217303	-2.00	0.006	0.240
Slc46a3	solute carrier family 46, member 3	71706	-2.01	0.002	0.156
Ddx58	DEAD (Asp-Glu-Ala-Asp) box polypeptide 58	230073	-2.01	0.001	0.147
Rnase6	ribonuclease, RNase A family, 6	78416	-2.01	0.019	0.384
Rgs18	regulator of G-protein signaling 18	64214	-2.01	0.006	0.254
Ifi47	interferon gamma inducible protein 47	15953	-2.02	0.003	0.187
Aoah	acyloxyacyl hydrolase	27052	-2.02	0.001	0.152
Clec4e	C-type lectin domain family 4, member e	56619	-2.02	0.002	0.167
Fgd4	FYVE, RhoGEF and PH domain containing 4	224014	-2.03	0.004	0.213
Cd83	CD83 antigen	12522	-2.03	0.001	0.138
Tmem176a	transmembrane protein 176A	66058	-2.03	0.003	0.202
St3gal5	ST3 beta-galactoside alpha-2,3-sialyltransferase 5	20454	-2.04	<0.001	0.073
Ifi203	interferon activated gene 203	15950	-2.05	0.004	0.217
Tmem195	transmembrane protein 195	319660	-2.07	0.005	0.236
Ifitm3	interferon induced transmembrane protein 3	66141	-2.08	0.012	0.328
2210404J11Rik	RIKEN cDNA 2210404J11 gene	381062	-2.08	<0.001	0.049
Ifitm2	interferon induced transmembrane protein 2	80876	-2.09	0.017	0.372
Abca9	ATP-binding cassette, sub-family A (ABC1), member 9	217262	-2.10	0.007	0.273
Kctd12	potassium channel tetramerisation domain containing 12	239217	-2.10	<0.001	0.073
Lbr	lamin B receptor	98386	-2.10	0.002	0.156
Rilpl2	Rab interacting lysosomal protein-like 2	80291	-2.10	0.010	0.295
Gstp1	glutathione S-transferase, pi 1	14870	-2.11	0.046	0.508
Panx1	pannexin 1	55991	-2.11	0.006	0.254
Sult1a1	sulfotransferase family 1A, phenol-preferring, member 1	20887	-2.11	0.038	0.478
Ptpn22	protein tyrosine phosphatase, non-receptor type 22 (lymphoid)	19260	-2.11	0.003	0.196
Mlkl	mixed lineage kinase domain-like	74568	-2.12	0.003	0.196
Pf4	platelet factor 4	56744	-2.12	0.008	0.275
Gas6	growth arrest specific 6	14456	-2.13	0.009	0.290
Cd93	CD93 antigen	17064	-2.13	0.002	0.162
Oas3	2'-5' oligoadenylate synthetase 3	246727	-2.13	0.004	0.205

Usp18	ubiquitin specific peptidase 18	24110	-2.13	0.015	0.350
Cebpd	CCAAT/enhancer binding protein (C/EBP), delta	12609	-2.16	0.001	0.127
Ikbke	inhibitor of kappaB kinase epsilon	56489	-2.16	0.002	0.167
Clec7a	C-type lectin domain family 7, member a	56644	-2.16	0.004	0.216
Mki67	antigen identified by monoclonal antibody Ki 67	17345	-2.17	<0.001	0.064
Fgd2	FYVE, RhoGEF and PH domain containing 2	26382	-2.17	0.003	0.188
BC055004	cDNA sequence BC055004	381680	-2.17	0.006	0.252
Ifitm1	interferon induced transmembrane protein 1	68713	-2.17	0.003	0.191
2210404J11Rik	RIKEN cDNA 2210404J11 gene	381062	-2.17	<0.001	0.048
Kif23	kinesin family member 23	71819	-2.18	<0.001	0.073
Oas2	2'-5' oligoadenylate synthetase 2	246728	-2.18	0.023	0.409
Cd97	CD97 antigen	26364	-2.20	0.007	0.258
Sorl1	sortilin-related receptor, LDLR class A repeats-containing	20660	-2.22	0.001	0.098
BC048502	cDNA sequence BC048502	223927	-2.22	0.001	0.112
Epsti1	epithelial stromal interaction 1 (breast)	108670	-2.23	0.002	0.156
Tmem8	transmembrane protein 8 (five membrane-spanning domains)	60455	-2.26	0.011	0.307
Fcgr4	Fc receptor, IgG, low affinity IV	246256	-2.27	0.003	0.202
Pstpip2	proline-serine-threonine phosphatase-interacting protein 2	19201	-2.27	0.037	0.475
Cd36	CD36 antigen	12491	-2.30	0.005	0.240
Pdxk	pyridoxal (pyridoxine, vitamin B6) kinase	216134	-2.30	0.007	0.269
Cnrip1	cannabinoid receptor interacting protein 1	380686	-2.31	0.001	0.149
Slc16a7	solute carrier family 16 (monocarboxylic acid transporters), member 7	20503	-2.31	<0.001	0.087
Cd69	CD69 antigen	12515	-2.32	0.007	0.259
Gm11711	predicted gene 11711	100043125	-2.33	0.007	0.257
Itsn1	intersectin 1 (SH3 domain protein 1A)	16443	-2.34	0.002	0.167
P2ry13	purinergic receptor P2Y, G-protein coupled 13	74191	-2.35	0.001	0.115
Ext1	exostoses (multiple) 1	14042	-2.35	0.003	0.196
Dok2	docking protein 2	13449	-2.35	0.009	0.290
Fcrl1	Fc receptor-like 1	229499	-2.36	0.008	0.276
Siglece	sialic acid binding Ig-like lectin E	83382	-2.36	0.001	0.133
Gm11711	predicted gene 11711	100043125	-2.37	0.008	0.273
Filip1l	filamin A interacting protein 1-like	78749	-2.37	0.001	0.137
Clec2j	C-type lectin domain family 2, member J	677440	-2.37	0.002	0.167
Ahrr	aryl-hydrocarbon receptor repressor	11624	-2.37	0.006	0.255
Cxcl2	chemokine (C-X-C motif) ligand 2	20310	-2.38	<0.001	0.064
2810405K02Rik	RIKEN cDNA 2810405K02 gene	66469	-2.38	0.006	0.245
Rnd3	Rho family GTPase 3	74194	-2.38	0.002	0.178
Marcksl1	MARCKS-like 1	17357	-2.40	0.003	0.187
Ccl4	chemokine (C-C motif) ligand 4	20303	-2.40	0.006	0.242
Pdlim4	PDZ and LIM domain 4	30794	-2.41	0.007	0.260
Herpud1	homocysteine-inducible, endoplasmic reticulum stress-inducible, ubiquitin-like domain member 1	64209	-2.41	0.002	0.156
Pot1b	protection of telomeres 1B	72836	-2.42	<0.001	0.069
Il1b	interleukin 1 beta	16176	-2.46	0.001	0.141
Arhgap26	Rho GTPase activating protein 26	71302	-2.46	0.001	0.120
Dmwd	dystrophia myotonica-containing WD repeat motif	13401	-2.46	0.001	0.120

Cd80	CD80 antigen	12519	-2.46	0.001	0.146
Ppfibp2	PTPRF interacting protein, binding protein 2 (liprin beta 2)	19024	-2.47	0.002	0.158
Rasgrp4	RAS guanyl releasing protein 4	233046	-2.47	0.012	0.328
Niacr1	niacin receptor 1	80885	-2.49	0.005	0.227
Engase	endo-beta-N-acetylglucosaminidase	217364	-2.49	0.001	0.112
Rhobtb1	Rho-related BTB domain containing 1	69288	-2.52	<0.001	0.073
Gprc5c	G protein-coupled receptor, family C, group 5, member C	70355	-2.52	0.002	0.156
Akr1b8	aldo-keto reductase family 1, member B8	14187	-2.53	0.006	0.243
Tcfec	transcription factor EC	21426	-2.54	0.002	0.156
EG214403	predicted gene, EG214403	214403	-2.54	0.001	0.121
Gpr162	G protein-coupled receptor 162	14788	-2.55	0.002	0.167
Pparg	peroxisome proliferator activated receptor gamma	19016	-2.55	0.004	0.207
Ms4a4c	membrane-spanning 4-domains, subfamily A, member 4C	64380	-2.57	0.009	0.289
Nod1	nucleotide-binding oligomerization domain containing 1	107607	-2.57	0.001	0.109
Slc40a1	solute carrier family 40 (iron-regulated transporter), member 1	53945	-2.58	0.001	0.141
Gm12250	predicted gene 12250	631323	-2.60	0.004	0.214
Clec2d	C-type lectin domain family 2, member d	93694	-2.61	0.004	0.208
Gprc5c	G protein-coupled receptor, family C, group 5, member C	70355	-2.63	0.001	0.140
2210404J11Rik	RIKEN cDNA 2210404J11 gene	381062	-2.64	<0.001	0.073
Gbp6	guanylate binding protein 6	229900	-2.64	0.004	0.205
Pde7b	phosphodiesterase 7B	29863	-2.65	<0.001	0.075
Tlr1	toll-like receptor 1	21897	-2.66	0.002	0.156
Cfh	complement component factor h	12628	-2.67	0.001	0.112
Raph1	Ras association (RalGDS/AF-6) and pleckstrin homology domains 1	77300	-2.67	0.004	0.224
Cd80	CD80 antigen	12519	-2.67	0.007	0.259
Ltbp2	latent transforming growth factor beta binding protein 2	16997	-2.69	0.002	0.174
Emb	embigin	13723	-2.71	0.004	0.219
Irf7	interferon regulatory factor 7	54123	-2.72	0.003	0.202
Il12rb2	interleukin 12 receptor, beta 2	16162	-2.74	0.002	0.175
Fam26f	family with sequence similarity 26, member F	215900	-2.74	<0.001	0.073
Cfhr3	complement factor H-related 3	624286	-2.75	0.001	0.108
Kctd12b	potassium channel tetramerisation domain containing 12b	207474	-2.77	0.003	0.197
Ahr	aryl-hydrocarbon receptor	11622	-2.78	0.001	0.150
Sema6d	sema domain, transmembrane domain (TM), and cytoplasmic domain, (semaphorin) 6D	214968	-2.78	0.003	0.189
Rgl1	ral guanine nucleotide dissociation stimulator,-like 1	19731	-2.80	0.005	0.236
Tfrc	transferrin receptor	22042	-2.82	0.022	0.406
Clec2g	C-type lectin domain family 2, member g	70809	-2.84	0.009	0.281
Maf	avian musculoaponeurotic fibrosarcoma (v-maf) AS42 oncogene homolog	17132	-2.92	0.006	0.240
Pilra	paired immunoglobin-like type 2 receptor alpha	231805	-2.93	0.002	0.156
Gbp9	guanylate-binding protein 9	236573	-2.95	0.007	0.269
Gm10419	predicted gene 10419	100038441	-2.95	0.022	0.406
Marco	macrophage receptor with collagenous structure	17167	-2.96	<0.001	0.073
Rtp4	receptor transporter protein 4	67775	-2.98	0.003	0.188
Gas7	growth arrest specific 7	14457	-3.01	0.005	0.227
Fam198b	family with sequence similarity 198, member B	68659	-3.01	0.003	0.200
Slamf8	SLAM family member 8	74748	-3.17	0.002	0.157

Tnfrsf14	tumor necrosis factor receptor superfamily, member 14 (herpesvirus entry mediator)	230979	-3.17	<0.001	0.082
A530064D06Rik	RIKEN cDNA A530064D06 gene	328830	-3.20	0.002	0.167
Clec4b2	C-type lectin domain family 4, member b2	381809	-3.23	<0.001	0.073
B430306N03Rik	RIKEN cDNA B430306N03 gene	320148	-3.24	0.003	0.197
Cmpk2	cytidine monophosphate (UMP-CMP) kinase 2, mitochondrial	22169	-3.30	0.002	0.156
Pyhin1	pyrin and HIN domain family, member 1	236312	-3.39	0.003	0.181
Fpr3	formyl peptide receptor 3	14294	-3.45	<0.001	0.097
Lst1	leukocyte specific transcript 1	16988	-3.46	<0.001	0.097
Gbp2	guanylate binding protein 2	14469	-3.48	<0.001	0.031
Spic	Spi-C transcription factor (Spi-1/PU.1 related)	20728	-3.60	0.006	0.247
Cdc42ep2	CDC42 effector protein (Rho GTPase binding) 2	104252	-3.60	<0.001	0.075
Slc9a9	solute carrier family 9 (sodium/hydrogen exchanger), member 9	331004	-3.62	<0.001	0.073
Ccl9	chemokine (C-C motif) ligand 9	20308	-3.75	0.004	0.222
Ebi3	Epstein-Barr virus induced gene 3	50498	-3.80	0.001	0.149
Bank1	B-cell scaffold protein with ankyrin repeats 1	242248	-4.08	<0.001	0.048
Ifitm6	interferon induced transmembrane protein 6	213002	-4.27	0.013	0.330
Ednrb	endothelin receptor type B	13618	-4.30	0.001	0.132
Fpr1	formyl peptide receptor 1	14293	-4.32	<0.001	0.097
Cfp	complement factor properdin	18636	-4.36	0.002	0.167
Ifit3	interferon-induced protein with tetratricopeptide repeats 3	15959	-4.41	<0.001	0.073
Clec4n	C-type lectin domain family 4, member n	56620	-4.62	0.001	0.144
Rsad2	radical S-adenosyl methionine domain containing 2	58185	-4.79	<0.001	0.073
Dmpk	dystrophia myotonica-protein kinase	13400	-5.01	0.002	0.156
Clec10a	C-type lectin domain family 10, member A	17312	-5.02	0.001	0.140
Cd28	CD28 antigen	12487	-5.04	0.003	0.187
Syn1	synapsin I	20964	-5.09	0.001	0.106
Gdf15	growth differentiation factor 15	23886	-5.14	0.002	0.165
Pilrb2	paired immunoglobin-like type 2 receptor beta 2	545812	-5.39	0.006	0.252
Pilrb1	paired immunoglobin-like type 2 receptor beta 1	170741	-5.47	0.003	0.185
Cxcl10	chemokine (C-X-C motif) ligand 10	15945	-5.78	0.003	0.185
Ifit1	interferon-induced protein with tetratricopeptide repeats 1	15957	-5.81	<0.001	0.096
Clec4a2	C-type lectin domain family 4, member a2	26888	-6.14	<0.001	0.082
Ifit2	interferon-induced protein with tetratricopeptide repeats 2	15958	-6.29	<0.001	0.073
Ccl6	chemokine (C-C motif) ligand 6	20305	-6.33	<0.001	0.096
Clec4a1	C-type lectin domain family 4, member a1	269799	-8.19	<0.001	0.023
Clec4a3	C-type lectin domain family 4, member a3	73149	-9.21	0.001	0.101
Mrc1	mannose receptor, C type 1	17533	-11.77	0.001	0.137
Emr4	EGF-like module containing, mucin-like, hormone receptor-like sequence 4	52614	-13.28	0.005	0.233

Supplementary Table 4. Significantly regulated Gene Ontology (GO) classes, as determined by ErmineJ analysis on gene array data.

GO class	GO ID	# Genes	Raw Score	P-value
cellular response to interferon-alpha	GO:0035457	6	2.36534771	3.10E-10
G-protein coupled receptor internalization	GO:0002031	5	2.26684427	4.03E-09
positive regulation of monocyte chemotaxis	GO:0090026	5	2.09796501	6.30E-03
regulation of response to reactive oxygen species	GO:1901031	5	2.0473355	8.58E-03
complement activation, alternative pathway	GO:0006957	5	2.00538188	1.11E-02
regulation of monocyte chemotaxis	GO:0090025	6	1.96866805	1.24E-02
adhesion to other organism involved in symbiotic interaction	GO:0051825	9	1.95449853	1.83E-10
adhesion to symbiont	GO:0051856	8	1.9443492	3.80E-03
microtubule polymerization	GO:0046785	5	1.91834601	2.17E-02
negative regulation of endothelial cell apoptotic process	GO:2000352	8	1.90162606	3.67E-03
urea cycle	GO:0000050	5	1.89589233	2.54E-02
urea metabolic process	GO:0019627	5	1.89589233	2.52E-02
nitrogen cycle metabolic process	GO:0071941	5	1.89589233	2.52E-02
negative regulation of vasoconstriction	GO:0045906	5	1.88974156	2.69E-02
spindle midzone assembly	GO:0051255	5	1.85169439	2.67E-02
regulation of phospholipid biosynthetic process	GO:0071071	5	1.84703915	2.66E-02
positive regulation of apoptotic process involved in mammary gland involution	GO:0060058	5	1.83129215	3.11E-02
positive regulation of apoptotic process involved in morphogenesis	GO:1902339	5	1.83129215	3.11E-02
regulation of timing of cell differentiation	GO:0048505	5	1.82736193	3.12E-02
membrane depolarization involved in regulation of action potential	GO:0086010	5	1.82421195	3.10E-02
regulation of branching involved in salivary gland morphogenesis	GO:0060693	5	1.80439376	3.35E-02
glial cell migration	GO:0008347	8	1.80437148	8.96E-03
phasic smooth muscle contraction	GO:0014821	5	1.78807981	3.89E-02
positive regulation of blood coagulation	GO:0030194	8	1.77381253	1.10E-02
positive regulation of hemostasis	GO:1900048	8	1.77381253	1.10E-02
regulation of the force of heart contraction by chemical signal	GO:0003057	5	1.76257417	4.36E-02
negative regulation of stem cell differentiation	GO:2000737	5	1.76051147	4.29E-02
gas homeostasis	GO:0033483	6	1.74117242	3.05E-02
arginine metabolic process	GO:0006525	5	1.70666984	4.83E-02
positive chemotaxis	GO:0050918	11	1.68556844	6.40E-03
cellular response to interferon-beta	GO:0035458	19	1.65369254	2.88E-10
regulation of astrocyte differentiation	GO:0048710	11	1.64694622	8.64E-03
interleukin-1 beta production	GO:0032611	9	1.64559499	1.66E-02
adenohypophysis development	GO:0021984	6	1.64033751	4.39E-02
positive regulation of astrocyte differentiation	GO:0048711	7	1.63323471	2.75E-02
lymphocyte costimulation	GO:0031294	10	1.62487873	8.89E-03
T cell costimulation	GO:0031295	10	1.62487873	8.83E-03
positive regulation vascular endothelial growth factor production	GO:0010575	9	1.62390733	1.67E-02
desensitization of G-protein coupled receptor protein signaling pathway	GO:0002029	12	1.61387125	6.78E-03
negative adaptation of signaling pathway	GO:0022401	12	1.61387125	6.78E-03
positive regulation of cardiac muscle cell proliferation	GO:0060045	6	1.60369486	4.82E-02
response to interferon-alpha	GO:0035455	14	1.59967433	3.67E-10
positive regulation of phospholipase C activity	GO:0010863	8	1.58491068	2.42E-02
regulation of phospholipase C activity	GO:1900274	8	1.58491068	2.40E-02
response to interferon-beta	GO:0035456	24	1.58326395	3.36E-10
regulation of interleukin-2 biosynthetic process	GO:0045076	8	1.58046885	2.48E-02
regulation of fibroblast migration	GO:0010762	10	1.57497171	1.31E-02
negative regulation of cellular response to oxidative stress	GO:1900408	7	1.56353177	3.60E-02
cell adhesion mediated by integrin	GO:0033627	7	1.55700412	3.88E-02
negative regulation of protein processing	GO:0010955	10	1.55606608	1.72E-02
regulation of heat generation	GO:0031650	11	1.54916059	1.29E-02
negative regulation of amine transport	GO:0051953	7	1.54738428	4.03E-02
macrophage chemotaxis	GO:0048246	7	1.54305035	4.04E-02

regulation of organ formation	GO:0003156	9	1.54106357	2.15E-02
aorta development	GO:0035904	11	1.54024483	1.28E-02
aorta morphogenesis	GO:0035909	11	1.54024483	1.28E-02
regulation of endothelial cell apoptotic process	GO:2000351	14	1.5401856	1.61E-10
pharyngeal system development	GO:0060037	7	1.53548183	4.02E-02
negative regulation of coagulation	GO:0050819	13	1.5346437	3.91E-03
negative regulation of glial cell differentiation	GO:0045686	9	1.53232753	2.39E-02
negative regulation of purine nucleotide metabolic process	GO:1900543	8	1.51512491	3.49E-02
negative regulation of smooth muscle cell migration	GO:0014912	8	1.50558946	3.51E-02
adaptation of signaling pathway	GO:0023058	13	1.50452924	4.07E-03
negative regulation of organic acid transport	GO:0032891	9	1.5004347	2.87E-02
regulation of fever generation	GO:0031620	8	1.49989427	3.66E-02
ventricular septum morphogenesis	GO:0060412	14	1.49848854	3.70E-03
positive regulation of G-protein coupled receptor protein signaling pathway	GO:0045745	7	1.49435456	4.78E-02
detection of bacterium	GO:0016045	11	1.49420573	1.41E-02
interleukin-1 production	GO:0032612	10	1.4899627	2.07E-02
positive regulation of phospholipase activity	GO:0010518	12	1.48300529	1.42E-02
cyclic purine nucleotide metabolic process	GO:0052652	8	1.4829182	3.93E-02
response to ischemia	GO:0002931	8	1.47724137	4.08E-02
negative regulation of epithelial cell differentiation	GO:0030857	14	1.47113669	4.03E-03
regulation of vascular endothelial growth factor production	GO:0010574	11	1.46648939	1.96E-02
vasoconstriction	GO:0042310	13	1.45870896	6.50E-03
negative regulation of JAK-STAT cascade	GO:0046426	10	1.45783966	2.41E-02
regulation of amino acid transport	GO:0051955	10	1.44536649	2.47E-02
hyaluronan metabolic process	GO:0030212	9	1.43481506	3.59E-02
microglial cell activation	GO:0001774	10	1.4329157	2.50E-02
microtubule polymerization or depolymerization	GO:0031109	11	1.43237133	2.58E-02
regulation of blood coagulation	GO:0030193	32	1.42844751	5.04E-10
regulation of hemostasis	GO:1900046	32	1.42844751	5.04E-10
regulation of coagulation	GO:0050818	36	1.42319117	1.92E-10
regulation of alpha-beta T cell proliferation	GO:0046640	13	1.41798299	1.26E-02
detection of biotic stimulus	GO:0009595	17	1.41624776	6.72E-10
glomerulus development	GO:0032835	14	1.41255304	6.55E-03
somatic stem cell division	GO:0048103	12	1.41184421	2.04E-02
artery morphogenesis	GO:0048844	26	1.40890315	2.12E-10
response to muramyl dipeptide	GO:0032495	10	1.39957499	2.65E-02
negative regulation of interleukin-1 production	GO:0032692	9	1.39508484	4.32E-02
negative regulation of osteoclast differentiation	GO:0045671	10	1.38727009	3.02E-02
negative regulation of nucleotide metabolic process	GO:0045980	9	1.38712705	4.63E-02
positive regulation of coagulation	GO:0050820	11	1.38644099	3.24E-02
positive regulation of interleukin-1 secretion	GO:0050716	13	1.38425849	1.53E-02
positive regulation of interleukin-1 beta secretion	GO:0050718	13	1.38425849	1.53E-02
apoptotic cell clearance	GO:0043277	14	1.38343013	1.28E-02
positive regulation of phosphatidylinositol 3-kinase activity	GO:0043552	13	1.37948806	1.54E-02
filopodium assembly	GO:0046847	11	1.37672041	3.52E-02
negative regulation of blood coagulation	GO:0030195	11	1.37359442	3.53E-02
negative regulation of hemostasis	GO:1900047	11	1.37359442	3.53E-02
artery development	GO:0060840	30	1.37295354	1.75E-10
negative regulation of anoikis	GO:2000811	12	1.37248146	3.01E-02
defense response to Gram-negative bacterium	GO:0050829	14	1.37063247	1.57E-02
cardiac septum morphogenesis	GO:0060411	20	1.36779304	3.73E-03
positive regulation of lipid kinase activity	GO:0090218	14	1.36635818	1.68E-02
defense response to protozoan	GO:0042832	14	1.36346707	1.70E-02
positive regulation of monooxygenase activity	GO:0032770	11	1.35439772	3.97E-02
regulation of monooxygenase activity	GO:0032768	20	1.34316862	6.61E-03
regulation of antigen processing and presentation	GO:0002577	10	1.34258561	4.11E-02
regulation of interleukin-1 secretion	GO:0050704	15	1.34218818	2.16E-02
regulation of cellular response to oxidative stress	GO:1900407	10	1.33774903	4.09E-02

heparan sulfate proteoglycan metabolic process	GO:0030201	11	1.33515951	4.33E-02
outflow tract morphogenesis	GO:0003151	18	1.33018339	9.09E-03
regulation of macrophage derived foam cell differentiation	GO:0010743	13	1.32972081	2.49E-02
negative regulation of viral genome replication	GO:0045071	23	1.3221703	3.95E-03
purinergic receptor signaling pathway	GO:0035587	10	1.32045665	4.79E-02
negative regulation of gliogenesis	GO:0014014	14	1.31998066	2.57E-02
negative regulation of T cell apoptotic process	GO:0070233	10	1.31947952	4.76E-02
smooth muscle contraction	GO:0006939	17	1.30742987	1.43E-02
negative regulation of oxidoreductase activity	GO:0051354	12	1.30276356	4.41E-02
regulation of smooth muscle cell migration	GO:0014910	24	1.29920422	4.11E-03
regulation of wound healing	GO:0061041	44	1.29446349	1.04E-07
regulation of interleukin-1 beta secretion	GO:0050706	14	1.29265211	3.22E-02
regulation of platelet activation	GO:0010543	15	1.29166435	2.76E-02
neural crest cell development	GO:0014032	20	1.29048701	1.30E-02
negative regulation of smooth muscle cell proliferation	GO:0048662	20	1.29029283	1.25E-02
positive regulation of tissue remodeling	GO:0034105	14	1.28832682	3.23E-02
positive regulation of lipase activity	GO:0060193	17	1.28821145	1.69E-02
heterophilic cell-cell adhesion	GO:0007157	16	1.28714245	1.85E-02
positive regulation of smooth muscle cell migration	GO:0014911	16	1.2857882	1.83E-02
positive regulation of interleukin-1 beta production	GO:0032731	15	1.28150856	3.06E-02
cellular response to monosaccharide stimulus	GO:0071326	13	1.27964886	3.58E-02
cellular response to hexose stimulus	GO:0071331	13	1.27964886	3.58E-02
cellular response to glucose stimulus	GO:0071333	13	1.27964886	3.57E-02
regulation of nitric-oxide synthase activity	GO:0050999	15	1.27138217	3.31E-02
regulation of interleukin-1 production	GO:0032652	24	1.2705207	3.99E-03
regulation of phospholipase activity	GO:0010517	16	1.26531156	1.84E-02
receptor internalization	GO:0031623	22	1.26463201	1.59E-02
regulation of interleukin-1 beta production	GO:0032651	22	1.25591578	1.58E-02
positive regulation of interleukin-1 production	GO:0032732	16	1.2530803	2.18E-02
lamellipodium assembly	GO:0030032	22	1.25228192	1.71E-02
negative regulation of viral process	GO:0048525	32	1.24760072	2.37E-10
glycosaminoglycan biosynthetic process	GO:0006024	23	1.24617625	6.72E-03
response to protozoan	GO:0001562	16	1.2432103	2.20E-02
positive regulation of angiogenesis	GO:0045766	53	1.237824	1.65E-08
negative regulation of signal transduction in absence of ligand	GO:1901099	17	1.23560817	2.03E-02
negative regulation of extrinsic apoptotic signaling pathway in absence of ligand	GO:2001240	17	1.23560817	2.03E-02
cellular glucose homeostasis	GO:0001678	20	1.23392935	1.98E-02
positive regulation of cell-cell adhesion	GO:0022409	21	1.23340537	1.94E-02
neural crest cell differentiation	GO:0014033	24	1.23308211	6.66E-03
regulation of phosphatidylinositol 3-kinase activity	GO:0043551	18	1.23162149	2.06E-02
phagocytosis, engulfment	GO:0006911	13	1.22671454	4.75E-02
ventricular septum development	GO:0003281	23	1.22221709	9.03E-03
regulation of tissue remodeling	GO:0034103	26	1.22221225	4.03E-10
positive regulation of phosphatidylinositol 3-kinase cascade	GO:0014068	26	1.22201328	5.76E-10
regulation of interleukin-2 production	GO:0032663	26	1.22122969	4.48E-10
positive regulation of cytokine secretion	GO:0050715	39	1.22048034	2.02E-10
regulation of lipid kinase activity	GO:0043550	23	1.21252271	1.09E-02
cellular response to oxygen levels	GO:0071453	27	1.21199759	1.68E-10
regulation of natural killer cell activation	GO:0032814	16	1.21044331	2.56E-02
regulation of glial cell differentiation	GO:0045685	25	1.20673149	1.27E-02
pituitary gland development	GO:0021983	15	1.20623686	4.40E-02
regulation of morphogenesis of a branching structure	GO:0060688	20	1.20343787	2.51E-02
glycosaminoglycan metabolic process	GO:0030203	40	1.2019579	1.04E-05
retina vasculature development in camera-type eye	GO:0061298	16	1.19703292	2.88E-02
negative regulation of endothelial cell proliferation	GO:0001937	18	1.19108607	3.62E-02
regulation of amine transport	GO:0051952	26	1.19035294	3.77E-03
icosanoid biosynthetic process	GO:0046456	19	1.18859861	2.68E-02

fatty acid derivative biosynthetic process	GO:1901570	19	1.18859861	2.68E-02
proteoglycan metabolic process	GO:0006029	32	1.18554662	1.34E-09
regulation of cytokine secretion	GO:0050707	53	1.18437806	4.62E-07
brown fat cell differentiation	GO:0050873	18	1.18325613	3.95E-02
prostanoid metabolic process	GO:0006692	16	1.18096292	4.18E-02
prostaglandin metabolic process	GO:0006693	16	1.18096292	4.18E-02
negative regulation of lymphocyte apoptotic process	GO:0070229	18	1.17736889	3.94E-02
macrophage activation	GO:0042116	20	1.17706123	3.14E-02
aminoglycan biosynthetic process	GO:0006023	25	1.17305401	1.55E-02
regulation of tyrosine phosphorylation of STAT protein	GO:0042509	22	1.1727295	3.26E-02
regulation of anoikis	GO:2000209	16	1.17232654	4.53E-02
interaction with symbiont	GO:0051702	26	1.17147033	6.35E-03
regulation of oligodendrocyte differentiation	GO:0048713	16	1.16597172	4.54E-02
regulation of protein processing	GO:0070613	19	1.15687684	3.68E-02
membrane depolarization	GO:0051899	29	1.15677405	1.38E-02
cellular response to carbohydrate stimulus	GO:0071322	17	1.15310757	3.98E-02
receptor metabolic process	GO:0043112	38	1.15013567	2.69E-10
leukocyte cell-cell adhesion	GO:0007159	23	1.14679986	1.97E-02
icosanoid metabolic process	GO:0006690	34	1.14156236	1.01E-09
fatty acid derivative metabolic process	GO:1901568	34	1.14156236	1.01E-09
mesenchymal cell development	GO:0014031	40	1.14012766	1.51E-04
proteoglycan biosynthetic process	GO:0030166	22	1.13758747	4.34E-02
lipid storage	GO:0019915	19	1.13731012	4.28E-02
regulation of cell adhesion mediated by integrin	GO:0033628	21	1.13686321	4.32E-02
defense response to Gram-positive bacterium	GO:0050830	29	1.13565644	1.39E-02
cardiac septum development	GO:0003279	31	1.13457165	1.44E-02
regulation of oxidoreductase activity	GO:0051341	35	1.13374991	3.88E-03
regulation of extrinsic apoptotic signaling pathway via death domain receptors	GO:1902041	26	1.13237473	1.86E-02
smooth muscle cell differentiation	GO:0051145	20	1.13236348	4.31E-02
positive regulation of peptidyl-tyrosine phosphorylation	GO:0050731	64	1.13030795	8.45E-07
positive regulation of interferon-gamma production	GO:0032729	19	1.1288437	4.26E-02
telencephalon cell migration	GO:0022029	28	1.12424046	1.95E-02
cell chemotaxis	GO:0060326	65	1.12327057	1.32E-06
defense response to bacterium	GO:0042742	68	1.12237167	1.36E-06
cytosolic calcium ion transport	GO:0060401	26	1.12024002	1.93E-02
calcium ion transport into cytosol	GO:0060402	26	1.12024002	1.92E-02
unsaturated fatty acid metabolic process	GO:0033559	35	1.11979547	8.70E-03
regulation of viral genome replication	GO:0045069	38	1.11541304	2.52E-10
positive regulation of endothelial cell proliferation	GO:0001938	28	1.11164002	2.29E-02
forebrain cell migration	GO:0021885	29	1.11142904	2.43E-02
lymphocyte homeostasis	GO:0002260	39	1.10987534	2.02E-09
aminoglycan metabolic process	GO:0006022	45	1.10976175	1.32E-04
cellular response to molecule of bacterial origin	GO:0071219	90	1.10744732	2.18E-08
somatic stem cell maintenance	GO:0035019	25	1.10715799	2.70E-02
regulation of gliogenesis	GO:0014013	41	1.10380447	6.01E-04
regulation of acute inflammatory response	GO:0002673	30	1.10290915	2.44E-02
patterning of blood vessels	GO:0001569	23	1.0991838	3.34E-02
leukocyte homeostasis	GO:0001776	45	1.09808258	2.06E-04
renal system process	GO:0003014	25	1.09652708	3.32E-02
cellular response to lipopolysaccharide	GO:0071222	83	1.09499753	2.58E-07
positive regulation of cell division	GO:0051781	37	1.09419527	3.84E-03
positive regulation of protein secretion	GO:0050714	52	1.09318081	5.87E-05
leukocyte chemotaxis	GO:0030595	53	1.09001312	6.81E-05
calcium-mediated signaling	GO:0019722	29	1.08984884	2.89E-02
regulation of sodium ion transport	GO:0002028	27	1.08703311	2.53E-02
lipopolysaccharide-mediated signaling pathway	GO:0031663	24	1.08374547	4.17E-02
leukocyte migration	GO:0050900	67	1.08298976	1.40E-05

negative regulation of multi-organism process	GO:0043901	47	1.07964065	4.42E-04
positive regulation of chemotaxis	GO:0050921	41	1.07828895	1.47E-03
cellular response to decreased oxygen levels	GO:0036294	25	1.07636415	4.36E-02
cellular response to hypoxia	GO:0071456	25	1.07636415	4.36E-02
negative regulation of G-protein coupled receptor protein signaling pathway	GO:0045744	35	1.07609625	2.05E-02
positive regulation of phagocytosis	GO:0050766	29	1.07578421	3.16E-02
negative regulation of angiogenesis	GO:0016525	34	1.07459503	2.08E-02
regulation of chemotaxis	GO:0050920	54	1.07307735	1.53E-04
regulation of smooth muscle cell proliferation	GO:0048660	59	1.07195758	2.24E-10
regulation of vasculature development	GO:1901342	99	1.07129358	3.82E-08
wound healing	GO:0042060	96	1.06917316	4.48E-08
negative regulation of neurogenesis	GO:0050768	51	1.06877751	1.83E-04
regulation of Notch signaling pathway	GO:0008593	28	1.0647536	3.08E-02
regulation of G-protein coupled receptor protein signaling pathway	GO:0008277	58	1.06365111	8.06E-10
cardiac ventricle morphogenesis	GO:0003208	27	1.06339383	3.09E-02
regulation of angiogenesis	GO:0045765	92	1.0622858	7.50E-07
regulation of phagocytosis	GO:0050764	39	1.06014016	6.45E-03
regulation of endothelial cell proliferation	GO:0001936	42	1.05752909	2.93E-03
positive regulation of T cell proliferation	GO:0042102	43	1.05373122	3.30E-03
mesenchyme development	GO:0060485	54	1.0531761	3.66E-04
regulation of leukocyte chemotaxis	GO:0002688	33	1.05265397	2.59E-02
positive regulation of epithelial cell proliferation	GO:0050679	63	1.0494388	8.66E-05
regulation of phosphatidylinositol 3-kinase cascade	GO:0014066	34	1.04836931	3.07E-02
regulation of interferon-gamma production	GO:0032649	39	1.04665532	1.10E-02
positive regulation of behavior	GO:0048520	46	1.04602725	1.58E-03
positive regulation of cytokine biosynthetic process	GO:0042108	35	1.04505194	3.03E-02
regulation of organic acid transport	GO:0032890	26	1.04070471	4.37E-02
regulation of peptidyl-tyrosine phosphorylation	GO:0050730	93	1.0403905	5.03E-07
regeneration	GO:0031099	26	1.03953186	4.39E-02
negative regulation of leukocyte apoptotic process	GO:2000107	28	1.03879234	4.34E-02
positive regulation of fibroblast proliferation	GO:0048146	27	1.03722693	4.35E-02
negative regulation of cell activation	GO:0050866	69	1.03712548	3.44E-05
regulation of behavior	GO:0050795	72	1.03405774	4.10E-05
cardiac ventricle development	GO:0003231	47	1.03365052	2.48E-03
response to interferon-gamma	GO:0034341	31	1.03337094	4.47E-02
regulation of blood vessel size	GO:0050880	32	1.03327567	3.42E-02
regulation of protein secretion	GO:0050708	81	1.03206957	1.74E-05
vascular process in circulatory system	GO:0003018	42	1.03133669	5.57E-03
granulocyte chemotaxis	GO:0071621	27	1.03016953	4.69E-02
response to hypoxia	GO:0001666	89	1.02931121	7.73E-06
response to decreased oxygen levels	GO:0036293	89	1.02931121	7.55E-06
regulation of lipase activity	GO:0060191	33	1.02800091	3.40E-02
myeloid leukocyte activation	GO:0002274	62	1.02741805	2.57E-04
phagocytosis	GO:0006909	53	1.0269779	1.13E-03
regulation of striated muscle contraction	GO:0006942	26	1.02611569	4.73E-02
cytokine production	GO:0001816	61	1.02476146	4.16E-03
mesenchymal cell differentiation	GO:0048762	47	1.02287118	3.57E-03
negative regulation of extrinsic apoptotic signaling pathway	GO:2001237	58	1.02239401	3.63E-03
cardiac chamber morphogenesis	GO:0003206	45	1.02156775	3.63E-03
negative regulation of ion transport	GO:0043271	32	1.02028182	4.01E-02
elevation of cytosolic calcium ion concentration	GO:0007204	64	1.019026	3.84E-04
positive regulation of leukocyte migration	GO:0002687	39	1.01818581	2.19E-02
negative regulation of locomotion	GO:0040013	99	1.01657805	3.17E-06
defense response to virus	GO:0051607	86	1.01398531	2.04E-05
receptor-mediated endocytosis	GO:0006898	50	1.01297134	1.89E-03
regulation of tumor necrosis factor production	GO:0032680	59	1.00845408	8.76E-03
regulation of tube size	GO:0035150	33	1.00714782	4.38E-02
negative regulation of cell migration	GO:0030336	86	1.00681432	3.30E-05

cytosolic calcium ion homeostasis	GO:0051480	72	1.00678206	1.84E-04
regulation of mononuclear cell proliferation	GO:0032944	94	1.00467577	7.95E-06
regulation of lymphocyte proliferation	GO:0050670	94	1.00467577	7.75E-06
regulation of T cell proliferation	GO:0042129	66	1.00418132	7.84E-04
regulation of cell-cell adhesion	GO:0022407	52	1.00410483	2.66E-03
regulation of JAK-STAT cascade	GO:0046425	42	1.00246248	1.18E-02
negative regulation of lymphocyte activation	GO:0051250	55	1.00102033	2.97E-03
positive regulation of osteoblast differentiation	GO:0045669	37	1.00047764	3.25E-02
regulation of leukocyte proliferation	GO:0070663	96	0.99863679	1.13E-05
positive regulation of ERK1 and ERK2 cascade	GO:0070374	46	0.99549466	6.99E-03
second-messenger-mediated signaling	GO:0019932	44	0.99518733	1.38E-02
positive regulation of mononuclear cell proliferation	GO:0032946	64	0.99381503	1.23E-03
positive regulation of lymphocyte proliferation	GO:0050671	64	0.99381503	1.22E-03
negative regulation of leukocyte activation	GO:0002695	65	0.99030115	1.43E-03
blood coagulation	GO:0007596	48	0.98629712	8.97E-03
coagulation	GO:0050817	48	0.98629712	8.97E-03
positive regulation of leukocyte proliferation	GO:0070665	66	0.98516583	1.75E-03
negative regulation of T cell activation	GO:0050868	42	0.97880502	1.88E-02
negative regulation of cell motility	GO:2000146	89	0.97852952	1.85E-04
regulation of cell-matrix adhesion	GO:0001952	40	0.97828192	1.90E-02
regulation of viral process	GO:0050792	57	0.97756479	1.40E-02
regulation of symbiosis, encompassing mutualism through parasitism	GO:0043903	68	0.97696074	2.50E-03
negative regulation of inflammatory response	GO:0050728	51	0.97456754	6.54E-03
neuron death	GO:0070997	40	0.97418568	2.07E-02
regulation of fibroblast proliferation	GO:0048145	47	0.97358037	1.31E-02
activation of MAPK activity	GO:0000187	58	0.97333735	1.45E-02
negative regulation of cell development	GO:0010721	59	0.97331087	1.41E-02
positive regulation of endocytosis	GO:0045807	55	0.97134923	6.81E-03
hemostasis	GO:0007599	50	0.96743147	7.84E-03
regulation of B cell activation	GO:0050864	61	0.96561841	1.53E-02
positive regulation of inflammatory response	GO:0050729	44	0.96466697	2.48E-02
regulation of ion homeostasis	GO:2000021	67	0.96430655	4.00E-03
regulation of muscle contraction	GO:0006937	47	0.96378281	1.59E-02
calcium ion transmembrane transport	GO:0070588	45	0.96308672	1.55E-02
memory	GO:0007613	40	0.96296743	2.51E-02
positive regulation of T cell activation	GO:0050870	81	0.96287382	8.79E-04
extracellular matrix organization	GO:0030198	64	0.96257971	4.24E-03
extracellular structure organization	GO:0043062	64	0.96257971	4.19E-03
positive regulation of protein kinase B signaling cascade	GO:0051897	41	0.96086614	2.60E-02
negative regulation of secretion	GO:0051048	64	0.96022667	3.97E-03
cardiac chamber development	GO:0003205	57	0.95917123	1.56E-02
regulation of interleukin-6 production	GO:0032675	54	0.95889421	9.96E-03
ameboidal cell migration	GO:0001667	64	0.953877	5.15E-03
regulation of leukocyte migration	GO:0002685	52	0.95182577	1.25E-02
T cell differentiation	GO:0030217	78	0.95097014	1.53E-03
negative regulation of cellular component movement	GO:0051271	93	0.9491089	2.85E-04
regulation of ERK1 and ERK2 cascade	GO:0070372	70	0.94481155	3.54E-03
heart morphogenesis	GO:0003007	87	0.94462516	1.18E-03
negative regulation of response to external stimulus	GO:0032102	80	0.94455292	2.07E-03
positive regulation of ion transport	GO:0043270	69	0.94450671	3.55E-03
regulation of reactive oxygen species metabolic process	GO:2000377	45	0.94332425	2.42E-02
antigen processing and presentation	GO:0019882	46	0.93572608	2.72E-02
calcium ion transport	GO:0006816	97	0.93464881	6.82E-04
regulation of organ morphogenesis	GO:2000027	62	0.93390949	9.97E-03
tissue remodeling	GO:0048771	54	0.93362201	1.86E-02
regulation of lymphocyte mediated immunity	GO:0002706	54	0.93298011	1.89E-02
positive regulation of homeostatic process	GO:0032846	40	0.93166405	4.41E-02
negative regulation of defense response	GO:0031348	66	0.93013213	1.12E-02

positive regulation of response to external stimulus	GO:0032103	97	0.92732363	1.04E-03
regulation of cell-substrate adhesion	GO:0010810	68	0.92679376	1.26E-02
positive regulation of cell adhesion	GO:0045785	81	0.92637622	3.89E-03
regulation of leukocyte apoptotic process	GO:2000106	50	0.92587974	2.15E-02
regulation of muscle system process	GO:0090257	66	0.92196161	1.43E-02
regulation of cell division	GO:0051302	54	0.91572127	2.66E-02
cardiocyte differentiation	GO:0035051	47	0.91514586	4.09E-02
immune response-regulating signaling pathway	GO:0002764	86	0.91460111	3.98E-03
regulation of body fluid levels	GO:0050878	90	0.91142075	4.67E-03
negative regulation of cell projection organization	GO:0031345	47	0.90978425	4.47E-02
regulation of heart contraction	GO:0008016	54	0.90805482	3.18E-02
negative regulation of cell adhesion	GO:0007162	59	0.90794509	4.72E-02
sulfur compound biosynthetic process	GO:0044272	45	0.90585046	4.80E-02
regulation of endocytosis	GO:0030100	97	0.90427998	3.33E-03
cell-substrate adhesion	GO:0031589	60	0.90312668	4.70E-02
regulation of leukocyte mediated immunity	GO:0002703	74	0.90171409	1.44E-02
gland morphogenesis	GO:0022612	50	0.90011326	3.77E-02
positive regulation of defense response	GO:0031349	97	0.8994649	4.12E-03
negative regulation of protein serine/threonine kinase activity	GO:0071901	69	0.89651334	1.60E-02
immune response-activating signal transduction	GO:0002757	78	0.88913288	1.49E-02
regulation of adaptive immune response	GO:0002819	74	0.88467863	2.16E-02
activation of immune response	GO:0002253	100	0.87999123	8.63E-03
kidney development	GO:0001822	73	0.87867856	2.54E-02
regulation of cell shape	GO:0008360	68	0.8783082	3.94E-02
regulation of calcium ion transport	GO:0051924	75	0.87545011	2.73E-02
detection of stimulus	GO:0051606	65	0.87363064	4.28E-02
cognition	GO:0050890	88	0.87175657	1.89E-02
adaptive immune response based on somatic recombination of immune receptors built from immunoglobulin superfamily domains	GO:0002460	71	0.86726646	3.36E-02
regulation of cellular response to growth factor stimulus	GO:0090287	85	0.86701514	2.15E-02
cardiac muscle tissue development	GO:0048738	65	0.86693474	4.94E-02
regulation of muscle tissue development	GO:1901861	64	0.86670159	4.96E-02
response to steroid hormone stimulus	GO:0048545	98	0.86577141	1.45E-02
symbiosis, encompassing mutualism through parasitism	GO:0044403	83	0.86297017	3.01E-02
interspecies interaction between organisms	GO:0044419	83	0.86297017	3.01E-02
positive regulation of immune effector process	GO:0002699	77	0.85839524	3.32E-02
MAPK cascade	GO:0000165	97	0.85468287	1.96E-02
ear development	GO:0043583	80	0.85326217	3.86E-02
morphogenesis of a branching epithelium	GO:0061138	92	0.85219359	3.18E-02
cytokine-mediated signaling pathway	GO:0019221	95	0.85114406	2.16E-02
branching morphogenesis of an epithelial tube	GO:0048754	80	0.85070051	4.10E-02
placenta development	GO:0001890	85	0.84767868	3.56E-02
morphogenesis of a branching structure	GO:0001763	93	0.84610506	2.52E-02
learning or memory	GO:0007611	83	0.84586836	4.51E-02
telencephalon development	GO:0021537	86	0.84104647	4.31E-02
development of primary sexual characteristics	GO:0045137	88	0.8384288	4.51E-02
regulation of innate immune response	GO:0045088	93	0.82217568	4.90E-02
